# Covalent labeling of a chromatin reader domain using proximity-reactive cyclic peptides†

**DOI:** 10.1039/d2sc00555g

**Published:** 2022-05-12

**Authors:** Meng Yao Zhang, Hyunjun Yang, Gloria Ortiz, Michael J. Trnka, Nektaria Petronikolou, Alma L. Burlingame, William F. DeGrado, Danica Galonić Fujimori

**Affiliations:** Department of Cellular and Molecular Pharmacology, University of California San Francisco San Francisco CA 94158 USA danica.fujimori@ucsf.edu; Department of Pharmaceutical Chemistry, University of California San Francisco San Francisco CA 94158 USA; Quantitative Biosciences Institute, University of California San Francisco San Francisco CA 94158 USA

## Abstract

Chemical probes for chromatin reader proteins are valuable tools for investigating epigenetic regulatory mechanisms and evaluating whether the target of interest holds therapeutic potential. Developing potent inhibitors for the plant homeodomain (PHD) family of methylation readers remains a difficult task due to the charged, shallow and extended nature of the histone binding site that precludes effective engagement of conventional small molecules. Herein, we describe the development of novel proximity-reactive cyclopeptide inhibitors for PHD3—a trimethyllysine reader domain of histone demethylase KDM5A. Guided by the PHD3–histone co-crystal structure, we designed a sidechain-to-sidechain linking strategy to improve peptide proteolytic stability whilst maintaining binding affinity. We have developed an operationally simple solid-phase macrocyclization pathway, capitalizing on the inherent reactivity of the dimethyllysine ε-amino group to generate scaffolds bearing charged tetraalkylammonium functionalities that effectively engage the shallow aromatic ‘groove’ of PHD3. Leveraging a surface-exposed lysine residue on PHD3 adjacent to the ligand binding site, cyclic peptides were rendered covalent through installation of an arylsulfonyl fluoride warhead. The resulting lysine-reactive cyclic peptides demonstrated rapid and efficient labeling of the PHD3 domain in HEK293T lysates, showcasing the feasibility of employing proximity-induced reactivity for covalent labeling of this challenging family of reader domains.

## Abstract 2

We describe the development of covalent cyclic peptide ligands which target a chromatin methylation reader domain using a proximity-reactive sulfonyl fluoride moiety.

## Graphical abstract

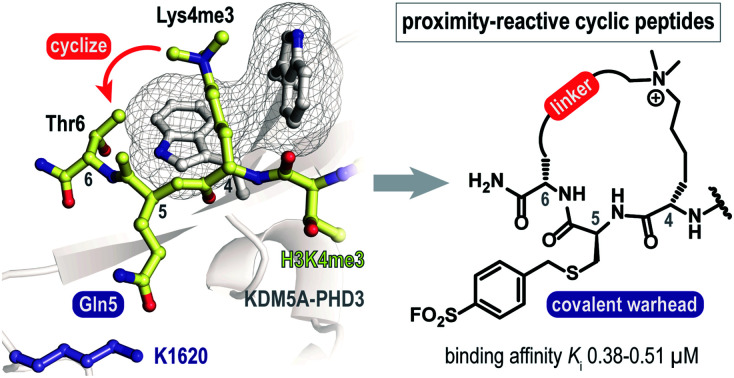

## Introduction

**Fig. 1:**
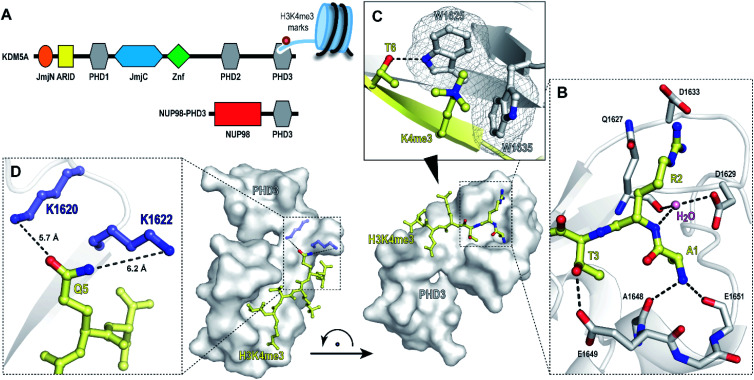
PHD3 domain of histone demethylase KDM5A binds histone H3 tail trimethylated at Lys4 (H3K4me3). (A) Domain architecture of KDM5A and oncoprotein NUP98–PHD3. (B and C) Crystal structure of KDM5A-PHD3 (grey) complexed with the H3K4me3 peptide (yellow). PDB 3GL6.^7^ (D) Solvent-exposed K1620 and K1622 of PHD3 are positioned adjacent to the Q5 residue upon H3K4me3 binding.

Chromatin reader domains recognize chromatin as a function of the site and the extent of histone modification. Plant homeodomains (PHD) are a class of chromatin readers that selectively associate with lysine residues with varying methylation states.^1,2^ While electrostatic interactions drive recognition of unmodified lysine residues, methyllysine-selective reader domains utilize an aromatic ‘cage’—formed through perpendicular positioning of aromatic amino acids to facilitate cation-π interactions to engage lysines with high methylation states.^1^ Misregulation of PHD domains is associated with pathogenesis of human diseases, including cancer, immunodeficiency and neurological disorders.^3–6^ Chromosomal translocation of PHD3—the C-terminal H3K4me2/3-specific PHD domain of histone demethylase KDM5A, with nucleoporin-98 (NUP98), has been reported to induce malignancy in acute myeloid leukemia (Fig. 1A).^7^ Specifically, the resulting oncogenic PHD3–NUP98 fusion aberrantly recruits NUP98 to H3K4me3 marks, preventing the silencing of critical transcription factors during hematopoietic differentiation. Interestingly, an alternatively spliced isoform lacking the PHD3 reader module failed to induce leukemia, suggesting that a functional PHD3 domain is necessary for the oncogenic effects of NUP98–PHD3 fusions.^7^

While the repertoire of chemical probes for various classes of reader domains has significantly increased in recent years,^8–15^ thus far, only a few small molecule ligands have been described for PHD domains, owing to their extended, solvent-exposed histone binding site.^16–19^ To date, there have been several reports on the development of small-molecule antagonists for the PHD3 finger of KDM5A. In 2012, Wagner and co-workers identified analogues of amiodarone as PHD3 inhibitors through a HaloTag screen.^20^ Unfortunately, subsequent structure–activity relationship (SAR) studies revealed insufficient selectivity for the PHD3 finger, underscored by promiscuous binding to PHD domains of KDM7 and inhibition of KDM5A catalytic activity.^21^ Developing selective and potent probes for PHD fingers remains a formidable challenge, highlighting the increasing demand for better-quality tool compounds to study this family of epigenetic reader proteins.

The PHD3–H3K4me3 co-crystal structure depicts a charged, large, and shallow binding surface, posing challenges in target modulation using conventional small molecule-based ligand discovery methods (Fig. 1B–D). Peptidomimetics, on the other hand, offer a unique pharmaceutical space. Structurally, they are well suited for capturing extensive protein–protein interactions that lack binding pockets and cover a large surface area. Encouraged by reports of the successful development of peptide-derived inhibitors for CBX proteins,^22–26^ we set to target the PHD3 domain using peptide-based modalities.

The majority of trimethyllysine-binding PHD domains contain an aromatic cage comprised of a combination of two to four aromatic and hydrophobic residues.^1^ Notably, the aromatic cage of the PHD3 domain contains only two tryptophan sidechains (Fig. 1C), giving rise to a shallow aromatic ‘groove’ which engages the trimethyllysine sidechain along the protein surface.^7^ We viewed this unique feature as an opportunity to design PHD3-targeting macrocyclic peptides by tethering the sidechains of Lys4 and Thr6. In addition, we identified two solvent-exposed lysines—K1620 and K1622, which are adjacently positioned to the Q5 sidechain upon ligand binding (Fig. 1D), revealing potential for the development of covalent peptidomimetic ligands. Sequence alignment of the PHD3 domain with other H3K4me3 readers suggested that these lysine residues are not conserved in the majority of other PHD reader domains (Fig. S1†), presenting an opportunity for the development of cyclic peptide ligands selective for the PHD3 domain of KDM5A.

Herein, we describe the structure-guided design of covalent peptide ligands for the PHD3 domain of KDM5A. Based on the known crystal structure of the PHD3 finger, thorough structure–activity relationship studies revealed macrocyclic peptides that inhibited the PHD3–H3K4me3 interaction with sub-micromolar potency. Moreover, the identified cyclopeptide PHD3 ligands were rendered covalent by introduction of a lysine-reactive covalent warhead. To the best of our knowledge, this work demonstrates covalent modulation of a PHD reader domain for the first time, laying the groundwork for future applications of covalent peptides as occupancy probes for PHD3 domain-targeting ligand discovery.

## Results and discussion

### Alanine and truncated peptide scan

**Fig. 2:**
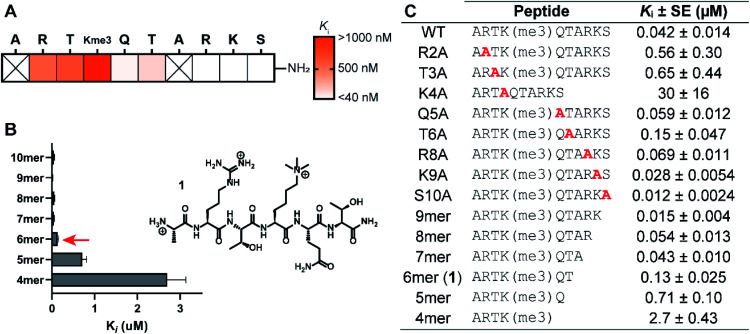
Structure–activity relationship study of the H3K4me3 10mer peptide. (A) Wild-type histone residues were replaced with Ala in the alanine scan. (B) C-terminal truncation scan. (C) Inhibition constants (*K*_i_) of mutant/truncated H3K4me3 peptides measured using competitive FP assays. Data are represented as average ± three standard errors.

The structure-guided design of PHD3 peptide ligands commenced with identifying the minimal binding sequence, achieved through systematic mutagenesis and truncation scanning experiments on the wild type H3K4me3 10mer peptide. Inhibition constants (*K*_i_) of mutant and truncated peptides were determined using an *in vitro* competition-based fluorescence polarization (FP) assay. Briefly, recombinant His_6_-MBP-PHD3 protein was incubated with the 10mer H3K4me3 tracer containing a C-terminal fluorescent label, followed by treatment with varying concentrations of the competing peptide to provide *K*_i_ values (see ESI†). Alanine substitutions demonstrated the importance of N-terminal amino acid sidechains (R2, T3 and K4), where mutations at these hot-spot residues significantly reduced activity (Fig. 2A and C). Interestingly, the Q5A mutation did not compromise binding affinity. Diminished competition was observed with H3K4me3 peptides truncated at the Q5 and T6 positions (Fig. 2B and C), suggesting the presence of critical amide backbone interactions stabilizing the three-stranded β-sheet motif formed upon H3 peptide binding (Fig. 1C). Our systematic scanning of the native sequence revealed hexapeptide ARTK(me3)QT (1), which engaged the PHD3 domain with sub-micromolar binding affinity (*K*_i_ = 0.13 ± 0.025 μM) as a starting point for the rational design of H3 peptide ligands (Fig. 2C).

### Structure–activity relationship studies of the histone peptide

**Fig. 3:**
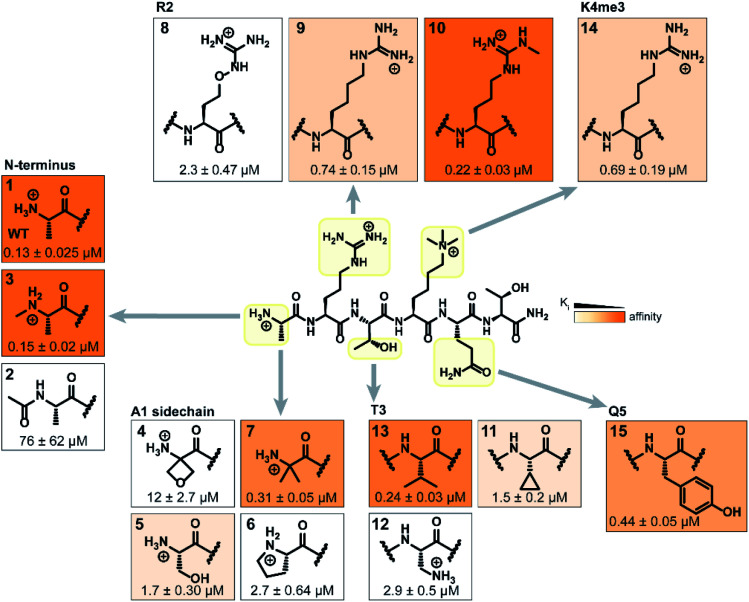
Structure–activity relationship study of modified H3 6mer peptides. Structures of the incorporated natural/unnatural amino acids are boxed. The color tones represent the binding affinity to recombinant His_6_-MBP-PHD3, determined by competitive FP assays.

Binding interaction between the PHD3 domain and H3K4me3 is established *via* the formation of an anti-parallel β-sheet between the H3 backbone and the two-stranded β-sheet of PHD3, which engages the trimethyllysine moiety with a shallow aromatic cleft comprised of W1625 and W1635 through cation-π interactions (Fig. 1C). The aromatic cage significantly contributes to trimethyllysine selectivity, indicated by diminished binding upon mutations of the tryptophan residues.^7^ Additionally, the N-terminus of the H3K4me3 peptide caps the α-helix through stabilization of the backbone carbonyls of E1651 and A1648, and the R2 sidechain is embedded in an acidic pocket consisting of D1629 and D1633 (Fig. 1B). To investigate the tolerance for structural modification across the blueprint H3K4me3 6mer peptide, we conducted a systematic SAR screen, involving N-terminal functionalization and incorporation of proteogenic or non-proteogenic amino acids (Fig. 3).

As expected, N-terminal acetylation of the H3K4me3 peptide (2, Fig. 3) resulted in a significant reduction in binding, highlighting the importance of the protonated free N-terminal amine which engages the α-helix carbonyls of the PHD3 domain through electrostatic interactions. In contrast, N-terminal methylation of the H3K4me3 peptide (3, Fig. 3) was well tolerated. Modification of the A1 sidechain (4–6, Fig. 3) generally lowered binding affinity of the H3 peptide, except for the 2-aminoisobutyric acid-containing peptide 7 which showed comparable binding affinity to the wild type peptide. Replacement of the R2 residue with canavanine substantially reduced binding (8, *K*_i_ = 2.3 μM) compared to the wild type peptide, while substitution with *homo*-arginine led to approximately three-fold decrease in binding affinity (9, *K*_i_ = 0.74 μM). Although structurally similar, the electronegative oxygen atom dramatically decreases the p*K*_a_ value of the oxyguanidino group (p*K*_a_ = 7.01) compared to the guanidino group of arginine and *homo*-arginine (p*K*_a_ ∼ 12.48), suggesting a strong preference for highly basic guanidinium-containing sidechains that enable extensive interactions with the surrounding acidic residues.^27^ Monomethylation of the guanidino group of R2 (10) was well-tolerated, fully retaining binding affinity of the wild type peptide. Interestingly, methylation at the N-terminus and the R2 sidechain improved tryptic stability compared to the wild type peptide (peptides 3 and 10 respectively, Fig. S2a–c†). Modification of the T3 residue to cyclopropyl glycine (11) and 2,3-diaminopropionic acid (12) decreased binding affinity, where the T3V mutation (13) resulted in a slight increase in binding affinity. Replacement of the K4me3 sidechain with *homo*-arginine led to a slight decrease in binding affinity (14, *K*_i_ = 0.69 μM). We postulate that the guanidinium group is also capable of participating in cation-π interactions with the W1625–W1635 aromatic cleft, albeit to a lesser extent than the trimethyllysine functionality. Replacement of Q5 with tyrosine (15) showed comparable activity to the wild type H3K4me3 peptide.

Consistent with the alanine scan, the point mutant SAR studies revealed R2 and K4me3 sidechains as key interactors responsible for the binding of H3K4me3 6mer peptides to the PHD3 domain. Among the modifications evaluated, N-terminal and R2 sidechain methylation, substitution of the T3 residue to valine had the least impact on binding affinity. Additionally, we found that the Q5 position was highly tolerant to substitution, both with smaller (Ala, Fig. 2A) and larger (Tyr, 15, Fig. 3) residues, opening prospects for further modification at this site to incorporate lysine-reactive handles.

### Macrocyclization of the histone hexapeptide

**Fig. 4:**
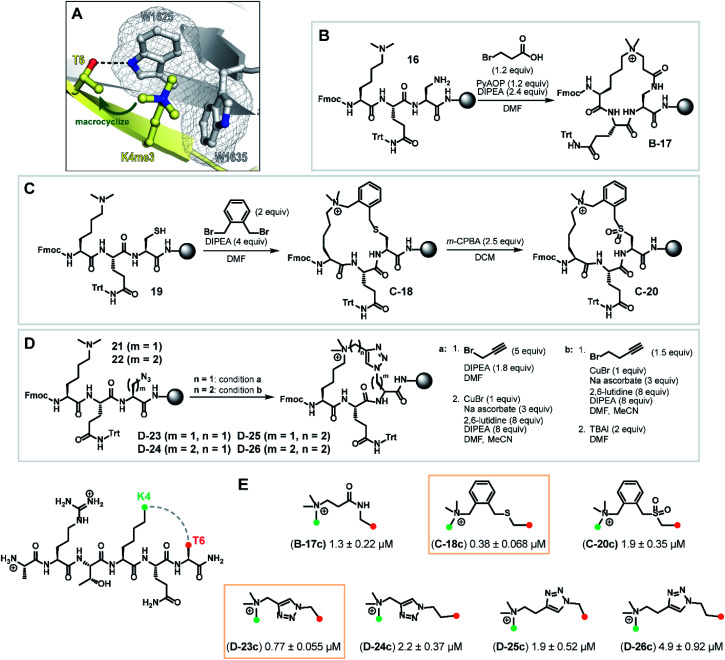
Synthesis of macrocyclic H3K4me3 6mer peptides. (A) Crystal structure of H3K4me3 peptide bound to PHD3 (PDB 3GL6)^7^ highlighting residues Lys4 and Thr6 selected for tethering. (B) Series B: macrolactamization through a tandem amide coupling-intramolecular S_N_2 reaction sequence to afford lactam tripeptide intermediate B-17. (C) Series C: cysteine–dimethyllysine stapling strategy which enabled the preparation of thioether tripeptide intermediate C-18 and sulfone tripeptide intermediate C-20 from linear precursor 19. (D) Series D: macrocyclic triazole-containing peptide intermediates D-23–26, forged through Cu(i)-catalyzed alkyne–azide cycloaddition chemistry. (E) Competitive FP assay data of full-length, cleaved macrocyclic peptides. Structure of linkers are shown.

Macrocyclization is a powerful approach to advance peptide leads through structural rigidification—affording peptide-mimicking compounds with enhanced cell permeability and resistance towards proteolysis.^14,28–30^ After observing rapid degradation of the wild type H3K4me3 hexapeptide with trypsin, we investigated a sidechain-to-sidechain macrocyclization strategy to increase proteolytic stability whilst maintaining the β-sheet backbone interaction with the PHD3 domain (Fig. S2a†). Upon close inspection of the PHD3–H3K4me3 co-crystal structure, it was envisioned that stabilization of the H3 strand could be achieved through *i*, *i* + 2 tethering of the K4me3 ε-amino group with the proximal T6 sidechain, bridging across the surface-exposed aromatic ‘groove’ (Fig. 4A). Leveraging the highly symmetrical trimethylammonium functionality of the K4me3 sidechain as a robust synthetic handle, our synthetic plan involved quaternization of the ε-amino group of dimethyllysine with alkyl halides bearing sidechain functionalities to generate trimethyllysine-mimicking building blocks that are amenable for further diversification.

Tethering strategies were evaluated using minimization and conformational search tools in MacroModel based on the PHD3–H3K4me3 co-crystal structure (PDB 3GL6). We first introduced structural modifications to link the ε-ammonium group of the K4me3 sidechain and the β-carbon of the T6 residue. Three tethering strategies were considered to form lactam (series B), thioether (series C), or triazole-linked (series D) cyclopeptides with varying chain lengths (Fig. 4B–D). Substructures were subsequently minimized to yield a converged energy minimized state. Subsequently, a substructure conformational search was performed to assess the feasibility of proposed designs. Macrocyclization linkers were further triaged based on the functional group and alkyl chain lengths that accommodate the cation-π interaction between the derivatized K4me3 and two neighboring tryptophan residues (W1625 and W1635).

Next, we assessed the feasibility of the proposed strategies through optimization of alkylation conditions to functionalize the dimethyllysine sidechain on a solid support. Macrocyclization precursors were prepared with Fmoc-SPPS on Rink amide resin (see ESI†) by first loading Fmoc-Dap(Alloc)-OH (*N*_α_-Fmoc-*N*_β_-Alloc-l-2,3-diaminopropionic acid), Fmoc-Cys(S*t*Bu)-OH and Fmoc-Dap(N_3_)-OH (*N*_α_-Fmoc-*N*_β_-azido-l-2,3-diaminopropionic acid)/Fmoc-Dab(N_3_)-OH (*N*_α_-Fmoc-*N*_γ_-azido-l-2,4-diaminobutyric acid) for series B, C and D respectively, followed by elongation using standard amide coupling protocols (Fig. 4). Amide coupling of the Alloc-deprotected dimethyllysine precursor 16 with 3-bromopropionic acid proceeded smoothly using standard conditions (PyAOP, DIPEA), conveniently effecting a spontaneous intramolecular S_N_2 reaction to deliver lactam intermediate B-17 (Fig. 4, series B) in one step. Bis(bromomethyl)benzenes are commonly used reagents in cysteine–cysteine stapling and have been actively employed in the development of cell permeable peptide-based chemical tools.^31,32^ Inspired by existing literature methods underpinning the utility of this bifunctional linker, we envisaged that this approach could be extended to cysteine–dimethyllysine stapling, affording H3 cyclopeptides tethered through a thioether functionality (Fig. 4, series C). Thioether macrocycle intermediate C-18 was smoothly obtained upon subjection of the dimethyllysine precursor 19 to 1,2-bis(bromomethyl)benzene and DIPEA for one hour. Notably, further diversification of the thioether cyclopeptide intermediate C-18 proved feasible upon treatment with *meta*-chloroperoxybenzoic acid to afford sulfone intermediate C-20, which served as a valuable structural analogue for subsequent SAR analyses. Quaternization of dimethyllysine precursors 21 (*m* = 1) and 22 (*m* = 2) using propargyl bromide also proceeded smoothly and was followed by intramolecular copper(i)-catalyzed azide–alkyne cycloaddition to furnish triazole-containing cyclopeptide intermediates D-23 (*m* = 1) and D-24 (*m* = 2) (Fig. 4, series D, condition a). Interestingly, alkylation of dimethyllysine tripeptides 21 and 22 with 4-bromo-1-butyne was notably inefficient, where prolonged reaction times (*t* = 48 h) only led to trace amounts of the alkylated dimethyllysine product. This was eventually mitigated by reversing the order of the azide–alkyne cycloaddition and alkylation sequence, providing an alternative macrocyclization pathway which proceeded through an entropically favored intramolecular S_N_2 reaction (Fig. 4, series D, condition b) to efficiently access cyclopeptide intermediates D-25 (*m* = 1) and D-26 (*m* = 2). Cyclic tripeptide intermediates were subsequently elongated and subjected to acidic cleavage conditions to afford full-length, H3 cyclic peptides (see ESI†).

With the synthesized macrocyclic peptides in hand, competitive FP assays were performed to evaluate their binding affinity (Fig. 4E). Macrocyclic lactam B-17c led to a decrease in binding affinity by 10-fold compared to wild type peptide. To our delight, we identified two cyclic peptides containing a thioether or triazole linker (C-18c and D-23c respectively) that exhibited comparable activity to the wild type hexapeptide. Oxidation of the thioether moiety to the sulfone cyclopeptide C-20c resulted in a five-fold decrease in binding affinity, which could be due to the enhanced structural rigidity of the linker, disfavoring binding. Interestingly, amongst series D, triazole cyclopeptide D-23c bearing the smallest macrocycle size showed highest affinity for the PHD3 domain and increasing linker chain length led to a decrease in binding affinity (D-24c–26c). In contrast to the series C thioether C-18c and sulfone C-20c cyclopeptides, we hypothesized that the observed trend in ring size for series D is likely attributed to the less flexible, shortest triazole tether D-23c which pre-organizes the peptide into a favorable, binding-competent conformation. Remarkably, enhanced tryptic stability was observed for triazole cyclopeptide D-23c compared to linear wild type peptide 1. Wild type peptide 1 was highly susceptible to tryptic digestion (Fig. S2a†), whereas some degradation of D-23c was only noted after 48 h incubation (Fig. S2d†).

### Development of covalent cyclic peptide ligands

Covalent inhibitors offer broad utility in chemical biology applications, owing to their improvement in potency and prolonged duration of action.^33–36^ In particular, targeting of non-conserved nucleophilic residues proximal to the binding site can impart enhanced selectivity to inhibitors for structurally similar paralogs—a design feature highly desirable for chromatin reader domain ligands that bind to H3K4me3 marks. Despite these potential advantages, to the best of our knowledge, peptide-based covalent inhibitors for chromatin readers have not been previously described. Based on the close proximity of K1620 and K1622 to the Q5 sidechain (Fig. 1D), observed tolerance of Q5 towards mutagenesis in our alanine scan (Fig. 2A) and peptide SAR studies (Fig. 3), we surmised that replacement of this residue with a lysine-reactive covalent warhead could be tolerated.

**Fig. 5:**
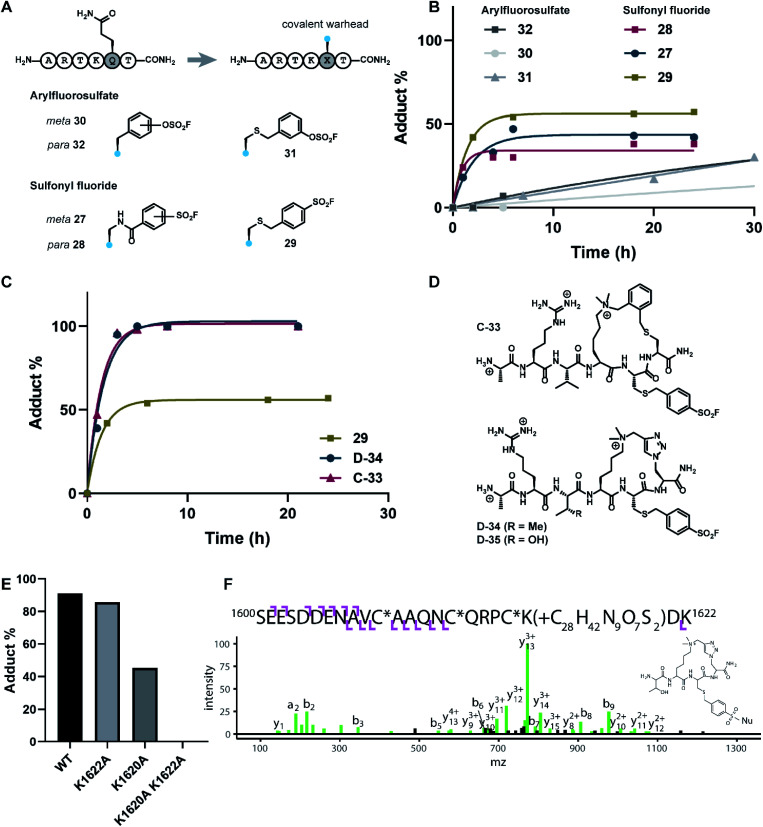
Design and biochemical evaluation of covalent peptides targeting the PHD3 domain. (A) H3K4me3 6mer peptides bearing covalent lysine-reactive warheads. (B) PHD3 (10 μM) was treated with peptides 27–32 at 50 μM, and the extent of labelling was monitored over time by intact protein LCMS. (C) Comparison of cross-linking efficiency between linear (29) and cyclic (C-33 and D-34) peptides. (D) Structures of covalent cyclic peptides C-33, D-34, and D-35. (E) Comparison of peptide-PHD3 adduct formation between WT and mutant PHD3 after 5 h incubation time. Triazole probe D-35 was used for this experiment. (F) HCD product ion spectrum of the triazole probe (D-35)-labeled PHD3 domain following tryptic digestion, corresponding to precursor ion at *m*/*z* = 678.879^5+^. C* indicates carbamidomethylated Cys. The structure of the probe adduct following digestion is shown and the spectrum indicates K1620 as the site of modification.

Sulfonyl fluorides and aryl fluorosulfates have emerged as privileged functionalities in chemical probe development, owing to their context-specific reactivity influenced by residues near the binding region of the electrophilic warhead.^37,38^ In contrast to cysteine-selective electrophiles such as maleimides, sulfonyl fluorides and arylfluorosulfates react with nucleophilic residues including tyrosine, lysine, threonine, and serine depending on the nature of the binding site.^37,39–41^ In recent years, lysine-targeted sulfonyl fluoride probes have been employed in innovative broad-spectrum kinase profiling systems.^42^ The utility of sulfur(vi) halides as latent, activatable electrophiles has been elegantly showcased in an ‘Inverse Drug Discovery’ platform, leveraging arylfluorosulfate-containing small molecules to screen the cellular proteome and identifying distinct groups of proteins that can facilitate covalent modification.^43^ Inspired by previous work on lysine-reactive chemical probe development using sulfonyl fluoride and arylfluorosulfate chemistries,^37–41,43–45^ our synthetic efforts centered on the investigation of mutant peptides bearing arylsulfonyl fluoride and arylfluorosulfate covalent warheads. To probe the nucleophilicity of K1620 and K1622, we prepared a library of linear peptides where the Q5 residue was substituted with a lysine-reactive electrophile (Fig. 5A). Sulfonyl fluoride-containing peptides were synthesized using Fmoc-SPPS, derivatizing the Q5 position with *S*-(*t*Bu)-protected Cys or Alloc-protected Dap amino acid building blocks. Orthogonal deprotection of the Alloc and *S*-(*t*Bu) groups proceeded smoothly upon treatment with dithiothreitol/DIPEA or Pd(PPh_3_)_4_/PhSiH_3_, respectively (see ESI†). Incorporation of the benzylsulfonyl fluoride functionality could be readily achieved on solid support, through an S_N_2 reaction between the deprotected Q5C hexapeptide and 4-bromobenzylsulfonyl fluoride. Alternatively, fluorosulfonyl benzoic acids could be introduced onto Dap sidechains using standard amide coupling conditions to deliver modified peptides bearing fluorosulfonyl benzamide functionalities. The synthesis of arylfluorosulfate peptides followed a slightly modified published procedure,^46^ employing [4-(acetylamino)phenyl]imidodisulfuryl difluoride as a bench-stable reagent for the selective installation of –SO_2_F groups on phenolic substrates. Synthesized covalent peptides were evaluated for their ability to disrupt the PHD3–H3K4me3 complex using the competitive FP assay, where efficient displacement of the wild type peptide with covalent peptide analogues was observed, with inhibition constants ranging from 0.11–0.37 μM (Fig. S4†).

The extent of covalent modification was assessed using intact protein mass spectrometry. Briefly, covalent peptides were incubated with recombinant PHD3 protein in buffer (50 mM HEPES pH 7.5, 50 mM KCl) at 37 °C, and reaction aliquots were monitored over time by LCMS analysis. We found that arylsulfonyl fluoride peptides (27–29) exhibited faster covalent modification (6–8 hours) compared to arylfluorosulfate peptides (30–32), although the extent of modification reached a plateau at 30–60%, presumably due to competitive hydrolysis of the sulfonyl fluoride functionality leading to deactivation of the covalent warhead (Fig. 5B). Contrastingly, covalent addition of arylfluorosulfate peptides were considerably slower, where the most reactive arylfluorosulfate peptide 32 reached ∼30% occupancy after 30 hours incubation time. We reasoned that steady increase in PHD3-arylfluorosulfate adduct over a longer timeframe was due to enhanced hydrolytic stability of the arylfluorosulfate peptides at physiological pH. Amongst the arylsulfonyl fluoride peptides tested, 29 was the most efficient at covalent modification of the PHD3 domain, likely attributed to the increased flexibility of the thioether linker and greater stability of the benzylsulfonyl fluoride moiety in buffer.

The best-performing benzylsulfonyl fluoride warhead was merged with the most potent cyclic peptide scaffolds identified during our SAR studies (Fig. 3 and 4) to generate covalent cyclopeptides C-33, D-34 and D-35 (Fig. 5D). Synthesis of macrocyclic peptides C-33, D-34 and D-35 bearing the cysteine-conjugated benzylsulfonyl fluoride was achieved by Fmoc-SPPS, relying on orthogonal protecting group strategies to ensure the efficient installation of both the macrocyclic scaffold and covalent warhead on solid support (see ESI†). For example, 4-methoxytrityl and *S*-(*t*Bu) groups were elected to mask C5 and C6 in the thioether cyclopeptide (C-33) synthesis, allowing for controlled and selective derivatization of adjacent cysteine sidechains during the Fmoc-SPPS process. Covalent cyclopeptides C-33 and D-34 exhibited comparable binding affinity to the wildtype linear peptide (ESI Fig. S4†). Notably, both covalent cyclopeptides quantitatively labeled the recombinant PHD3 protein after six hours incubation time, significantly outperforming the linear benzylsulfonyl fluoride peptide 29 (Fig. 5C).

### Lys1620 is the primary site of covalent modification

To identify the site of modification within the PHD3 domain, we performed site-directed mutagenesis to replace K1620 and K1622 with alanine and assessed the effect on covalent conjugate formation. Mutant PHD3 domains retained the ability to bind the histone ligand (Fig. S3†). Intact protein MS experiments with single point mutants of PHD3—K1620A and K1622A, revealed K1620 as the major site for covalent modification by triazole cyclopeptide D-35, although we also observed functionalization at the K1622 position (Fig. 5E). The site of reaction was also assessed by tandem mass spectrometry of both digested and intact PHD3. Tryptic digests of D-35-labeled PHD3 produced MS2 spectra with unambiguous site localization of the proteolyzed D-35 fragment at K1620 (Fig. 5F and S5a†) as well as at K1622 (Fig. S5b†) and at the N-terminus of the PHD3 domain construct (data not shown). Electron transfer with supplemental collision induced dissociation (ETciD) analysis of intact, covalently modified PHD3 displayed c- and z-product ions that were diagnostic of both K1620 and K1622 modification (data not shown). No evidence of N-terminal modification was observed in the intact experiments suggesting that this adduct may be an artifact of digestion. While it is impossible to conclude which site is preferentially labeled without the availability of peptide standards, spectral counts and ion intensities of both the precursor ions (digest sample) and product ions (intact sample) are consistent with a preference for modification at K1620 over K1622. Remarkably, the PHD3 double mutant K1620A/K1622A was completely resistant to covalent addition even when treated with five-fold excess of triazole cyclopeptide D-35, demonstrating the selectivity for the two surface-exposed lysine residues (Fig. 5E). This finding indicates that covalent adduct formation of triazole cyclopeptide D-35 with WT and K1622A PHD3 was enabled only by proximity labeling within the protein–peptide complex, as opposed to non-specific reactivity, which would result in modification of other nucleophilic residues in the PHD3 domain.

**Fig. 6:**
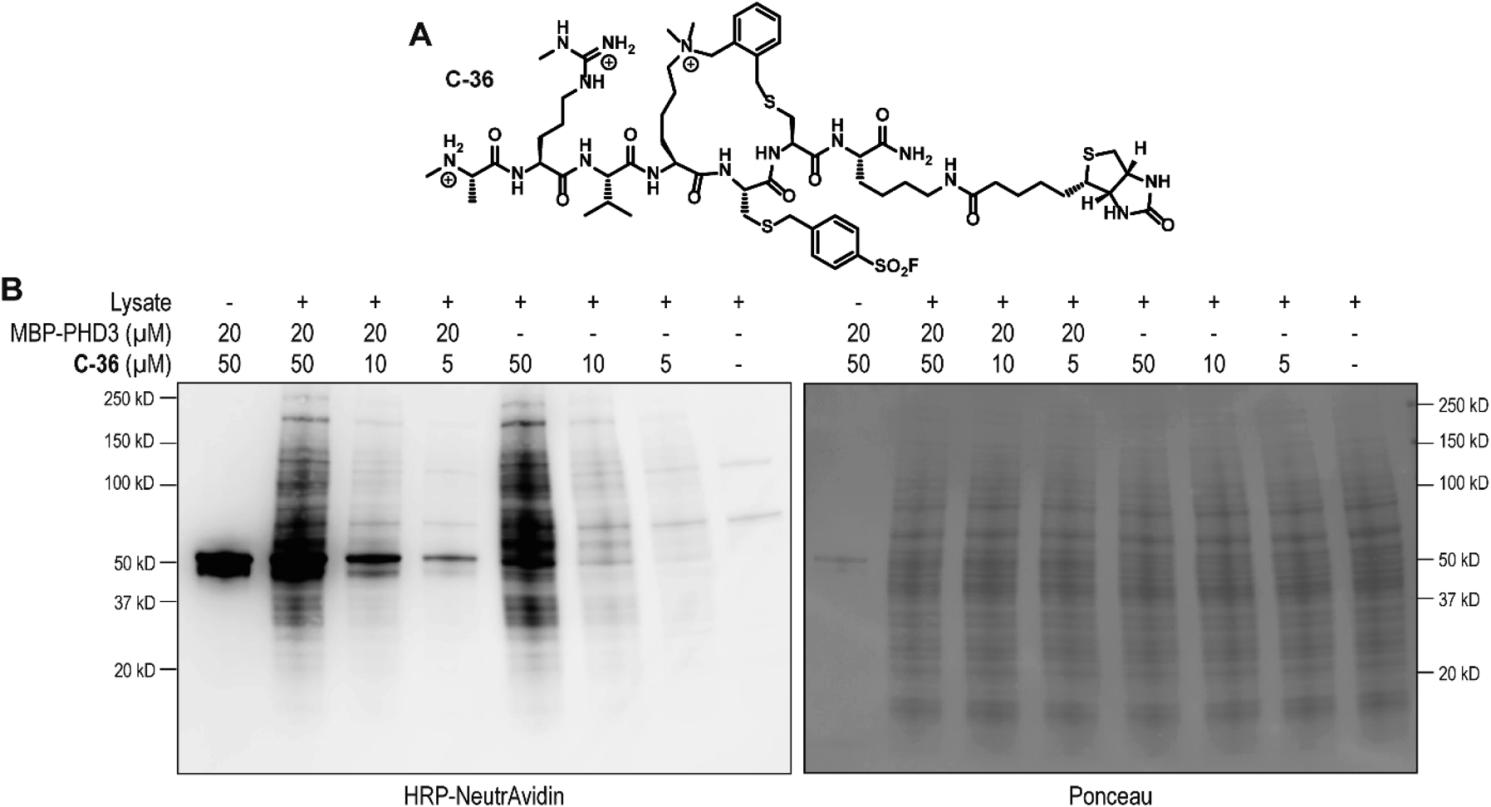
Chemical structure of cyclic covalent biotinylated probe C-36 and western blotting analysis of HEK293T cell lysate labelling. (A) Chemical structure of thioether covalent biotinylated probe C-36. (B) SDS-PAGE/western blot analysis of HEK293T cell lysate and recombinant His_6_-MBP-PHD3 labelling (4.5 h at RT) using biotinylated thioether sulfonyl fluoride probe C-36.

Finally, we investigated the ability of thioether sulfonyl fluoride probe C-33 to covalently modify the PHD3 domain in the context of HEK293T cell lysates. A biotinylated analogue, thioether cyclopeptide C-36 was prepared for cell lysate covalent labelling experiments (Fig. 6A). Importantly, addition of the biotin functionality did not impair binding of the peptide to the PHD3 domain as indicated by comparable inhibition constants of C-33 and C-36 (Fig. S4†). To assess the ability of biotinylated probe C-36 to effect the pulldown, lysates were supplemented with recombinant His_6_-MBP-PHD3 protein (20 μM). Spiked HEK293T lysates were treated with varying concentrations of biotinylated thioether probe C-36 (*t* = 4.5 h at RT), and biotin-labeled proteins were analyzed by western blot (HRP-conjugated NeutrAvidin antibody). Strong labelling of His_6_-MBP-PHD3 was observed, demonstrating the ability of cyclopeptide C-36 to covalently interact with the PHD3 domain within the complex proteome (Fig. 6B). While useful in targeting protein–protein interfaces, a common drawback of peptide modalities includes limited cell permeability. Structural optimizations^47,48^ as well as conjugation of cell-penetrating peptide tags^49–51^ should enable use of these peptide tools in future cellular experiments.

## Conclusions

The last few years have been marked by the resurgence of covalent peptide-based chemical probes, highlighted by their widespread applications in the development of novel pharmacological tools and viral protease inhibitors.^45,52–56^ These modalities complement existing small molecule approaches, particularly for targets that lack well-defined binding pockets. We herein describe the first covalent peptide-based probe for the PHD3 domain of histone demethylase KDM5A. Systematic SAR studies of the N-terminal H3K4me3 ligand for the PHD3 domain identified a hexapeptide sequence which engaged the binding site with sub-micromolar affinity. Aided by computational modelling, we discovered macrocyclic H3K4me3 peptide scaffolds with comparable binding affinity and improved proteolytic stability. The structurally unique, surface-exposed aromatic ‘groove’ of the PHD3 domain inherently enabled a macrocyclization strategy which involved sidechain-to-sidechain linking of Lys4 and Thr6. Notably, the highly symmetrical tetraalkylammonium moiety of the K4me3 sidechain was judiciously exploited in the development of macrocyclization strategies which avoided synthetic bottlenecks associated with the introduction of additional stereocenters. The presence of surface-exposed lysine residues adjacent to the histone binding site enabled the rational design of covalent cyclopeptide analogues. Our studies set the first example of a proximity-reactive cyclic peptide which targets a chromatin reader domain. These covalent cyclopeptide probes enable capturing of the PHD3 domain in cell lysates, laying the groundwork for developing valuable occupancy probes for future PHD3 ligand discovery programs. Given the paucity of well-characterized ligands for reader domains of chromatin methylation, we also anticipate that the compounds described herein will be instrumental for continued efforts towards targeting this challenging family of epigenetic proteins.

## Data availability

Experimenal procedures and characterization data have all been included into the ESI† file.

## Author contributions

MYZ contributed to conceptualization, data curation, formal analysis, investigation, methodology, and writing – original draft; HY contributed to investigation, methodology, writing – original draft; GO contributed to investigation, methodology, writing – review & editing; MJT contributed to data curation, formal analysis, investigation, writing – original draft; NP contributed to investigation and methodology; ALB contributed to resources and supervision; WFD contributed to methodology, resources, supervision, writing – review & editing; DGF contributed to conceptualization, funding acquisition, supervision, writing – review & editing.

## Conflicts of interest

There are no conflicts to declare.

## Supplementary Material

SC-013-D2SC00555G-s001
