# Supplementary material for: Covalent labeling of a chromatin reader domain using proximity-reactive cyclic peptides
Source: Chem Sci. 2022 May 12;13(22):6599–609. doi: 10.1039/d2sc00555g (PMC9172573; doi:10.1039/d2sc00555g)
Supplement: SC-013-D2SC00555G-s001 [file SC-013-D2SC00555G-s001.pdf]

Electronic Supplementary Material (ESI) for Chemical Science.
This journal is © The Royal Society of Chemistry 2022

Electronic Supplementary Information

Covalent labeling of a chromatin reader domain using proximity-reactive cyclic peptides

Meng Yao Zhang,[a] Hyunjun Yang,[b] Gloria Ortiz,[a] Michael J. Trnka,[b] Nektaria Petronikolou,[a] Alma L. Burlingame,[b] William F. DeGrado,[b] and Danica Galonić Fujimori[a,b,c]*

[a] Department of Cellular Molecular Pharmacology, University of California San Francisco, San Francisco, CA 94158, USA.

[b] Department of Pharmaceutical Chemistry, University of California San Francisco, San Francisco, CA 94158, USA.

[c] Quantitative Bioscience Institute, University of California San Francisco, San Francisco, CA 94158, USA.

* E-mail: danica.fujimori@ucsf.edu

Table of Contents

Supplementary Figure 1 3
Supplementary Figure 2 4
Supplementary Figure 3 8
Supplementary Figure 4 9
Supplementary Figure 5 10
Reagents and Materials 11
General Experimental Procedures 11
Molecular Modelling 11
Synthetic Procedures and Peptide Characterization Data 13
General Workflow for Fmoc-Solid Phase Peptide Synthesis (Fmoc-SPPS) 13
Macrocyclization Synthetic Procedures 35
Covalent Warhead Synthetic Procedures 53
Synthesis of covalent cyclopeptides 69
Expression and purification of His_6-MBP-PHD3, WT and mutant PHD3 80
Fluorescence polarization assays 81
In vitro PHD3 labelling reactions 82
Cell lysate labelling experiments 82
References 83

Supplementary Figure 1

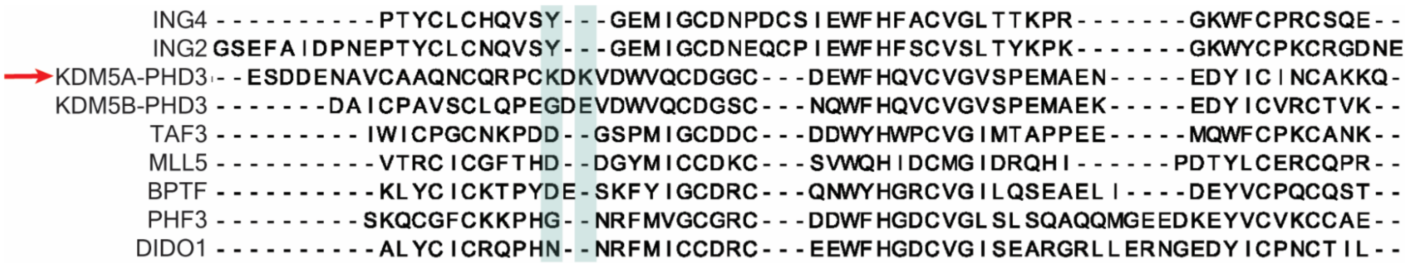

Fig. S1: Sequence alignment of KDM5A-PHD3 with other H3K4me3-recognizing PHD domains.

Supplementary Figure 2

Trypsin stability assay: Peptide and trypsin (1:1 w/w) were mixed in 1x PBS buffer and the resulting mixture was incubated at RT. Proteolysis reactions at a given time point was quenched by adding HCl. The proteolysis process was monitored by subjecting the quenched mixture onto reverse-phase HPLC column monitoring at 214 nm [H_2O (A):MeCN (B); 5% to 100% solvent B over 23 min].

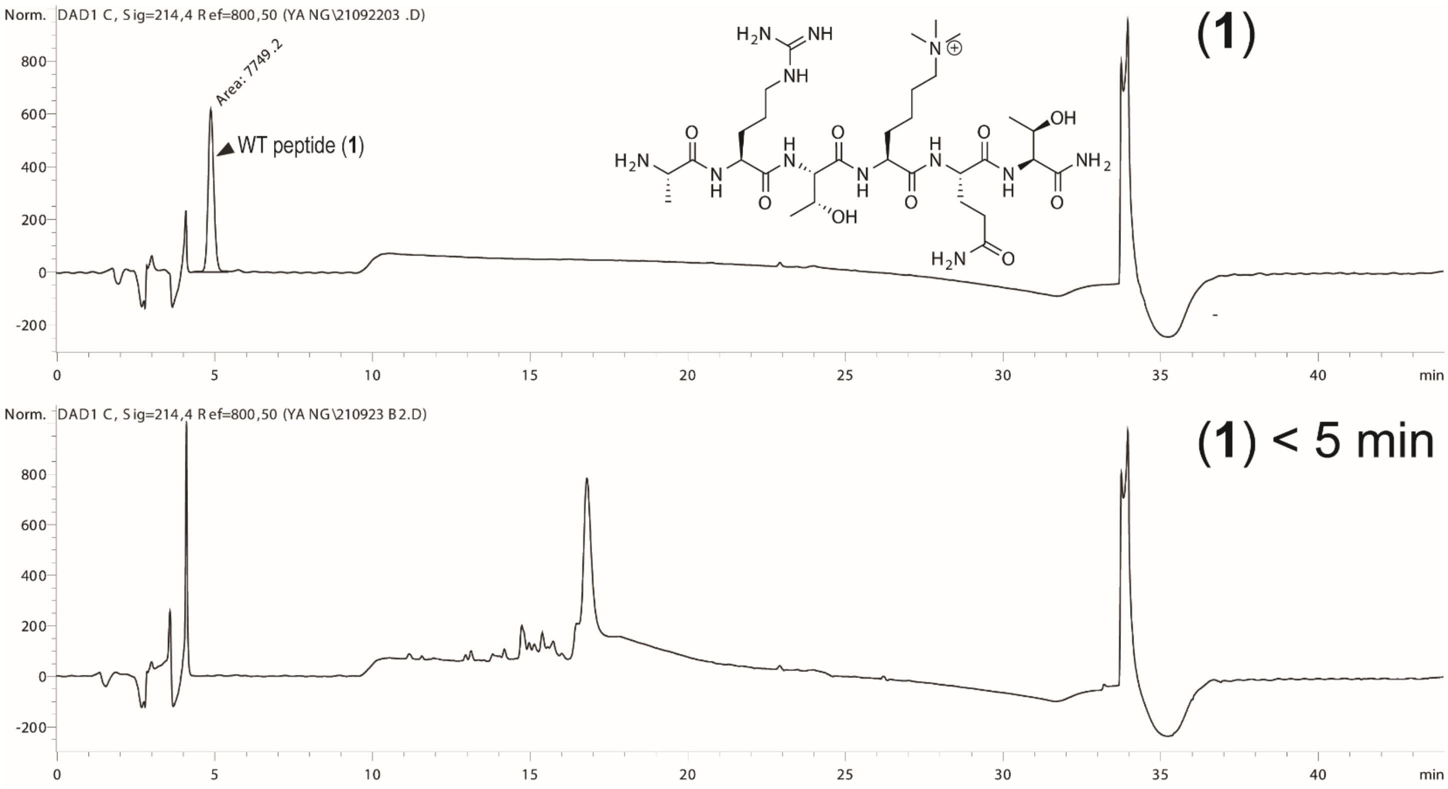

Fig. S2a: Chromatograms of WT H3K4me3 hexapeptide (**1**) before and after incubation with trypsin (*t* = < 5 min).

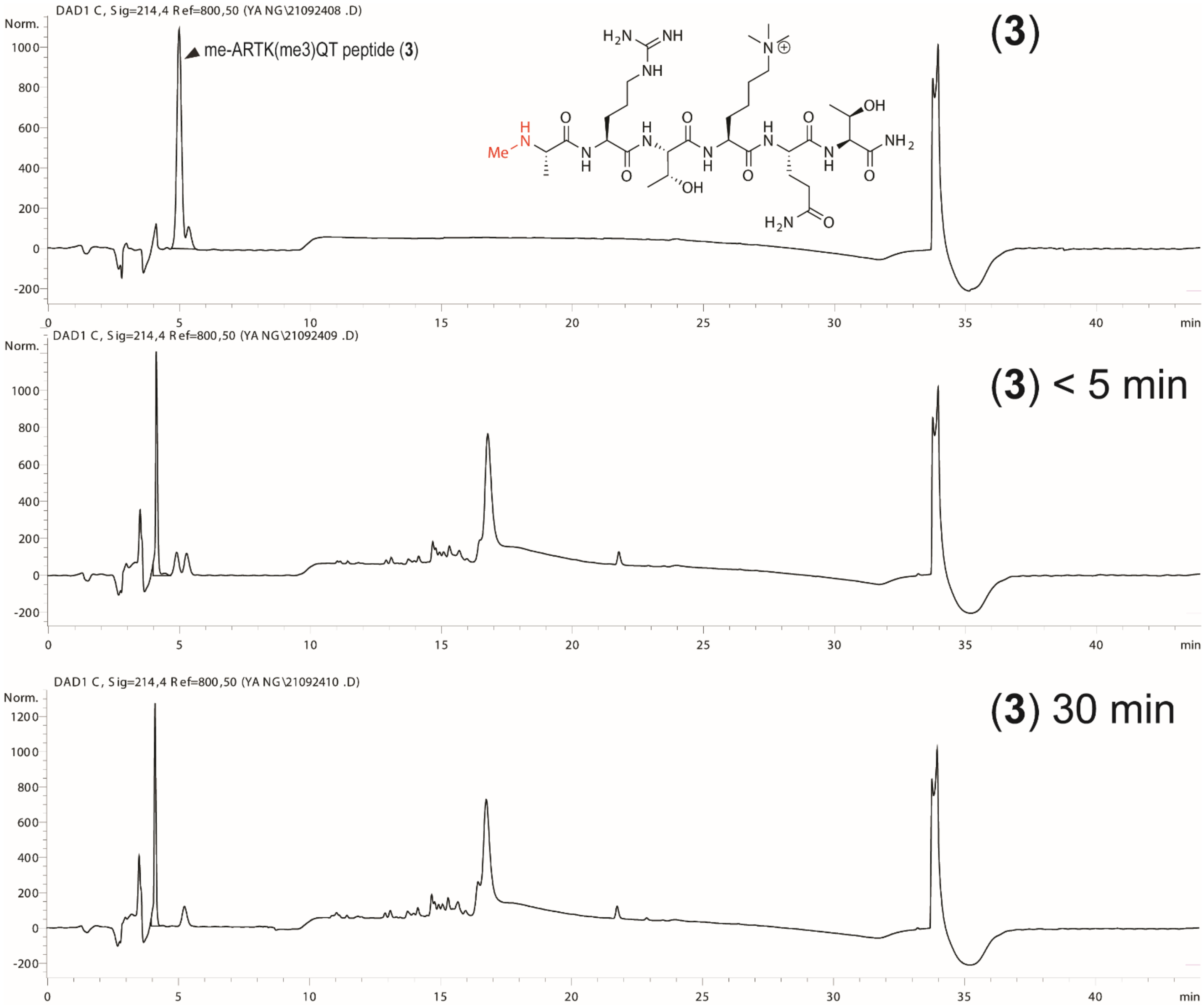

Fig. S2b: Chromatograms of me-ARTK(me3)QT hexapeptide (**3**) before and after incubation with trypsin (*t* = < 5 min, 30 min).

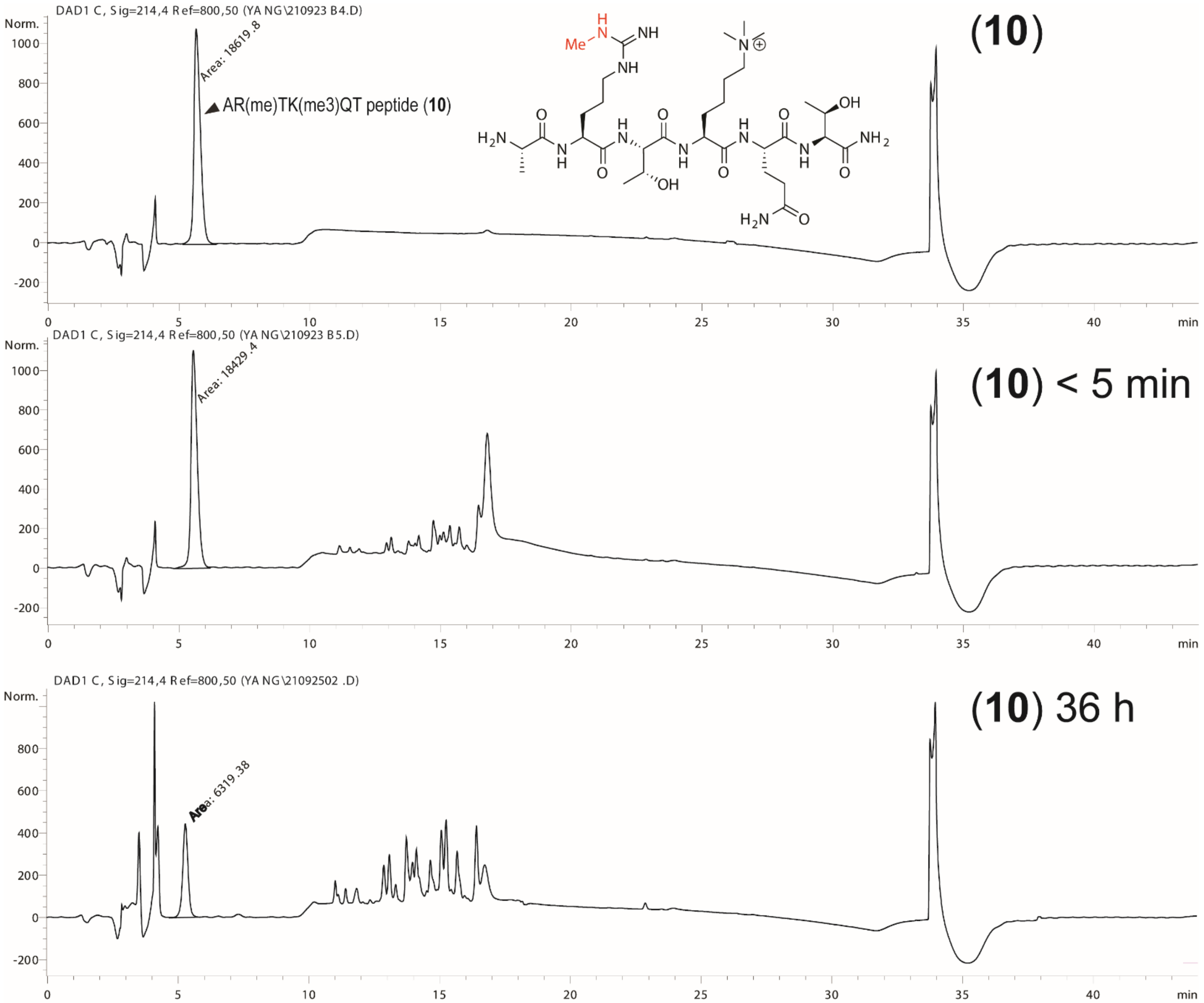

Fig. S2c: Chromatograms of AR(me)TK(me3)QT hexapeptide (**10**) before and after incubation with trypsin (*t* = < 5 min, 36 h).

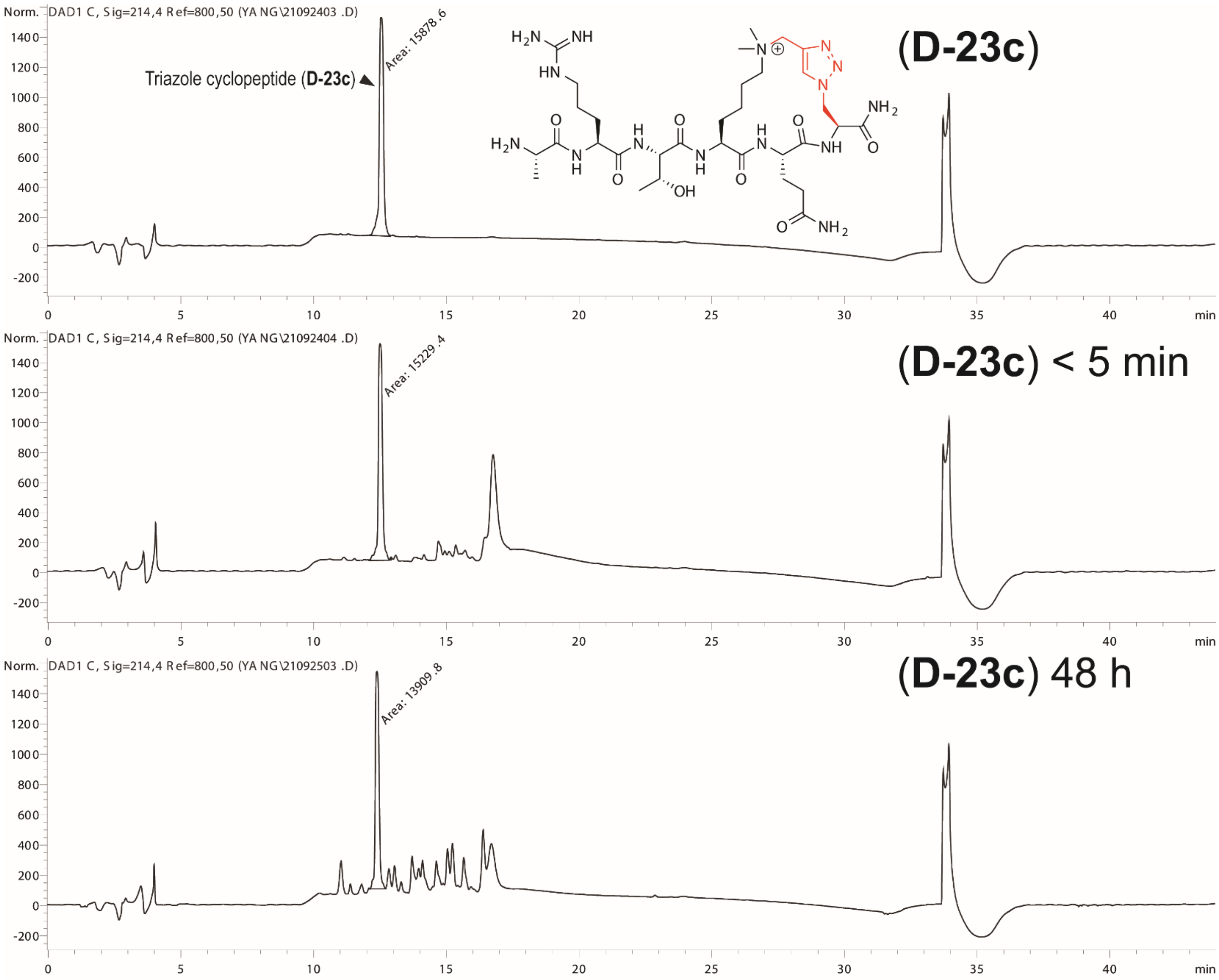

Fig. S2d: Chromatograms of triazole cyclopeptide (**D-23c**) before and after incubation with trypsin (t = < 5 min, 48 h).

Supplementary Figure 3

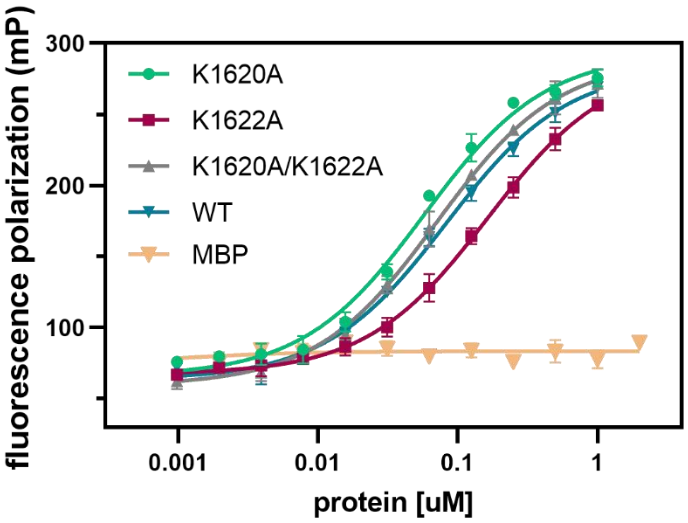

Protein	K_d (μM ± SE)
K1620A	0.058 ± 0.0071
K1622A	0.16 ± 0.0054
K1620A K1622A	0.071 ± 0.0042
WT	0.083 ± 0.0055
His_6-MBP	NB

Fig. S3: Fluorescence polarization binding curves (top) and K_d values of WT and mutant His_6-MBP-PHD3 (bottom). Fluorescence polarization binding assays used C-terminally fluorescently labeled H3K4me3 10mer peptide as the tracer, and K_d values are presented as average ± standard error of three replicates.

Supplementary Figure 4

Peptide	PHD domain	Compound number	K_i (µM ± SE)
Linear (arylfluorosulfate)	PHD3	**30**	0.22 ± 0.039
Linear (arylfluorosulfate)	PHD3	**31**	0.17 ± 0.020
Linear (arylfluorosulfate)	PHD3	**32**	0.37 ± 0.077
Linear (SF)	PHD3	**27**	0.13 ± 0.023
Linear (SF)	PHD3	**28**	0.11 ± 0.020
Linear (SF)	PHD3	**29**	0.22 ± 0.048
Cyclic (SF)	PHD3	Triazole **D-34**	0.51 ± 0.097
Cyclic (SF)	PHD3	Triazole **D-35**	0.38 ± 0.065
Cyclic (SF)	PHD3	Thioether **C-33**	0.50 ± 0.068
Cyclic (SF) biotinylated	PHD3	Thioether **C-36**	0.21 ± 0.040
Cyclic (SF)	PHD1*	Triazole **D-35**	188.8 ± 42.05

Fig. S4: Competitive fluorescence polarization binding assay data for covalent linear and cyclic peptides. Inhibition constants are presented as average ± standard error of three replicates.

* The inhibition constant for triazole **D-35** binding to PHD1 was obtained using recombinant GST-PHD1 and fluorescently labelled H3K4me0 10-mer peptide, as previously described.[1]

Supplementary Figure 5

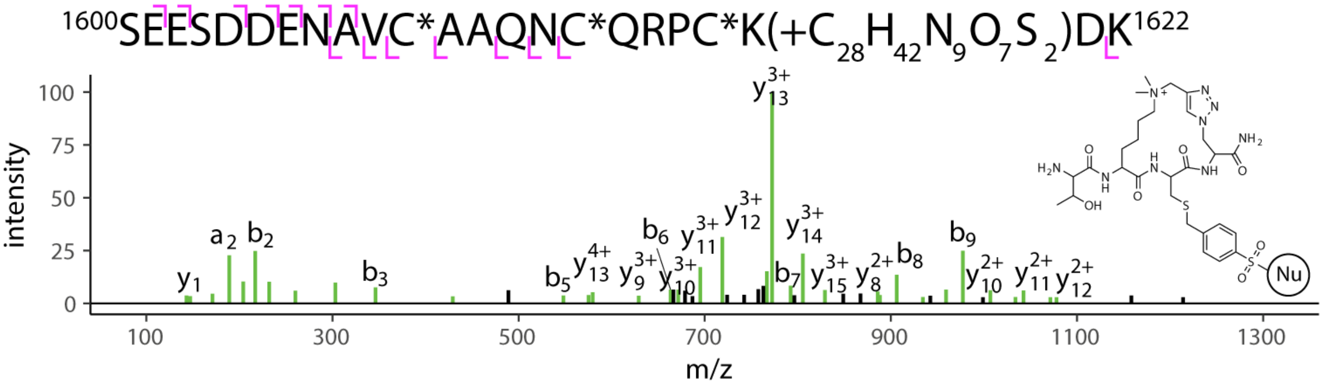

$b-H_2O$	---	199.0713	328.1139	415.146	530.1729	645.1998	774.2424	888.2854	959.3225	1058.3909	1218.4215	1289.4587
$b-H_2O^{2+}$	---	---	---	---	---	---	---	---	---	---	---	---
b	---	217.0819	346.1245	433.1565	548.1835	663.2104	792.253	906.2959	977.333	1076.4015	1236.4321	1307.4692
b^{2+}	---	---	---	---	---	---	---	---	---	---	---	---
	1	2	3	4	5	6	7	8	9	10	11	12
-	S	E	E	S	D	D	E	N	A	V	C*	A
	23	22	21	20	19	18	17	16	15	14	13	12
y	---	3303.334	3174.2914	3045.2488	2958.2168	2843.1899	2728.1629	2599.1203	2485.0774	2414.0403	2314.9719	2154.9412
y^{2+}	---	1652.1707	1587.6494	1523.1281	1479.612	1422.0986	1364.5851	1300.0638	1243.0423	1207.5238	1157.9896	1077.9743
y^{3+}	---	1101.7829	1058.7687	1015.7545	986.7438	948.4015	910.0592	867.045	829.0307	805.3516	772.3288	718.9853
$y-NH_3$	---	3286.3075	3157.2649	3028.2223	2941.1903	2826.1633	2711.1364	2582.0938	2468.0509	2397.0137	2297.9453	2137.9147
$y-NH_3^{2+}$	---	1643.6574	1579.1361	1514.6148	1471.0988	1413.5853	1356.0718	1291.5505	1234.5291	1199.0105	1149.4763	1069.461
$y-NH_3^{3+}$	---	1096.1073	1053.0931	1010.0789	981.0683	942.726	904.3836	861.3694	823.3551	799.6761	766.6533	713.3097

$b-H_2O$	1360.4958	1488.5543	1602.5973	1762.6279	1890.6865	2046.7876	2143.8404	2303.871	3111.223	3226.25	---
$b-H_2O^{2+}$	---	---	---	---	---	1023.8974	1072.4238	1152.4391	1556.1151	1613.6286	---
b	1378.5063	1506.5649	1620.6078	1780.6385	1908.6971	2064.7982	2161.8509	2321.8816	3129.2336	3244.2605	---
b^{2+}	---	---	---	---	---	1032.9027	1081.4291	1161.4444	1565.1204	1622.6339	---
	13	14	15	16	17	18	19	20	21	22	23
-	A	Q	N	C*	Q	R	P	C*	K(+MOD)	D	K
	11	10	9	8	7	6	5	4	3	2	1
y	2083.9041	2012.867	1884.8084	1770.7655	1610.7348	1482.6763	1326.5752	1229.5224	1069.4917	262.1397	147.1128
y^{2+}	1042.4557	1006.9371	942.9078	885.8864	805.8711	741.8418	663.7912	615.2648	535.2495	131.5735	74.06
y^{3+}	695.3062	671.6272	628.941	590.9267	537.5831	494.8969	442.8632	410.5123	357.1688	---	---
$y-NH_3$	2066.8776	1995.8405	1867.7819	1753.7389	1593.7083	1465.6497	1309.5486	1212.4958	1052.4652	245.1132	130.0863
$y-NH_3^{2+}$	1033.9424	998.4239	934.3946	877.3731	797.3578	733.3285	655.2779	606.7516	526.7362	123.0602	65.5468
$y-NH_3^{3+}$	689.6307	665.9517	623.2655	585.2512	531.9076	489.2214	437.1877	404.8368	351.4933	---	---

Fig. S5a: HCD product ion spectrum of 678.6790^{5+} corresponding to covalent modification at K1620 of PHD3.

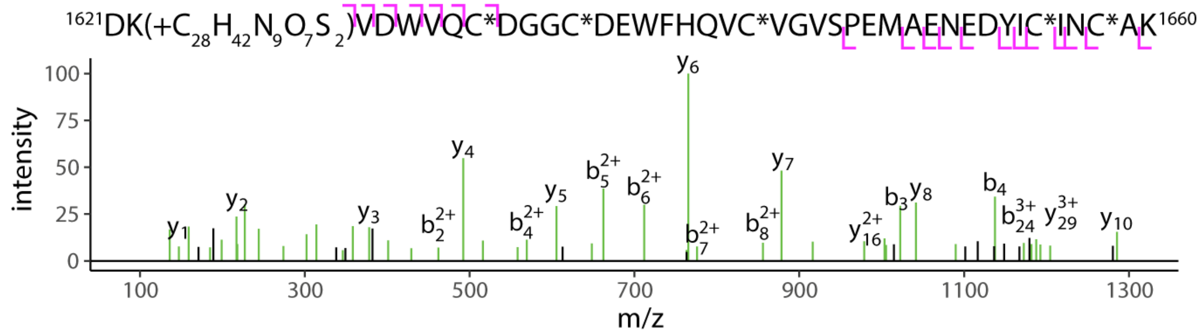

b	---	923.3862	1022.4546	1137.4816	1323.5609	1422.6293	1550.6879	1710.7185	1825.7455	1882.7669	1939.7884	2099.819	2214.846	2343.8886	2529.9679	2677.0363	2814.0952	2942.1538	3041.2222	3201.2529
b^{2+}	---	462.1967	511.731	569.2444	662.2841	711.8183	775.8476	855.8629	913.3764	941.8871	970.3978	1050.4132	1107.9266	1172.4479	1265.4876	1339.0218	1407.5512	1471.5805	1521.1147	1601.1301
	1	2	3	4	5	6	7	8	9	10	11	12	13	14	15	16	17	18	19	20
-	D	K(+MOD)	V	D	W	V	Q	C*	D	G	G	C*	D	E	W	F	H	Q	V	C*
	40	39	38	37	36	35	34	33	32	31	30	29	28	27	26	25	24	23	22	21
y	---	5384.2226	4576.8706	4477.8022	4362.7752	4176.6959	4077.6275	3949.5689	3789.5383	3674.5113	3617.4899	3560.4684	3400.4377	3285.4108	3156.3682	2970.2889	2823.2205	2686.1616	2558.103	2459.0346
y^{2+}	---	2692.6149	2288.9389	2239.4047	2181.8912	2088.8516	2039.3174	1975.2881	1895.2728	1837.7593	1809.2486	1780.7378	1700.7225	1643.209	1578.6877	1485.6481	1412.1139	1343.5844	1279.5551	1230.0209

b	3300.3213	3357.3427	3456.4112	3543.4432	3640.4959	3769.5385	3900.579	3971.6161	4100.6587	4214.7017	4343.7443	4458.7712	4621.8345	4734.9186	4894.9492	5008.0333	5122.0762	5282.1069	5353.144	---
b^{2+}	1650.6643	1679.175	1728.7092	1772.2252	1820.7516	1885.2729	1950.7931	1986.3117	2050.833	2107.8545	2172.3758	2229.8892	2311.4209	2367.9629	2447.9783	2504.5203	2561.5418	2641.5571	2677.0756	---
	21	22	23	24	25	26	27	28	29	30	31	32	33	34	35	36	37	38	39	40
-	V	G	V	S	P	E	M	A	E	N	E	D	Y	I	C*	I	N	C*	A	K
	20	19	18	17	16	15	14	13	12	11	10	9	8	7	6	5	4	3	2	1
y	2299.0039	2199.9355	2142.9141	2043.8456	1956.8136	1859.7608	1730.7183	1599.6778	1528.6407	1399.5981	1285.5551	1156.5125	1041.4856	878.4223	765.3382	605.3076	492.2235	378.1806	218.1499	147.1128
y^{2+}	1150.0056	1100.4714	1071.9607	1022.4265	978.9104	930.3841	865.8628	800.3425	764.824	700.3027	643.2812	578.7599	521.2464	439.7148	383.1727	303.1574	246.6154	189.5939	109.5786	74.06

Fig. S5b: HCD product ion spectrum of 1100.6557^{5+} corresponding to covalent modification at K1622 of PHD3.

Reagents and Materials

Fmoc-solid phase peptide synthesis was performed using fritted plastic syringes from TORVIQ. Rink amide resin (0.54 mmolg^{-1},100-200 mesh) was manufactured by Novabiochem® and purchased from Sigma-Aldrich. Fmoc-protected amino acids and reagents used for peptide synthesis were purchased from AK Scientific, Iris Biotech, AstaTech, Combi-blocks, Chem-Impex and Sigma-Aldrich, and were used without further purification. Peptides used for the alanine scan, C-terminal truncation scan, Ac-ARTK(me3)QT-NH_2, SRTK(me3)QT-NH_2, PRTK(me3)QT-NH_2, AibRTK(me3)QT-NH_2 were purchased from Genscript.

General Experimental Procedures

Preparative HPLC was performed on a Waters Prep 150Q LC system, with a Waters 2998 Photodiode Array detector, and this system was operated using ChromScope software. All separations used linear gradients of water containing 0.1% trifluoroacetic acid and acetonitrile, at a flow rate of 10 mL/min (XSelect® Peptide CSH™ C18, OBD™ Prep Column, 130Å, 5 µM, 19 mm × 250 mm, 1/pkg). Analytical HPLC was performed on a Varian ProStar 210 Solvent Delivery Module, with a Varian ProStar 335 Photodiode Array Detector, and this system was operated using Galaxy Chromatography Data System. All separations used linear gradients of water containing 0.1% trifluoroacetic acid (solvent A) and acetonitrile (solvent B), at a flow rate of 1 mL/min (Phenomenex Luna® 10 µm C18(2) 100Å, LC column 250 × 4.6 mm, Ea).

Analytical HPLC methods used for peptide characterization:

Method 1: 3% B for 1 minute, 3% to 30% B over 10 minutes, then 30% to 100% B over 1 minute (λ = 220 nm).

Method 2: 10% B for 1 minute, 10% to 95% B over 12 minutes and hold at 95% for 3 minutes (λ = 220 nm).

Method 3: 10% B for 1 minute, 10% to 95% B over 12 minutes and hold at 95% for 6 minutes (λ = 220 nm).

Analytical UPLC was performed on a Waters Acquity system fitted with a TUV detector. Separations for small molecules and peptides used linear gradients of water containing 0.1% formic acid (solvent A1) and acetonitrile containing 0.1% formic acid (solvent B1), at a constant flow rate (0.2 mL/min, BEH, C18, 1.7 µm). Separations for proteins used linear gradients of water containing 0.1% formic acid and 0.05% trifluoroacetic acid (solvent A2), and acetonitrile containing 0.1% formic acid (solvent B2), at a constant flow rate (0.2 mL/min, protein BEH C4, 300Å, 1.7 µm).

Molecular Modelling

Design feasibility of macrocyclic peptides were assessed using MacroModel. The X-ray crystallographic structure of PHD3-H3K4me3 (PDB 3GL6) was prepared as the initial starting structure. Non-coordinating water molecules found beyond 5 Å from heteroatoms were removed. Macrocyclic peptides were prepared by mutating K4me3 and T6 with a linker which contained amide, thioether, and triazole moieties respectively. The resulting structures were minimized using MMFFs and GB/SA water solvation whilst keeping the PHD3 and amide backbone of H3K4me3 frozen. The minimized model was subsequently searched for energy conformers using the conformational search tool using the MMFFs force field and GV/SA water solvation whilst keeping the PHD3 and

amide backbone of H3K4me3 frozen. The resulting conformers within 4 $kJmol^{-1}$ from the lowest-energy conformer were overlayed to assess the rigidity and design feasibility.

Synthetic Procedures and Peptide Characterization Data

General Workflow for Fmoc-Solid Phase Peptide Synthesis (Fmoc-SPPS)

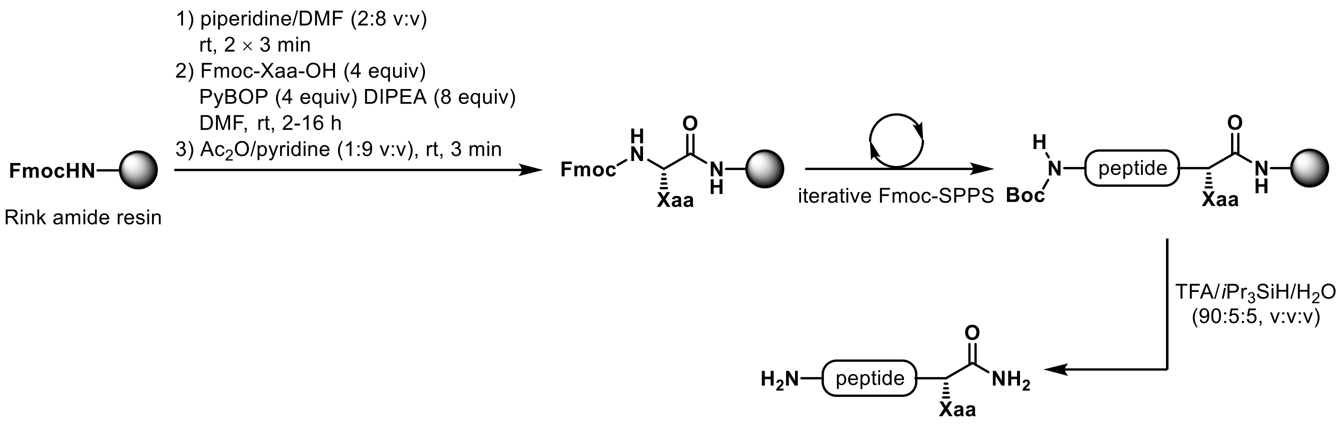

Loading Rink amide resin

In a fritted plastic syringe, Rink amide resin (1.0 equiv, substitution 0.54 mmolg^{-1}) was swollen in DMF for 15 min, then washed with DCM (5 × 3 mL) and DMF (5 × 3 mL). The resin-bound Fmoc group was removed by adding a solution of piperidine/DMF (2:8 v:v). The liquid was discarded after three minutes and the deprotection reaction was repeated once more. The resin was washed with DMF (5 × 3 mL), DCM (5 × 3 mL) and DMF (5 × 3 mL).

A solution of Fmoc-Xaa-OH (4 equiv), PyBOP (4 equiv) and DIPEA (8 equiv) in DMF was added to the resin and shaken at room temperature for 2-16 hours, at which time the resin was washed with DMF (5 × 3 mL), DCM (5 × 3 mL) and DMF (5 × 3 mL).

A solution of Ac_2O/pyridine (1:9 v:v) was added to the resin. After three minutes, the liquid was discarded and the resin was washed with DMF (5 × 3 mL), DCM (5 × 3 mL) and DMF (5 × 3 mL). The resin-bound amino acid was subjected to iterative peptide assembly.

Iterative Fmoc-SPPS workflow

Deprotection: The resin was treated with piperidine/DMF (2:8 v:v, 2 × 3 min) and washed with DMF (5 × 3 mL), DCM (5 × 3 mL) and DMF (5 × 3 mL).

General amino acid coupling: A solution of Fmoc-protected amino acid (4 equiv), PyBOP (4 equiv) and DIPEA (8 equiv) in DMF was added to the resin. After 2-16 hours, the resin was washed with DMF (5 × 3 mL), DCM (5 × 3 mL) and DMF (5 × 3 mL).

Capping: A solution of Ac_2O/pyridine (1:9 v:v) was added to the resin. After three minutes, the liquid was discarded and the resin was washed with DMF (5 × 3 mL), DCM (5 × 3 mL) and DMF (5 × 3 mL).

Cleavage: The resin was washed with DCM (10 × 3 mL) to remove any traces of DMF. A mixture of TFA/*i*$PrSi_3H/H_2O$ (90:5:5 v:v:v, 3 mL) was added to the resin and agitated at room temperature for three hours, at which time the resin was washed with TFA (2 × 2 mL) and DCM (2 × 2 mL).

Workup/purification: The cleavage and wash solutions were concentrated by gently blowing over the combined solutions with air. Ice-cold diethyl ether was added to the residue, and the precipitated crude peptide was pelleted by centrifugation. The diethyl ether solution was removed, and the pellet was dried to afford a colorless-yellow powder. The crude peptide was subsequently purified by reverse phase HPLC.

Loading and/or coupling of Fmoc-protected cysteine amino acids: A solution of Fmoc-protected cysteine amino acid (3 equiv), Oxyma pure (3 equiv) and *N,N'*-diisopropylcarbodiimide (3 equiv) in DMF was added to the resin at 4 °C. The coupling reaction was performed at 4 °C for 2-16 hours, at which time the resin was washed with DMF (5 × 3 mL), DCM (5 × 3 mL) and DMF (5 × 3 mL).

Deprotection procedures

Alloc and Allyl protecting groups: The resin (1 equiv) was treated with a solution of $Pd(PPh_3)_4$ (0.5 equiv) and $PhSiH_3$ (2 equiv) in DCM for one hour, at which time the reaction vessel was carefully vented and the dark liquid was discarded. The deprotection reaction was monitored by LCMS and repeated if necessary.

Mmt protecting group: The resin was washed with DCM (5 × 3 mL). The resin was treated with a solution of DCM/TFA/*i*$PrSi_3H$ (95:1:4 v:v:v) and agitated for two minutes at room temperature, at which the time the liquid is discarded. A small portion of resin was treated with TFA to monitor the deprotection. The cleavage reaction was repeated until the Mmt cation was not detected (intense orange color).

S-(*t*Bu) protecting group: The resin was washed with DMF (5 × 3 mL) and was agitated with a solution of DTT (5 equiv) dissolved in DMF/DIPEA/H_2O (95:2.5:2.5 v:v:v) for 10 minutes at RT, at which time the liquid was discarded. The reaction was repeated three more times or until the deprotection was complete, indicated by LCMS analysis.

Compound **3**

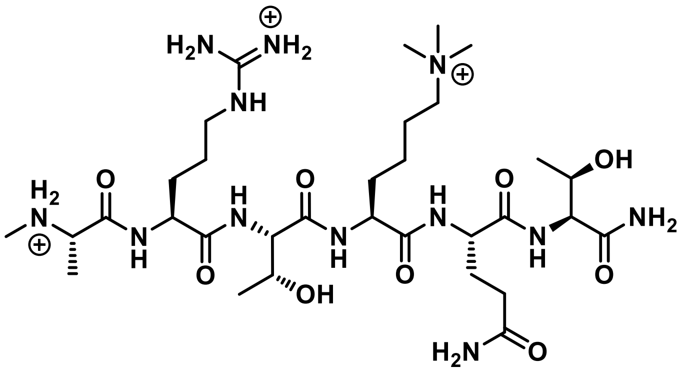

Yield: 8%

Exact mass: 761.50 $[C_{32}H_{65}N_{12}O_9]^{3+}$

HPLC trace (Method 1):

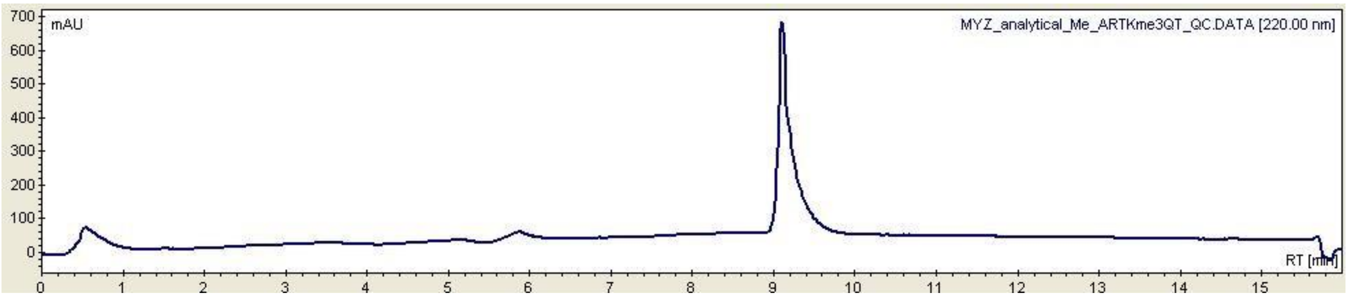

LCMS

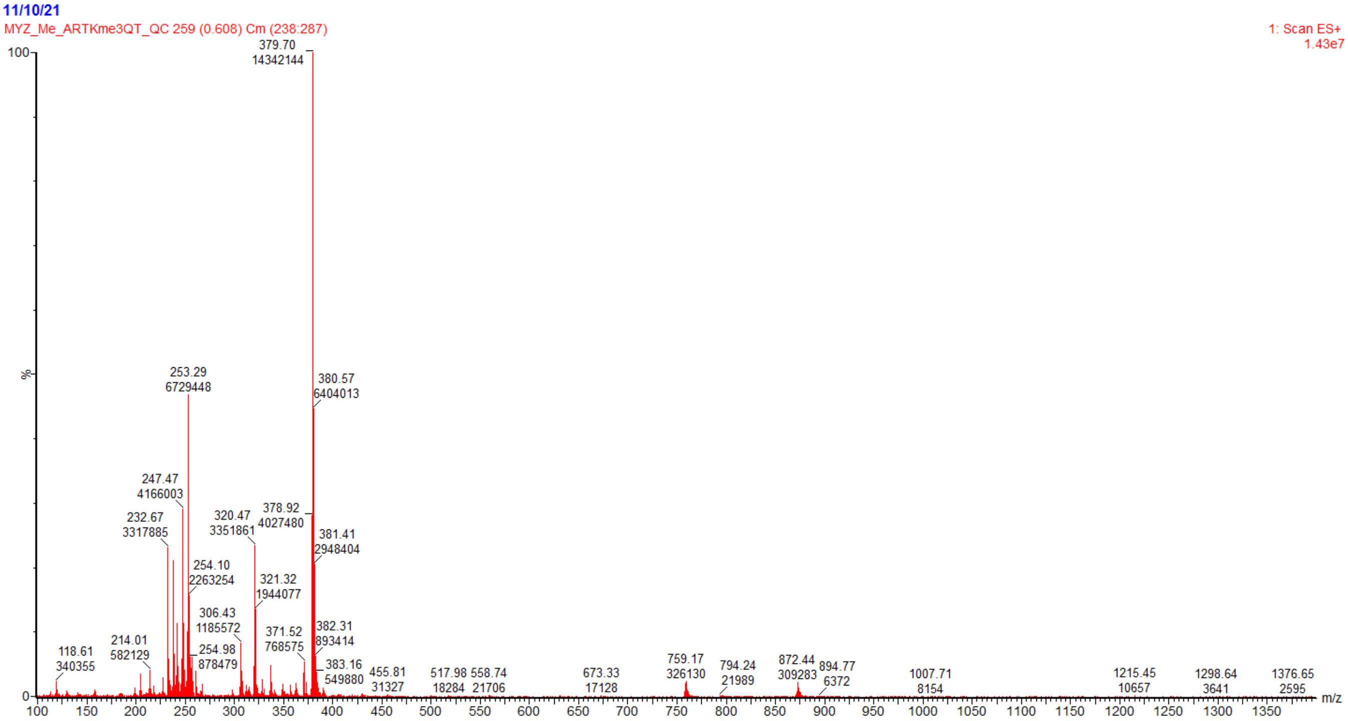

MALDI

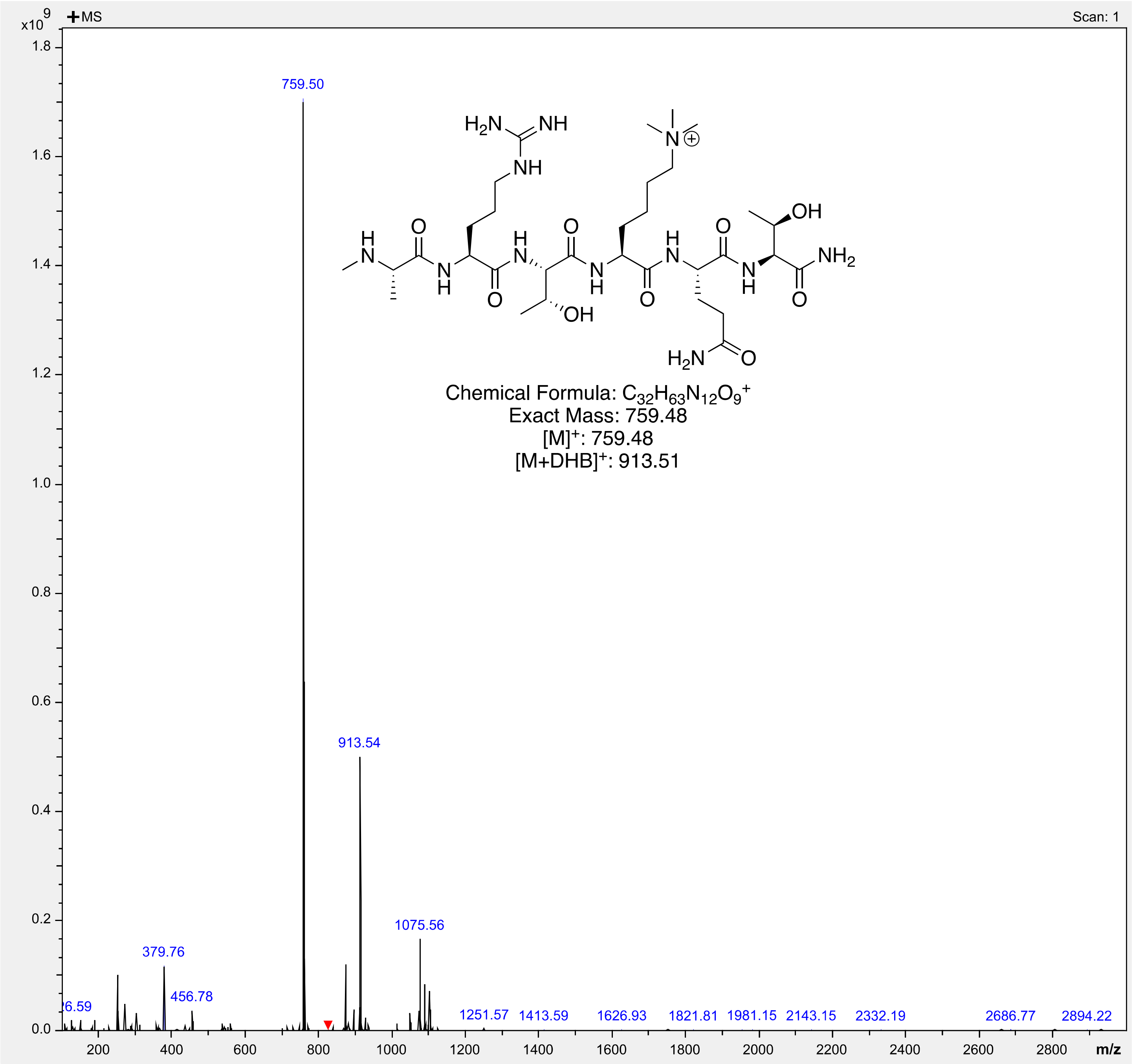

+MS
Scan: 1
x10^9
759.50
913.54
1075.56
379.76
456.78
6.59
1251.57
1413.59
1626.93
1821.81
1981.15
2143.15
2332.19
2686.77
2894.22
m/z
Chemical Formula: C32H63N12O9+
Exact Mass: 759.48
[M]+: 759.48
[M+DHB]+: 913.51

Compound **4**

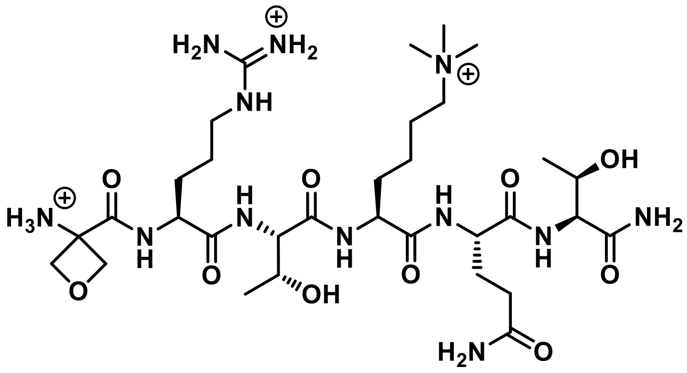

Yield: 8%

Exact mass: 775.48 $[C_{32}H_{63}N_{12}O_{10}]^{3+}$

HPLC trace (Method 1):

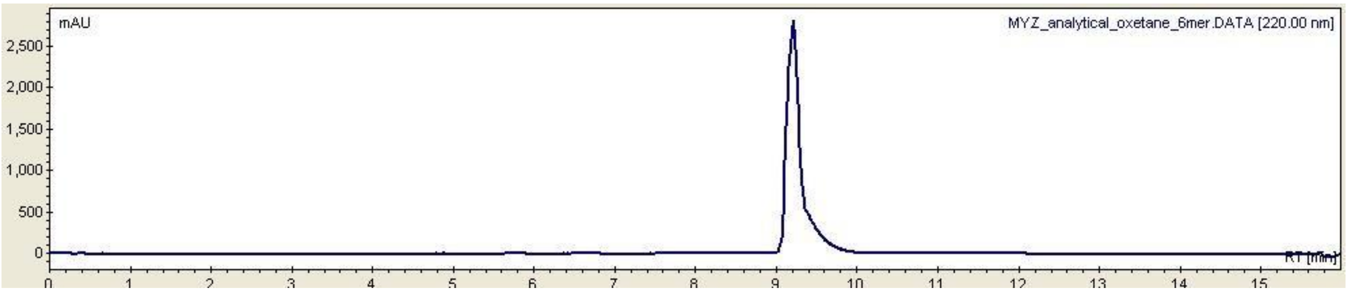

LCMS

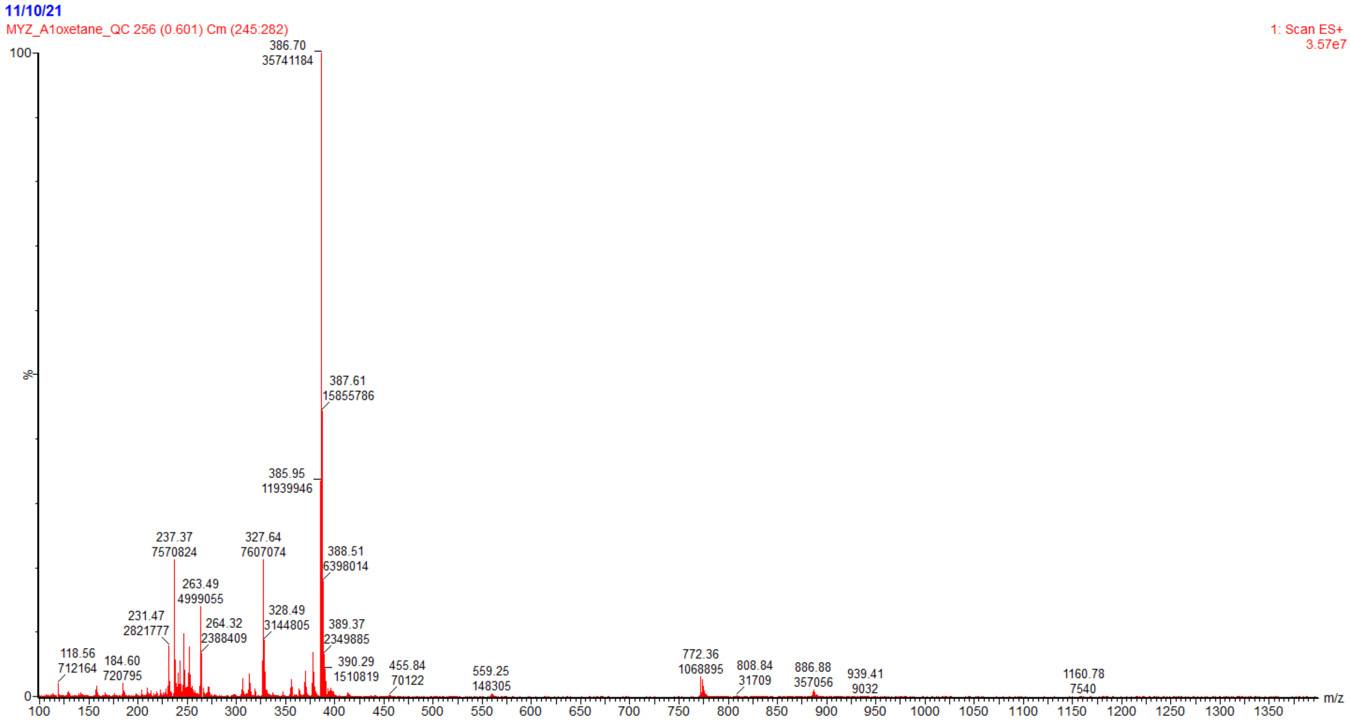

MALDI

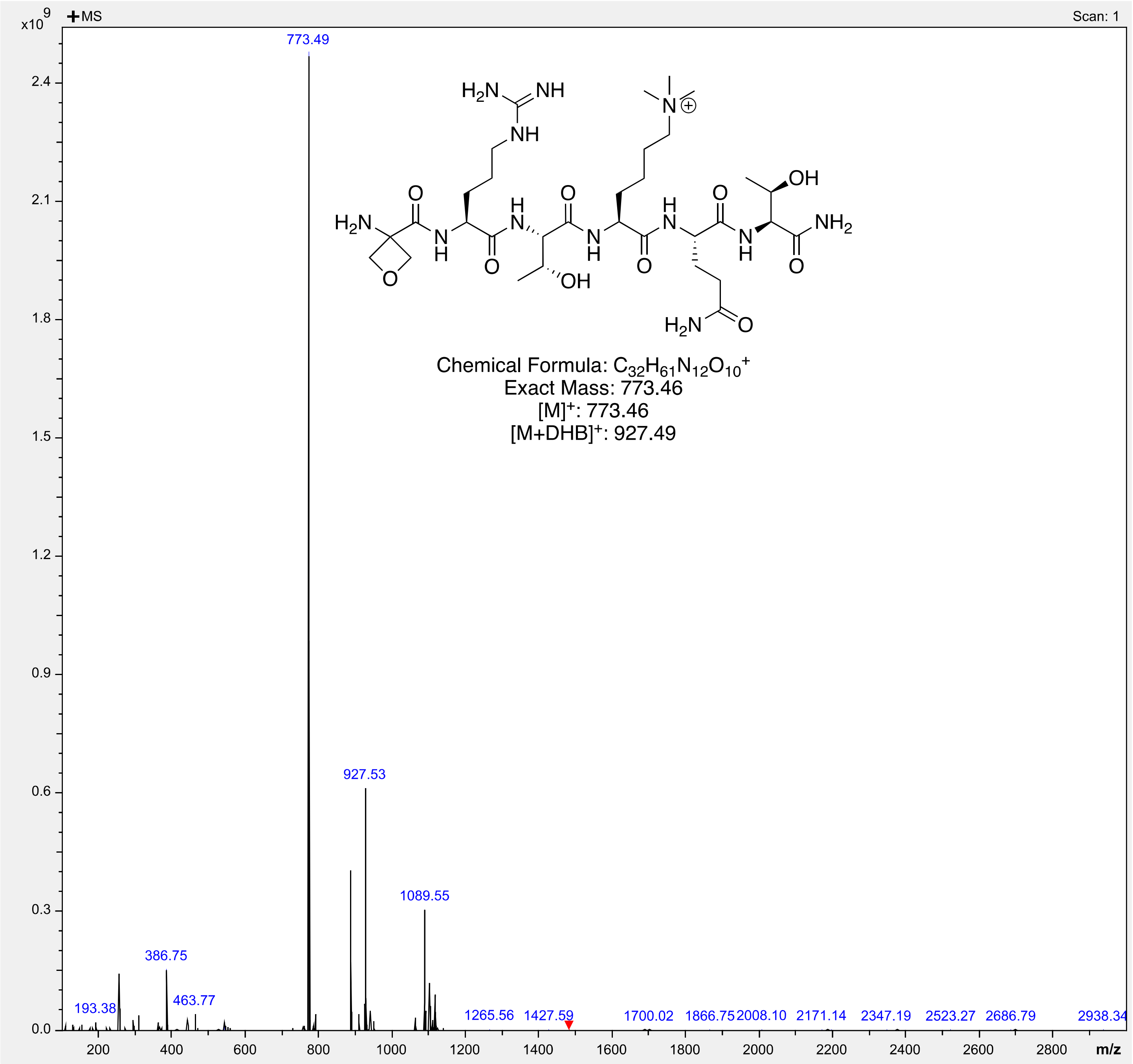

x10^9
+MS
Scan: 1
773.49
927.53
1089.55
386.75
463.77
193.38
1265.56
1427.59
1700.02
1866.75
2008.10
2171.14
2347.19
2523.27
2686.79
2938.34
m/z
Chemical Formula: C32H61N12O10+
Exact Mass: 773.46
[M]+: 773.46
[M+DHB]+: 927.49

Compound **8**

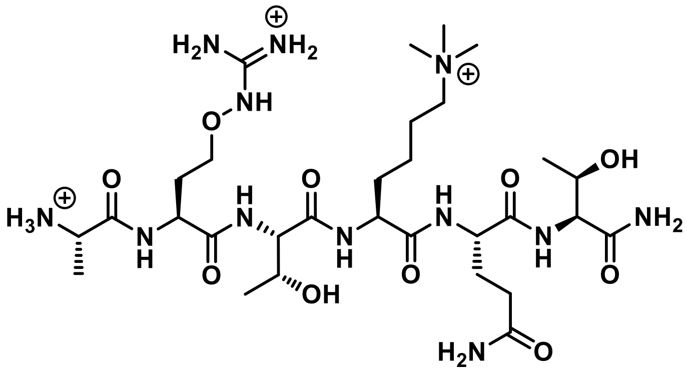

Yield: 17%

Exact mass: 749.46 $[C_{30}H_{61}N_{12}O_{10}]^{3+}$

HPLC trace (Method 1):

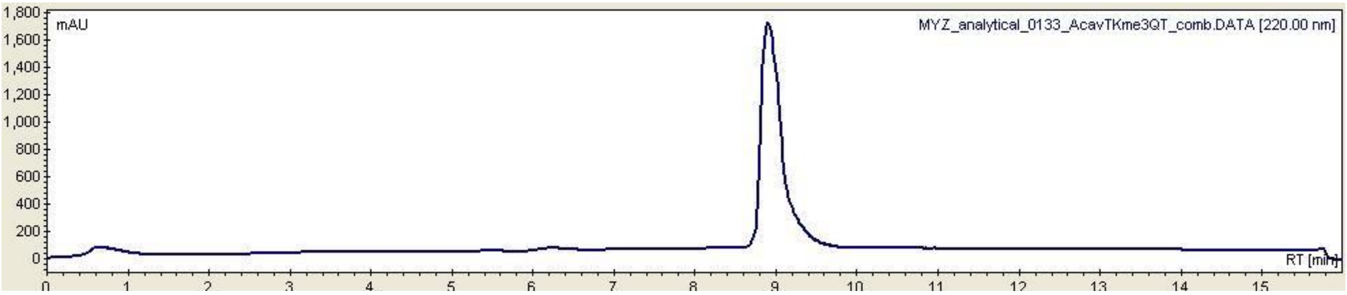

LCMS

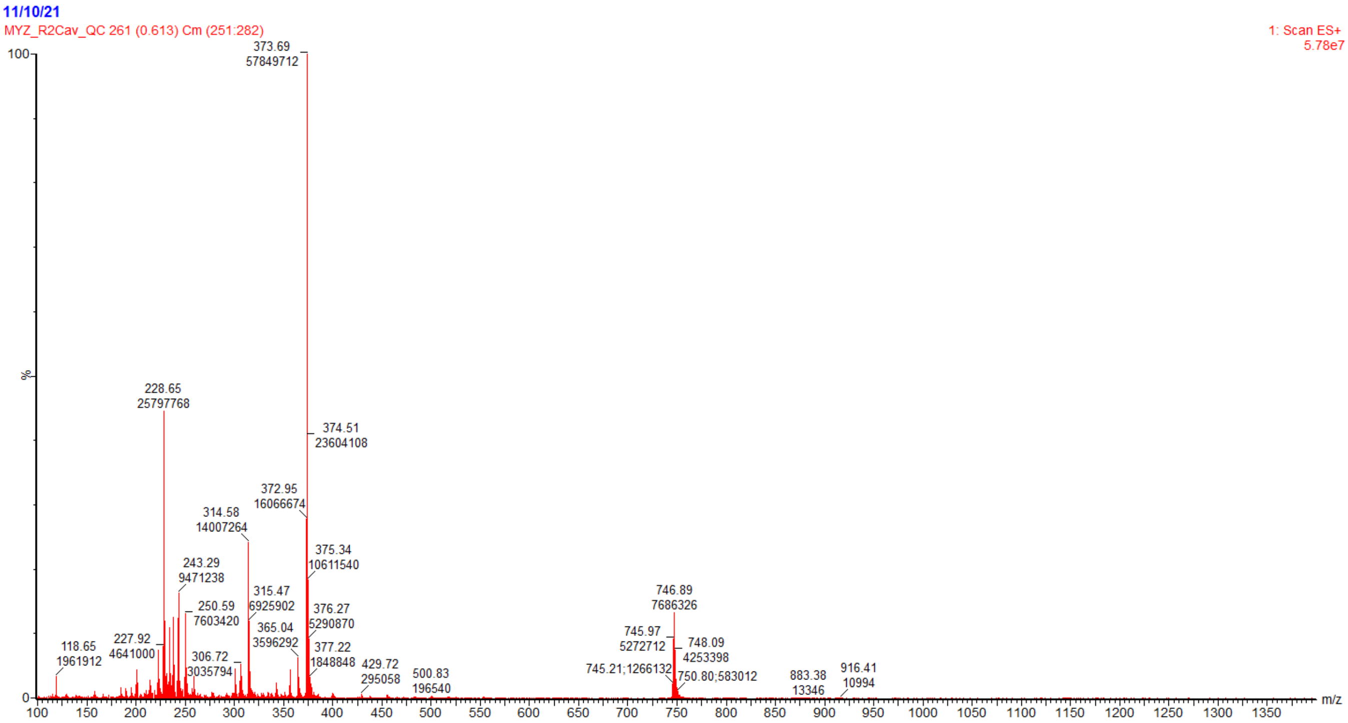

MALDI

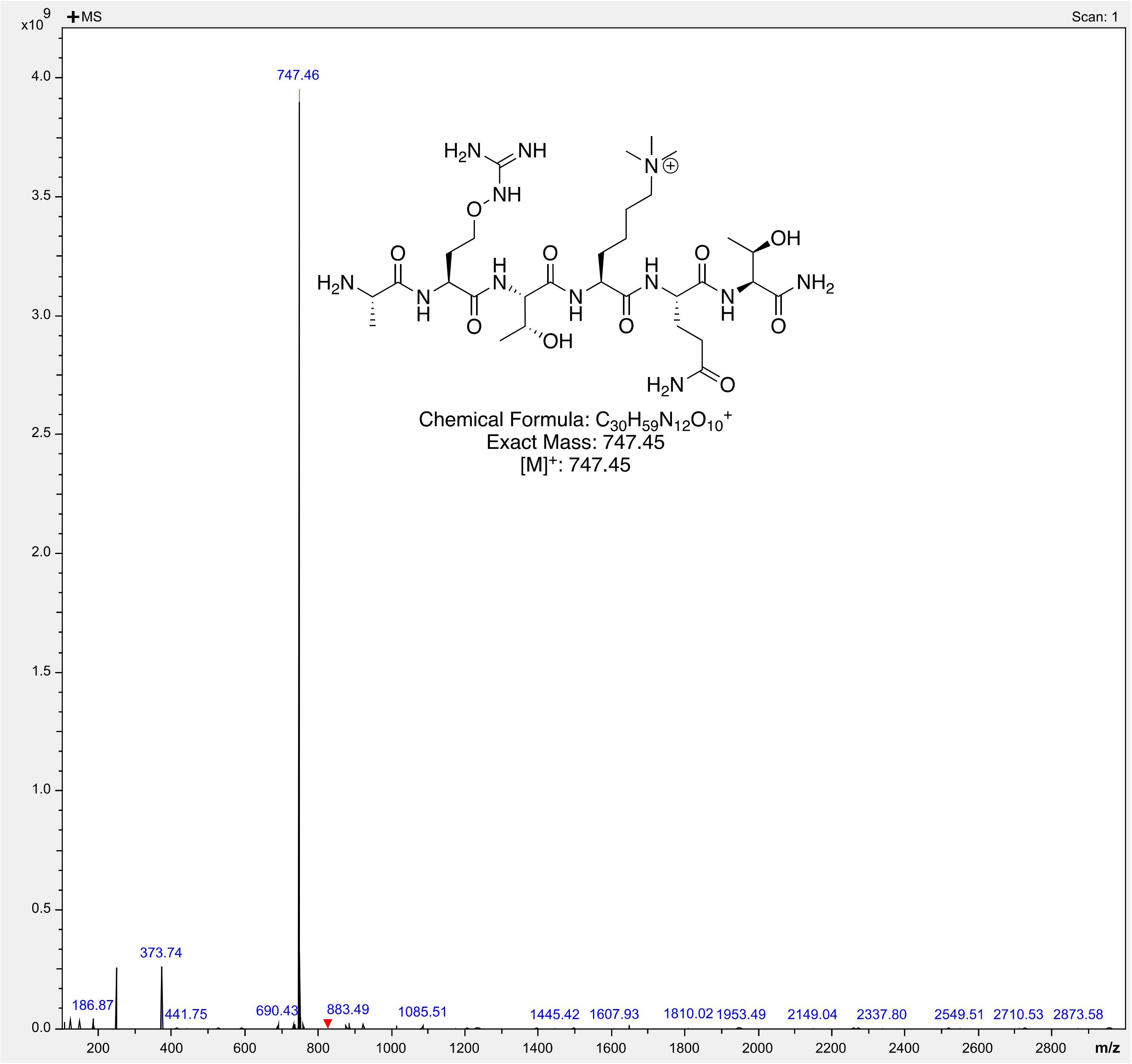
x10^9
+MS
Scan: 1
4.0
3.5
3.0
2.5
2.0
1.5
1.0
0.5
0.0
747.46
373.74
186.87
441.75
690.43
883.49
1085.51
1445.42
1607.93
1810.02
1953.49
2149.04
2337.80
2549.51
2710.53
2873.58
200
400
600
800
1000
1200
1400
1600
1800
2000
2200
2400
2600
2800
m/z
Chemical Formula: C30H59N12O10+
Exact Mass: 747.45
[M]+: 747.45

Compound **9**

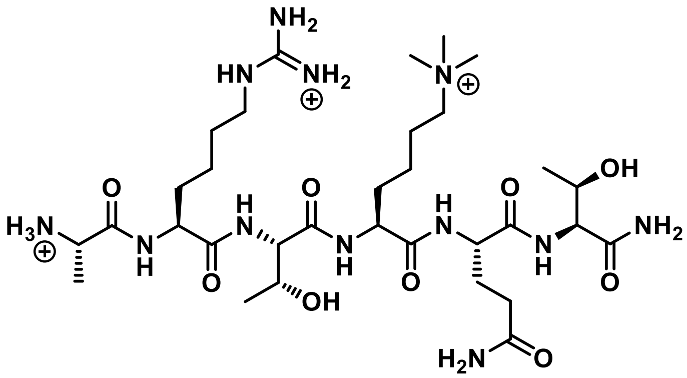

Yield: 12%

Exact mass: 761.50 $[C_{32}H_{65}N_{12}O_9]^{3+}$

HPLC trace (Method 1):

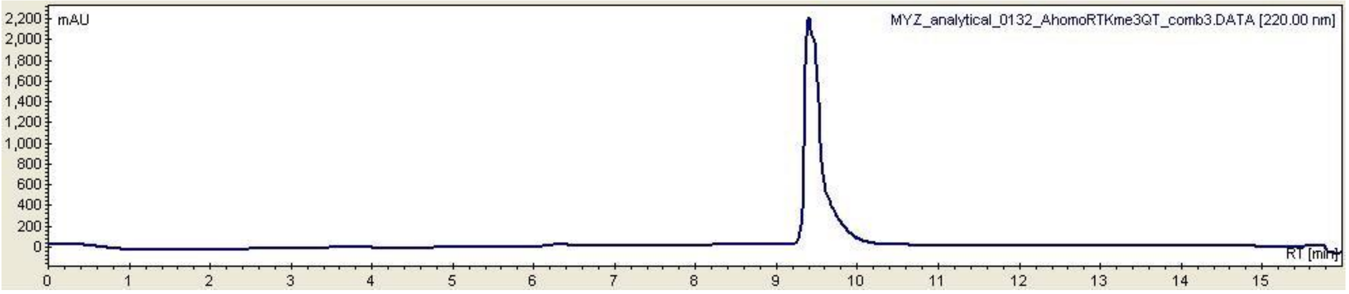

LCMS

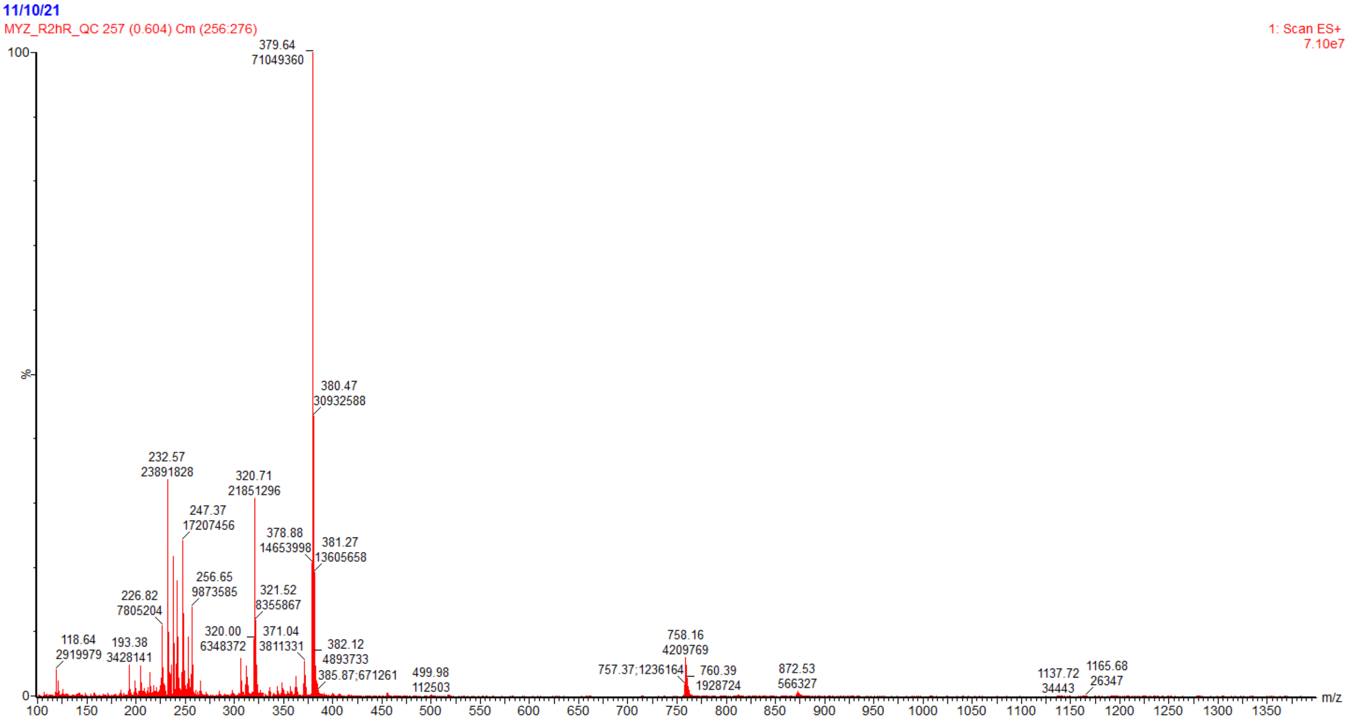

MALDI

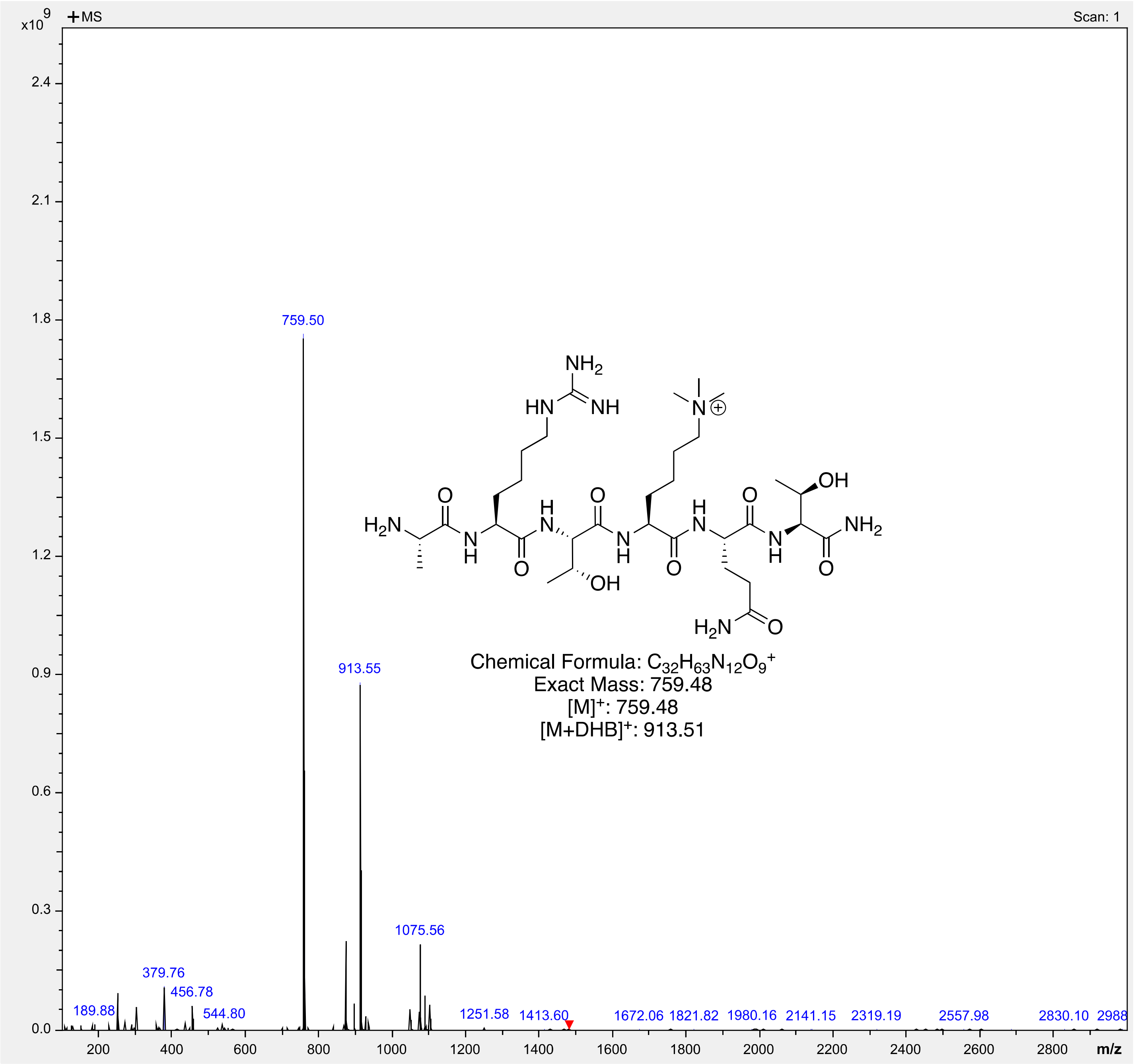

x10^9
+MS
Scan: 1
2.4
2.1
1.8
1.5
1.2
0.9
0.6
0.3
0.0
759.50
913.55
1075.56
379.76
456.78
189.88
544.80
1251.58
1413.60
1672.06
1821.82
1980.16
2141.15
2319.19
2557.98
2830.10
2988
200
400
600
800
1000
1200
1400
1600
1800
2000
2200
2400
2600
2800
m/z
Chemical Formula: C32H63N12O9+
Exact Mass: 759.48
[M]+: 759.48
[M+DHB]+: 913.51

Compound **10**

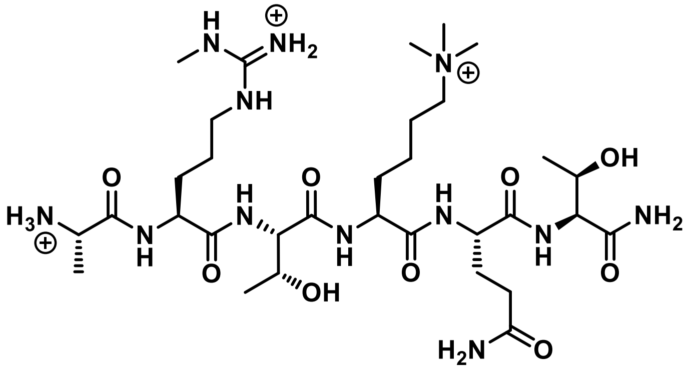

Yield: 22%

Exact mass: 761.50 $[C_{32}H_{65}N_{12}O_9]^{3+}$

HPLC trace (Method 1):

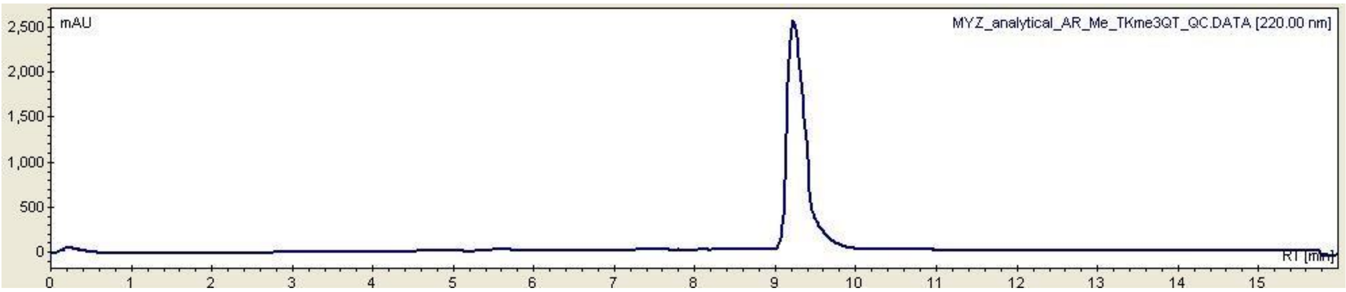

LCMS

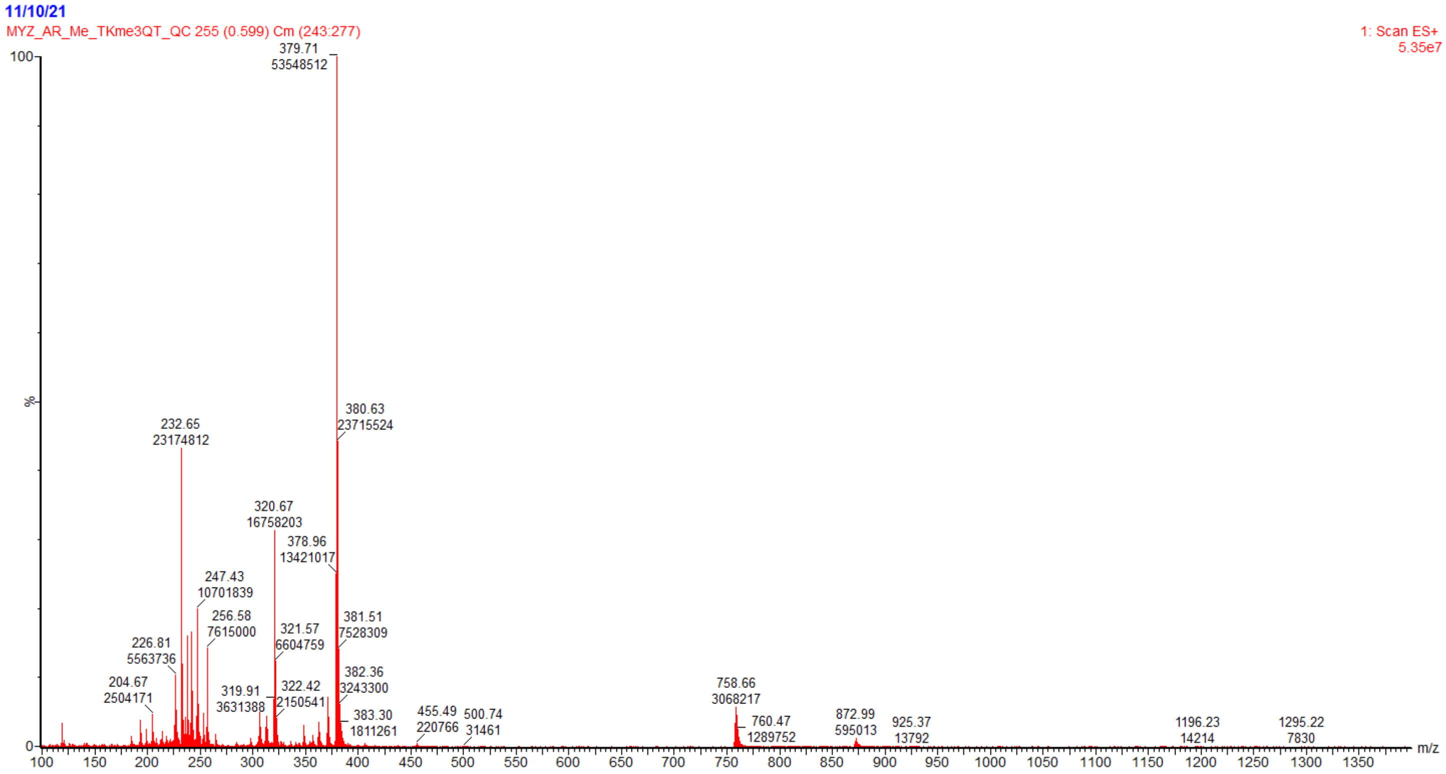

MALDI

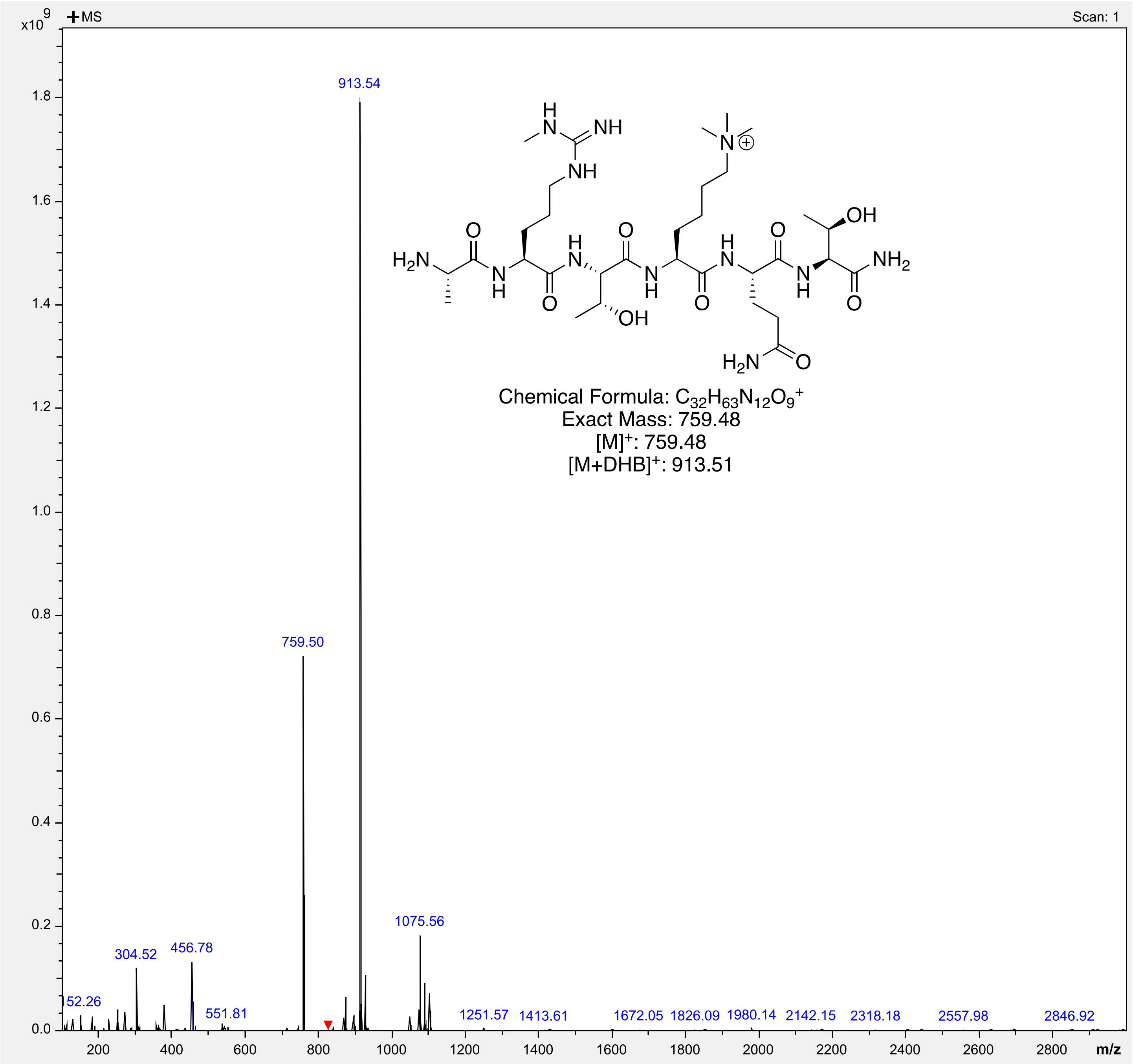

x10^9 +MS
Scan: 1
913.54
759.50
1075.56
304.52
456.78
152.26
551.81
1251.57
1413.61
1672.05
1826.09
1980.14
2142.15
2318.18
2557.98
2846.92
m/z
Chemical Formula: C32H63N12O9+
Exact Mass: 759.48
[M]+: 759.48
[M+DHB]+: 913.51

Compound **11**

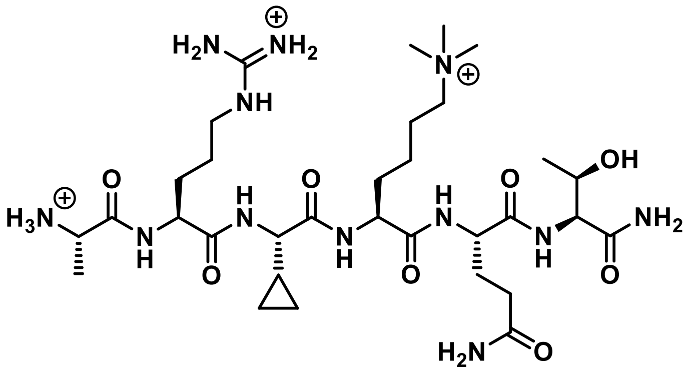

Yield: 6%

Exact mass: 743.39 $[C_{32}H_{63}N_{12}O_8]^{3+}$

HPLC trace (Method 2):

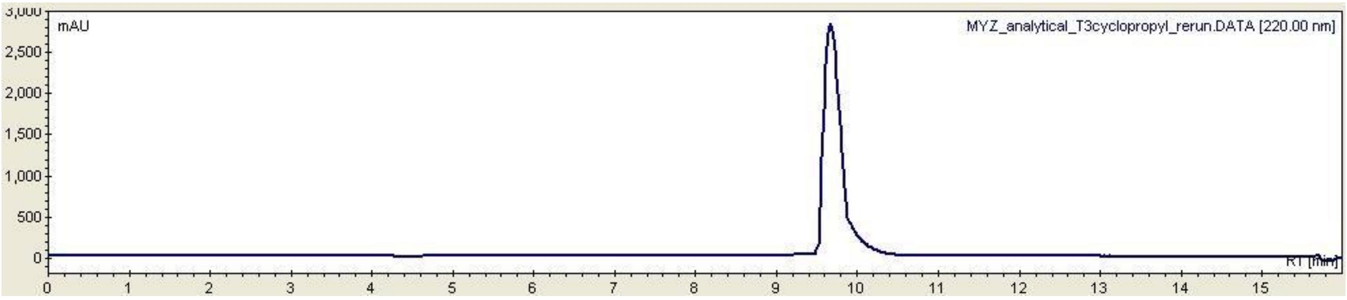

LCMS

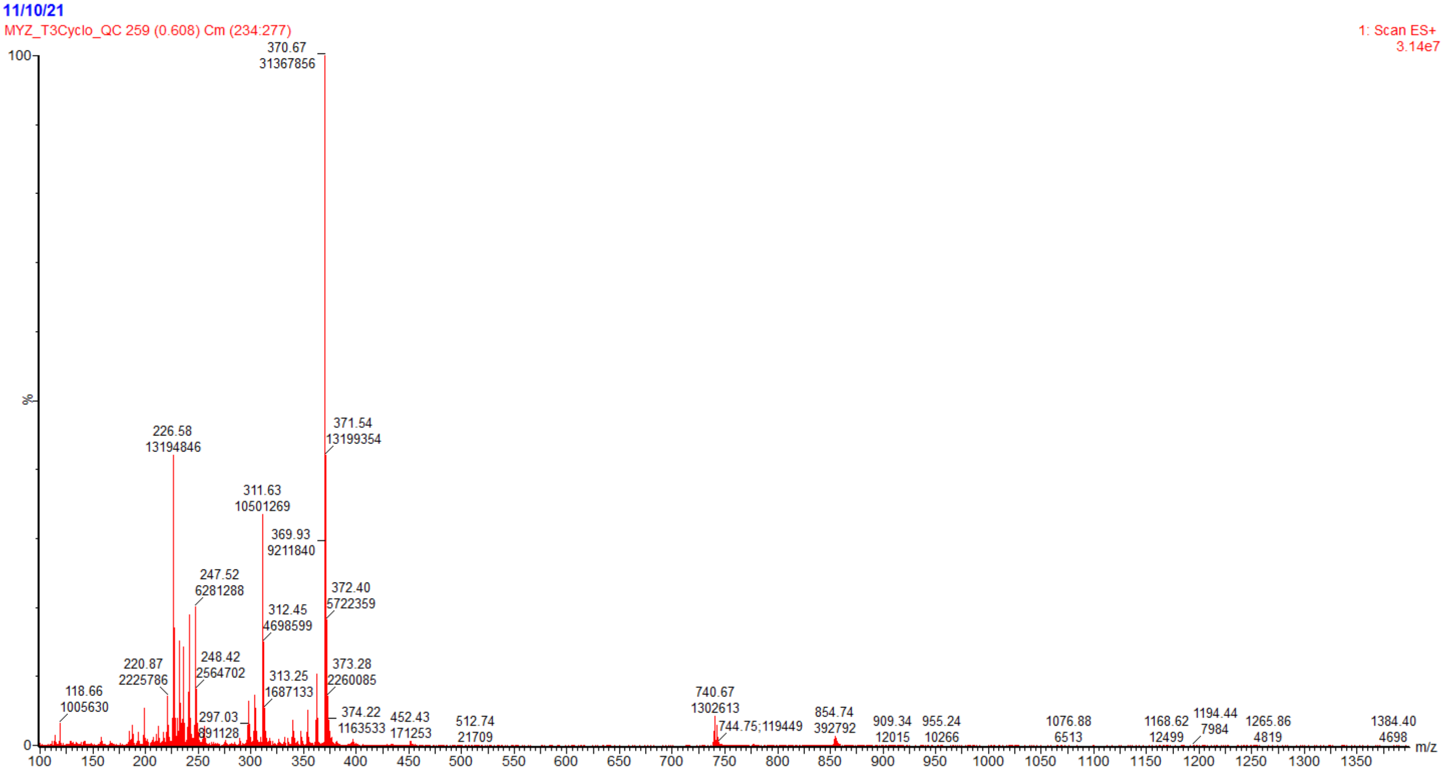

MALDI

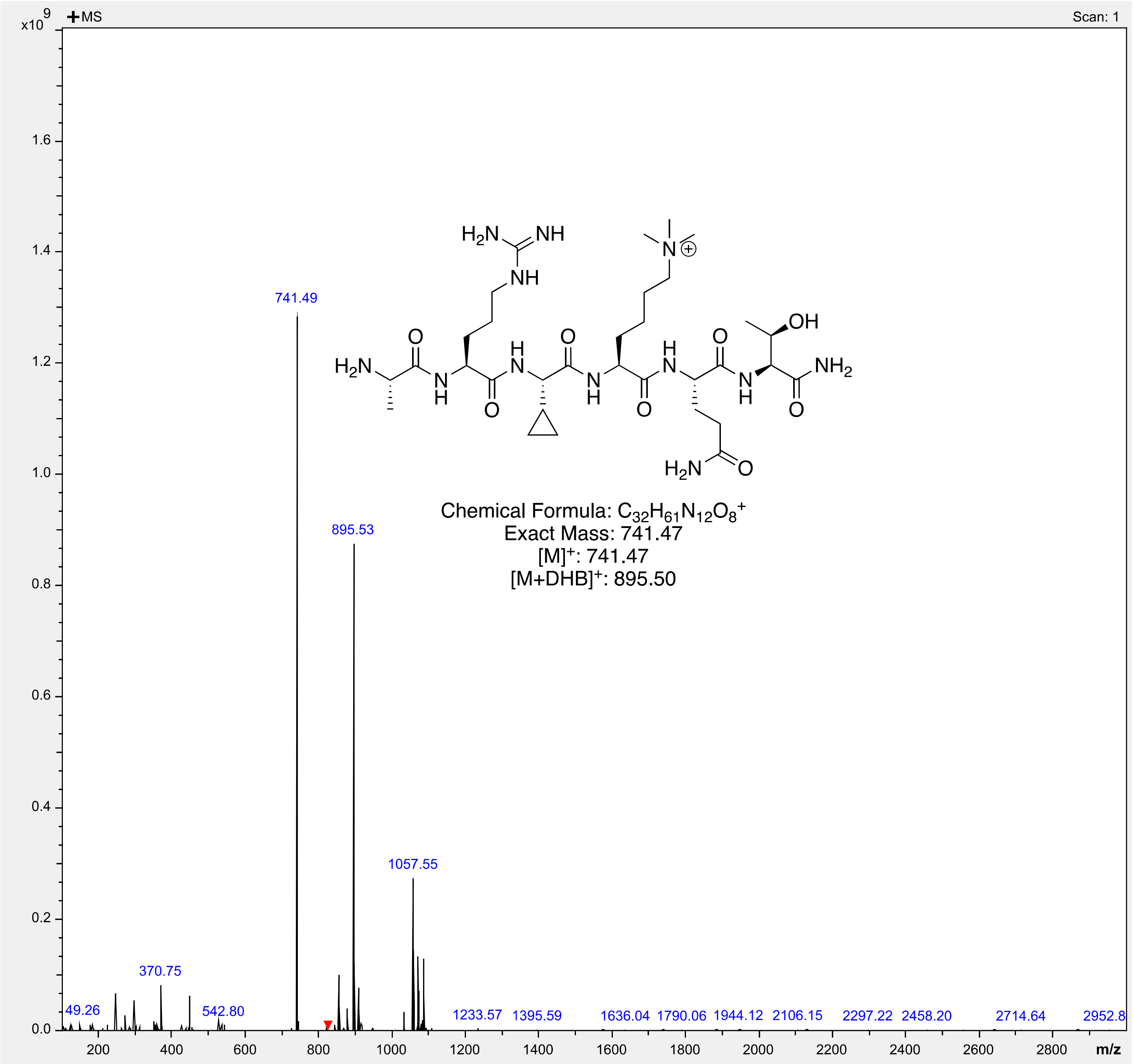

x10^9
+MS
Scan: 1
741.49
895.53
1057.55
370.75
49.26
542.80
1233.57
1395.59
1636.04
1790.06
1944.12
2106.15
2297.22
2458.20
2714.64
2952.8
m/z
Chemical Formula: $C_{32}H_{61}N_{12}O_8^+$
Exact Mass: 741.47
$[M]^+$: 741.47
$[M+DHB]^+$: 895.50

Compound **12**

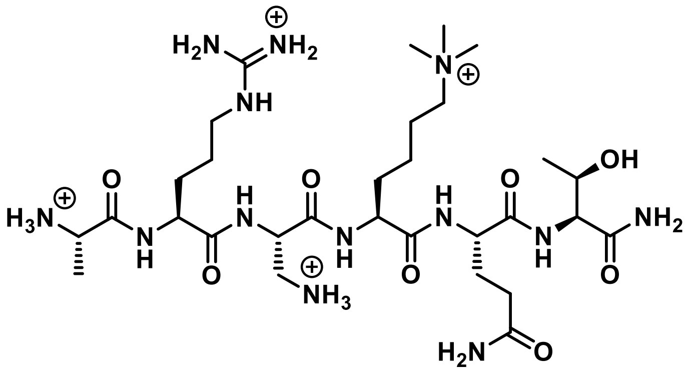

Yield: 15%

Exact mass: 733.49 $[C_{30}H_{63}N_{13}O_8]^{4+}$

HPLC trace (Method 1):

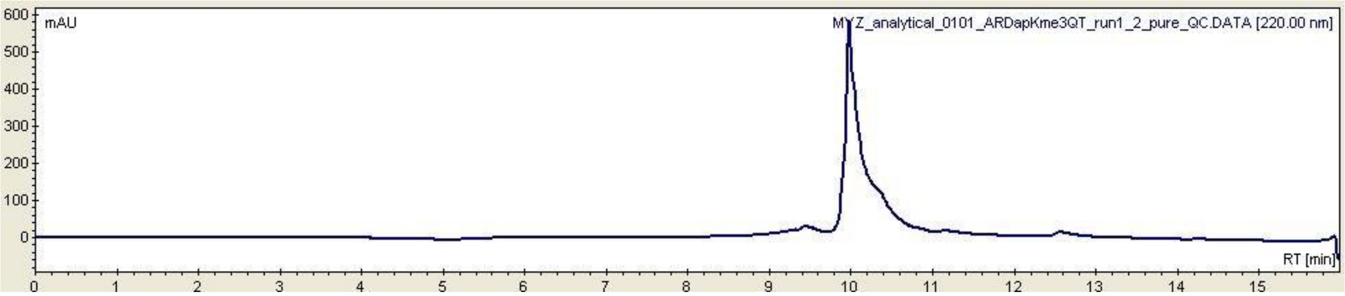

LCMS

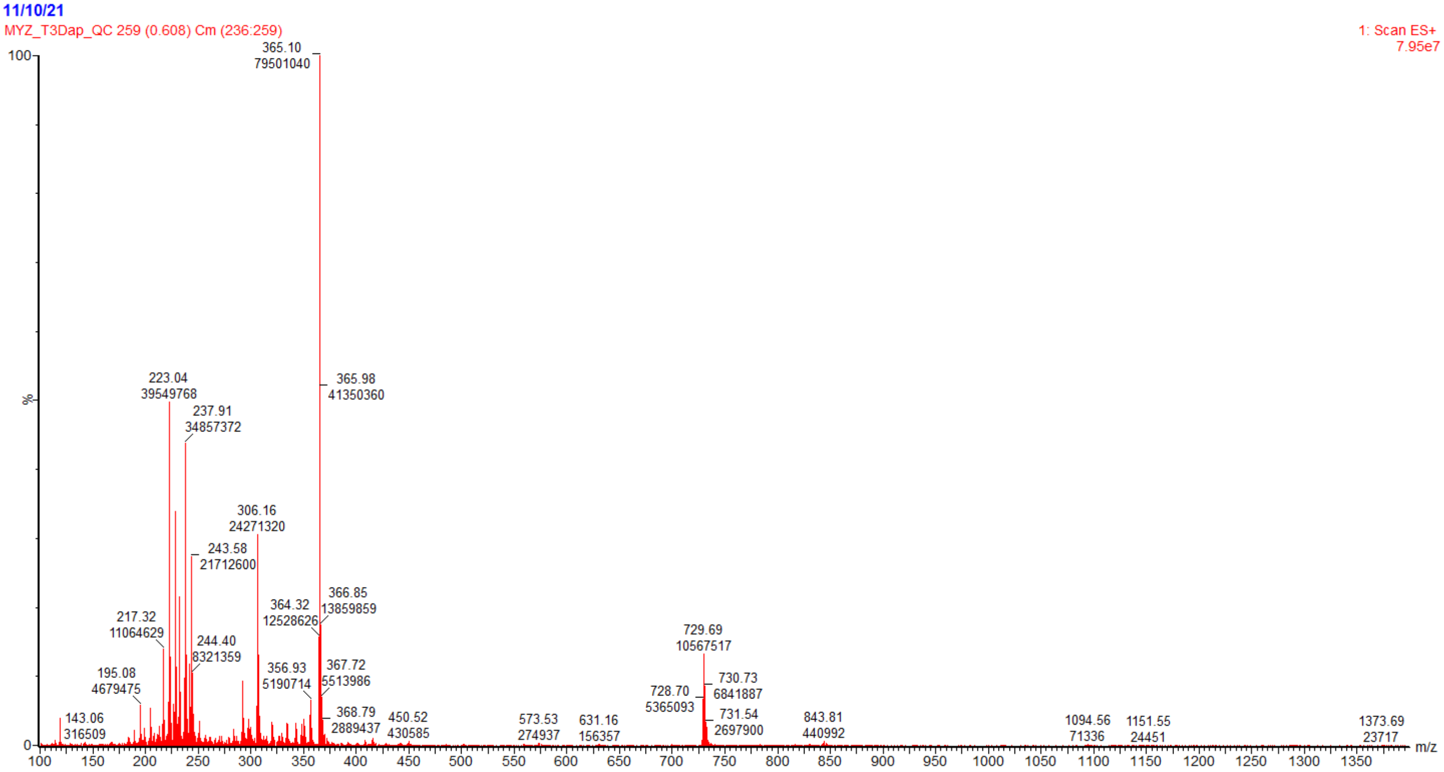

MALDI

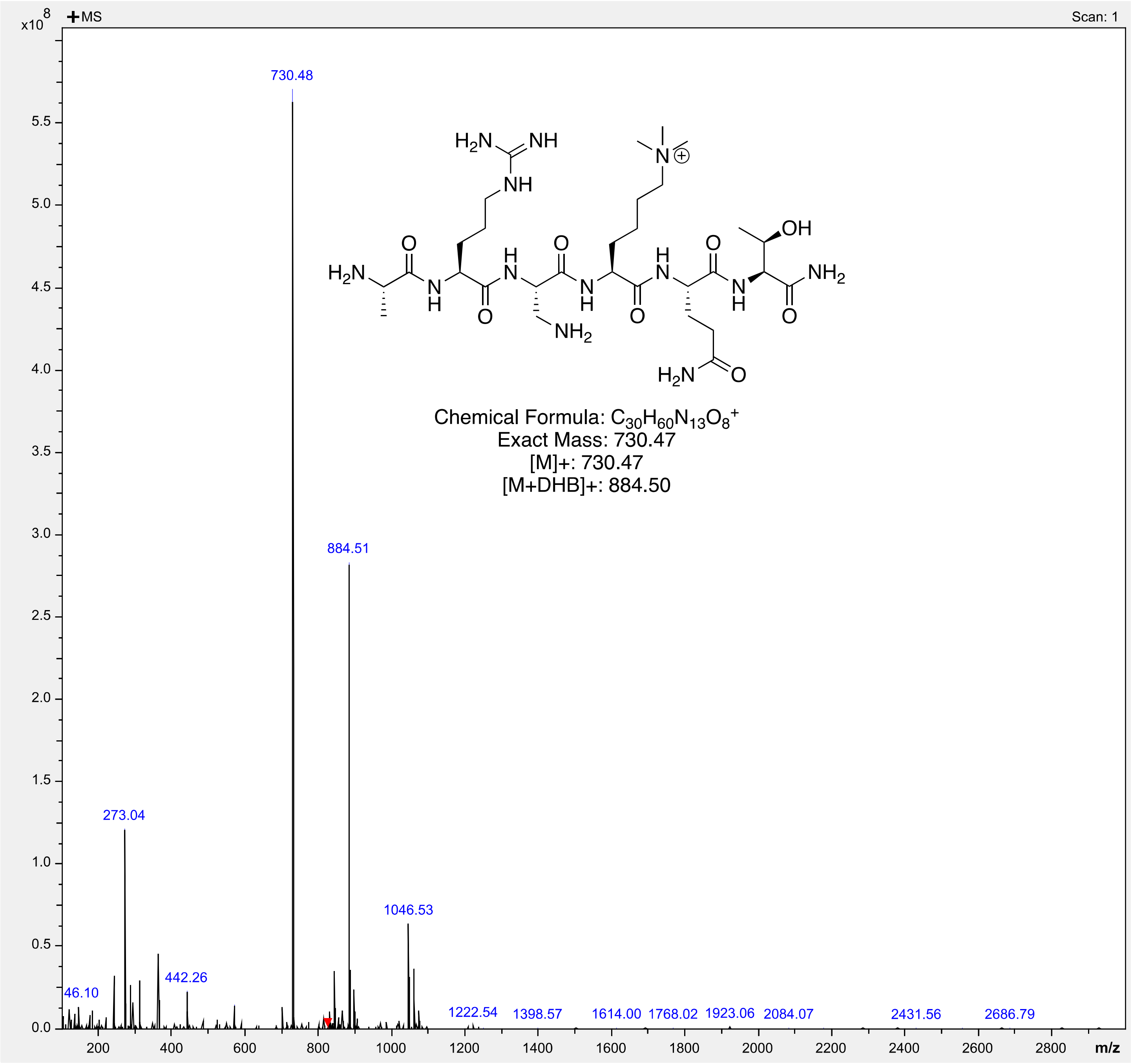

x10^8
+MS
Scan: 1
730.48
884.51
273.04
1046.53
442.26
46.10
1222.54
1398.57
1614.00
1768.02
1923.06
2084.07
2431.56
2686.79
m/z
Chemical Formula: $C_{30}H_{60}N_{13}O_8^+$
Exact Mass: 730.47
[M]+: 730.47
[M+DHB]+: 884.50

Compound **13**

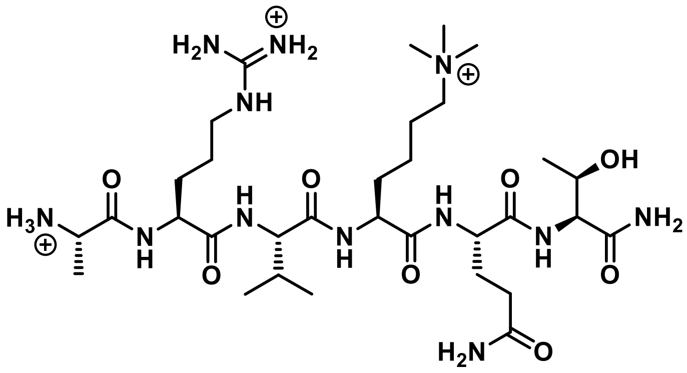

Yield: 17%

Exact mass: 745.50 $[C_{32}H_{65}N_{12}O_8]^{3+}$

HPLC trace (<u>Method 1</u>):

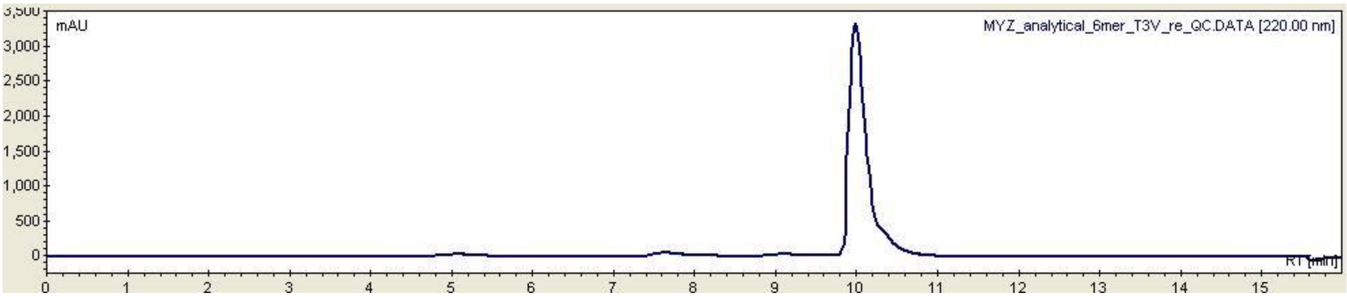

LCMS

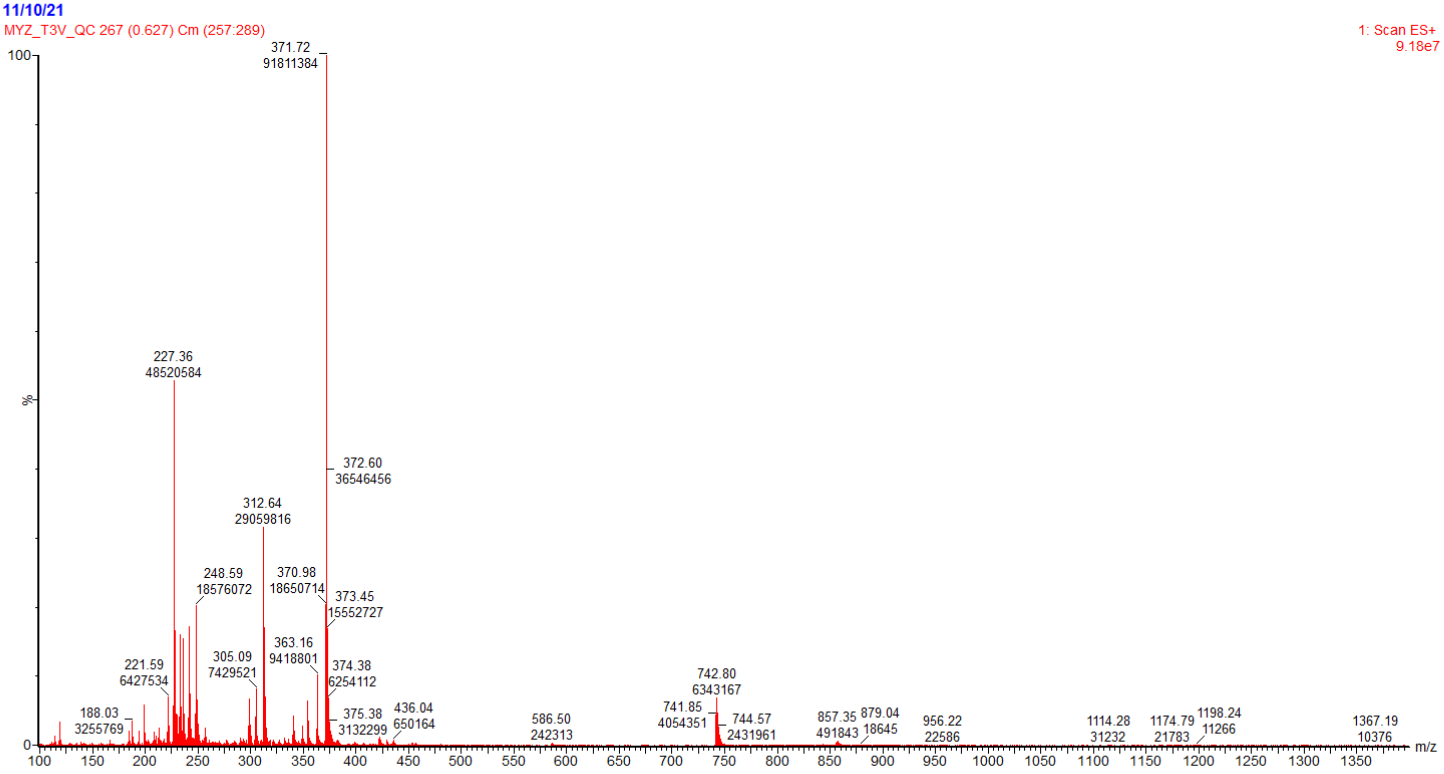

MALDI

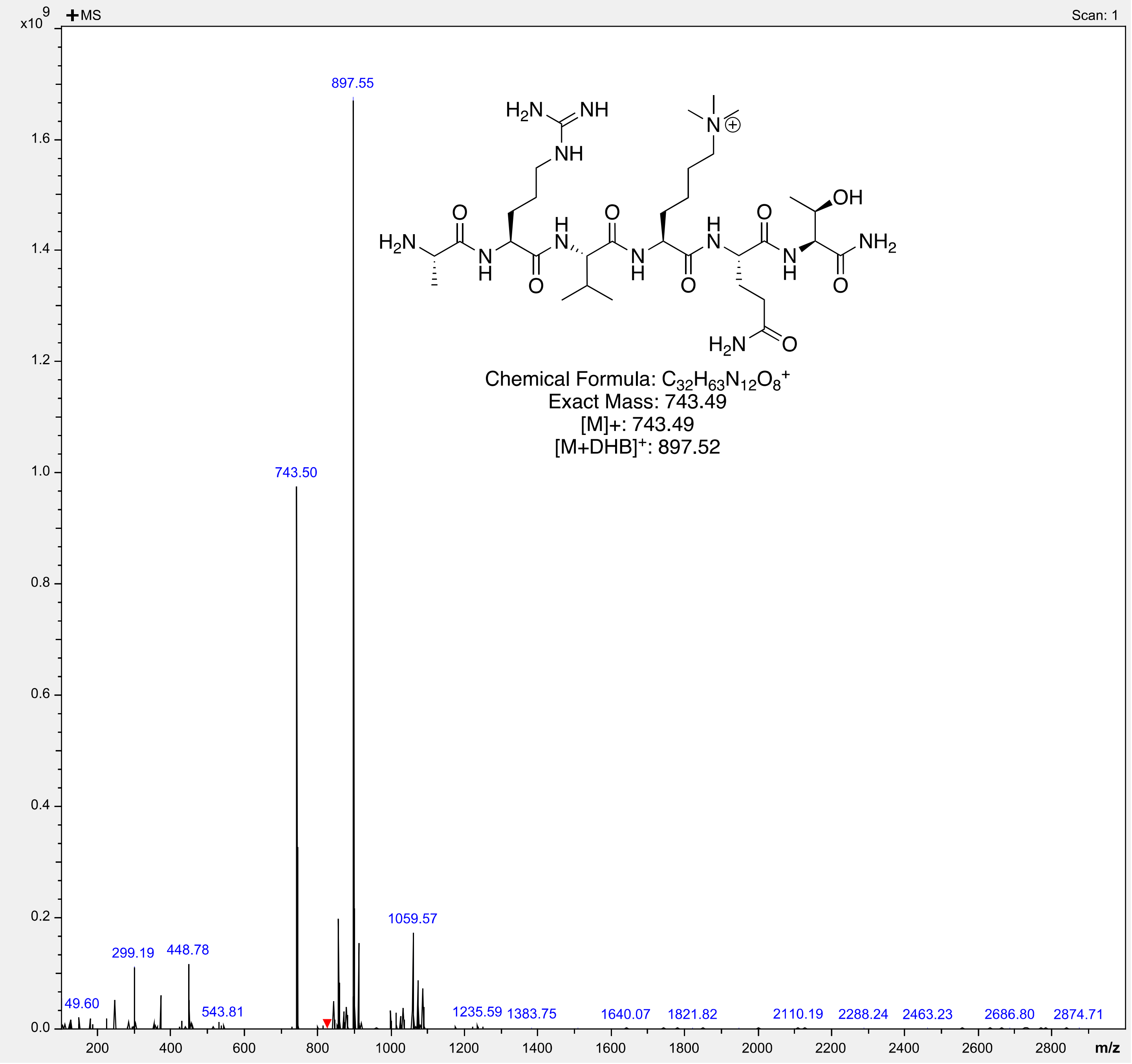

+MS
Scan: 1
x10^9
1.6
1.4
1.2
1.0
0.8
0.6
0.4
0.2
0.0
897.55
743.50
1059.57
299.19
448.78
49.60
543.81
1235.59
1383.75
1640.07
1821.82
2110.19
2288.24
2463.23
2686.80
2874.71
200
400
600
800
1000
1200
1400
1600
1800
2000
2200
2400
2600
2800
m/z
Chemical Formula: C32H63N12O8+
Exact Mass: 743.49
[M]+: 743.49
[M+DHB]+: 897.52

Compound **14**

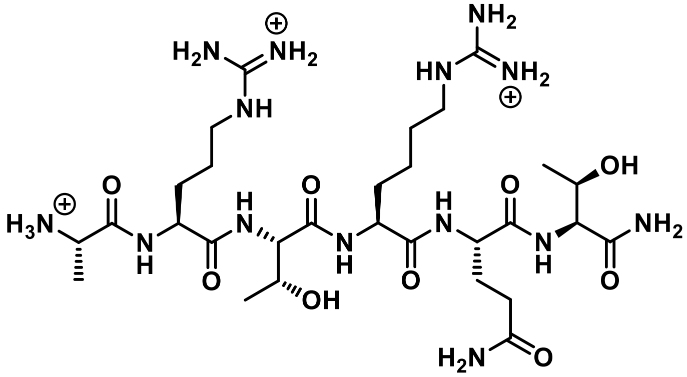

Yield: 26%

Exact mass: 747.46 $[C_{29}H_{59}N_{14}O_9]^{3+}$

HPLC trace (Method 1):

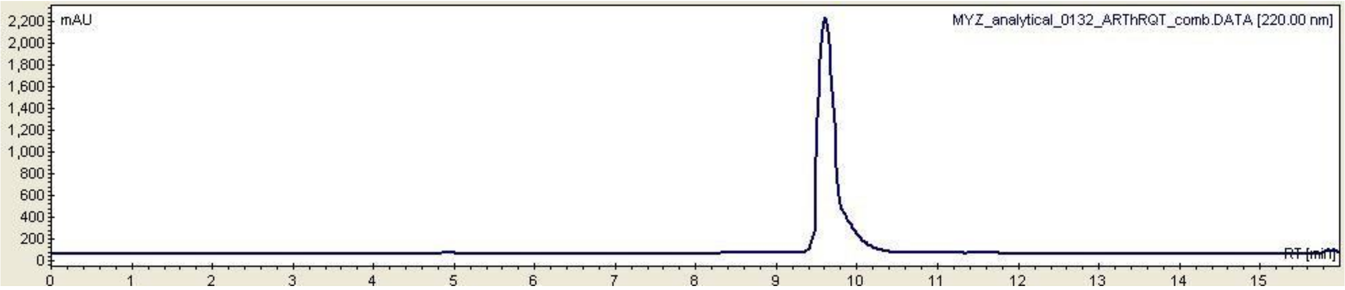

LCMS

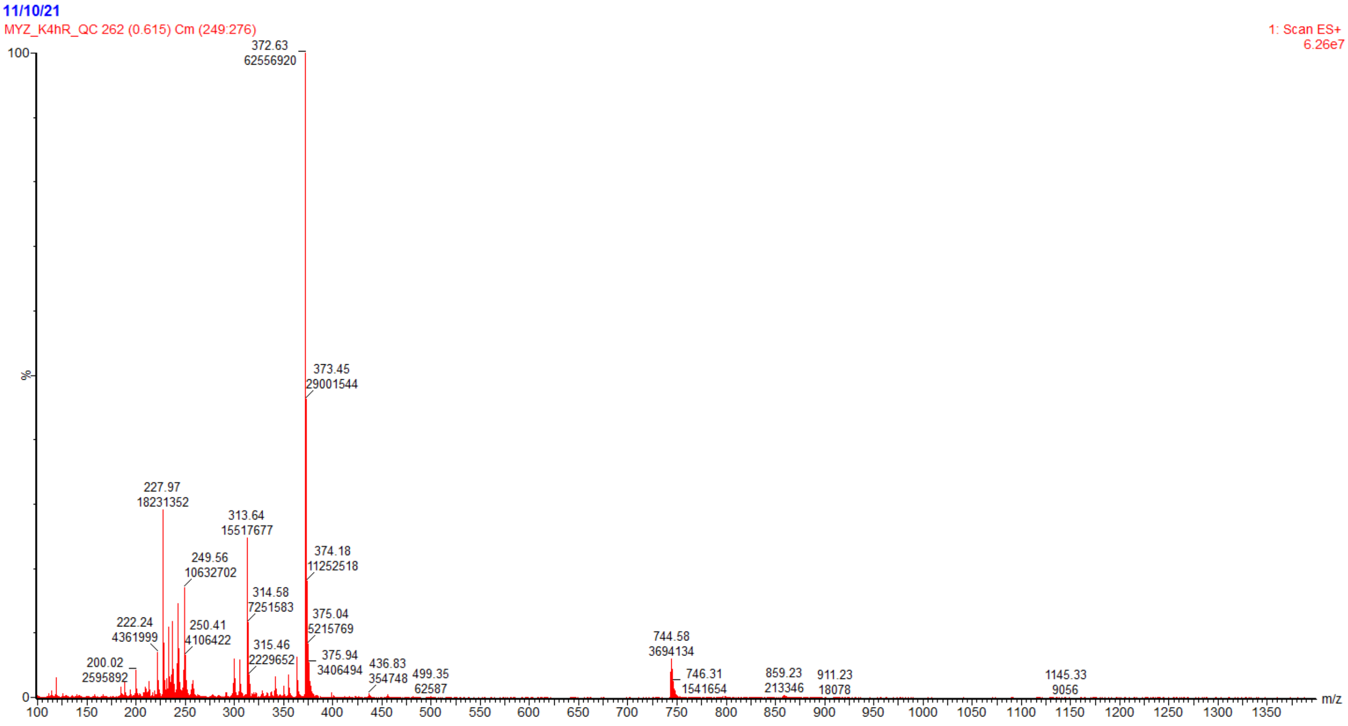

MALDI

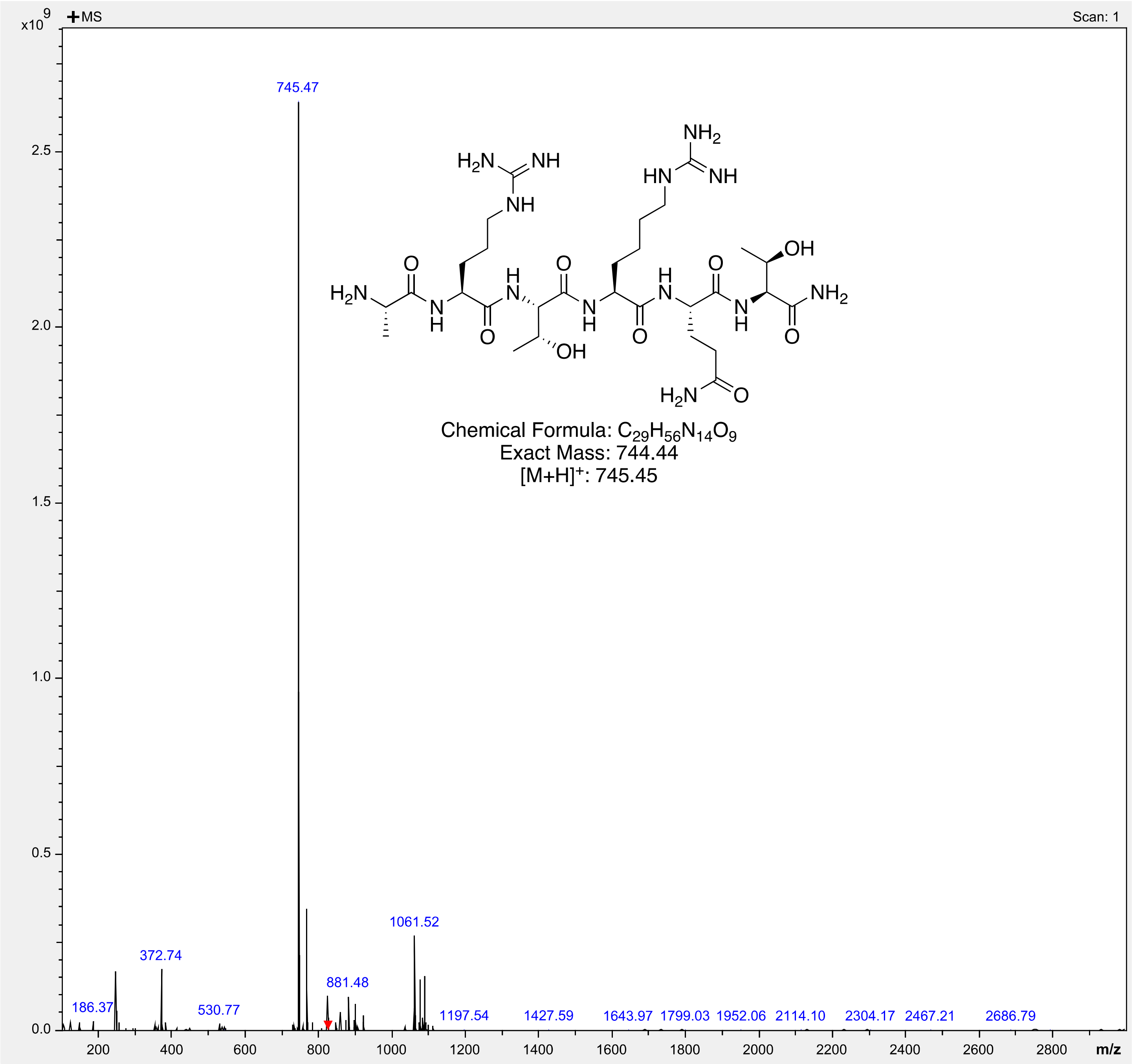

x10^9
+MS
Scan: 1
745.47
2.5
2.0
1.5
1.0
0.5
0.0
372.74
186.37
530.77
881.48
1061.52
1197.54
1427.59
1643.97
1799.03
1952.06
2114.10
2304.17
2467.21
2686.79
200
400
600
800
1000
1200
1400
1600
1800
2000
2200
2400
2600
2800
m/z
Chemical Formula: C29H56N14O9
Exact Mass: 744.44
[M+H]+: 745.45

Compound **15**

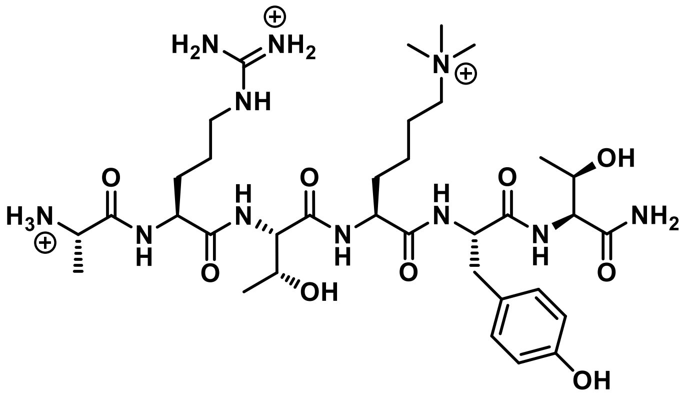

Yield: 8%

Exact mass: 782.49 $[C_{35}H_{64}N_{11}O_9]^{3+}$

HPLC trace (Method 1):

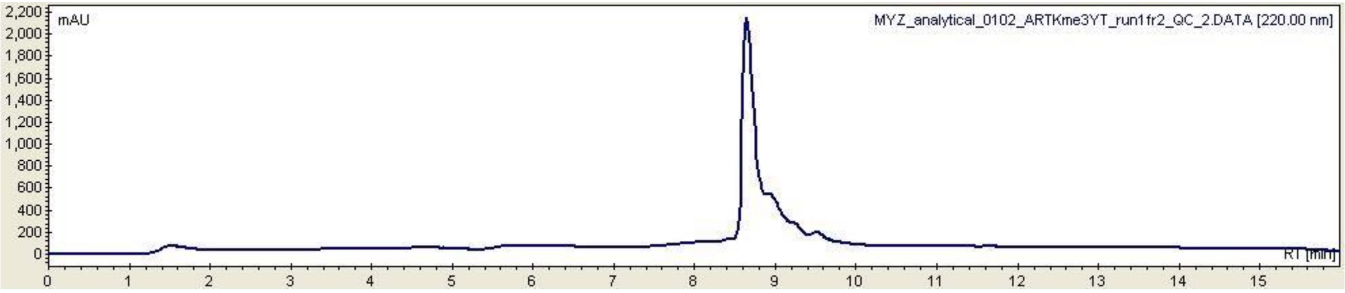

LCMS

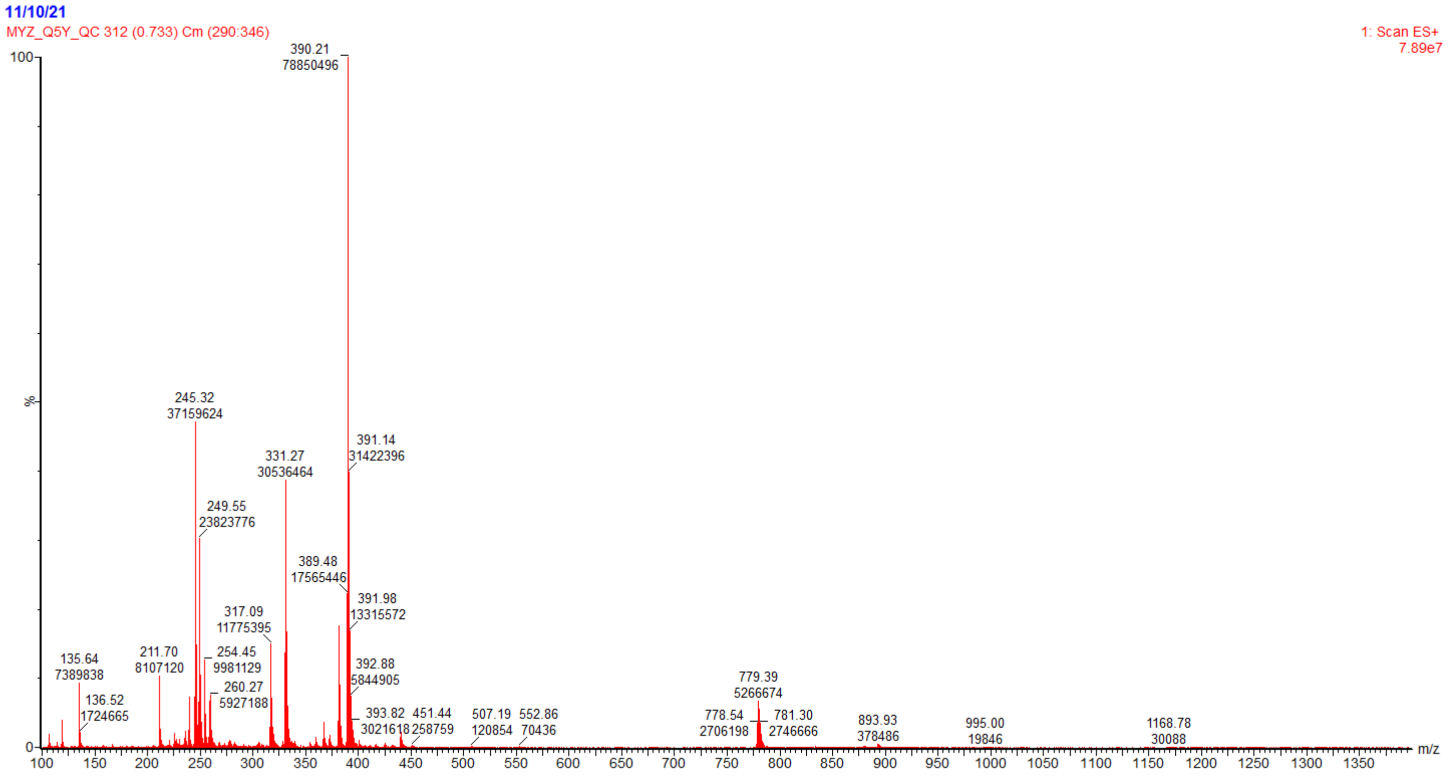

MALDI

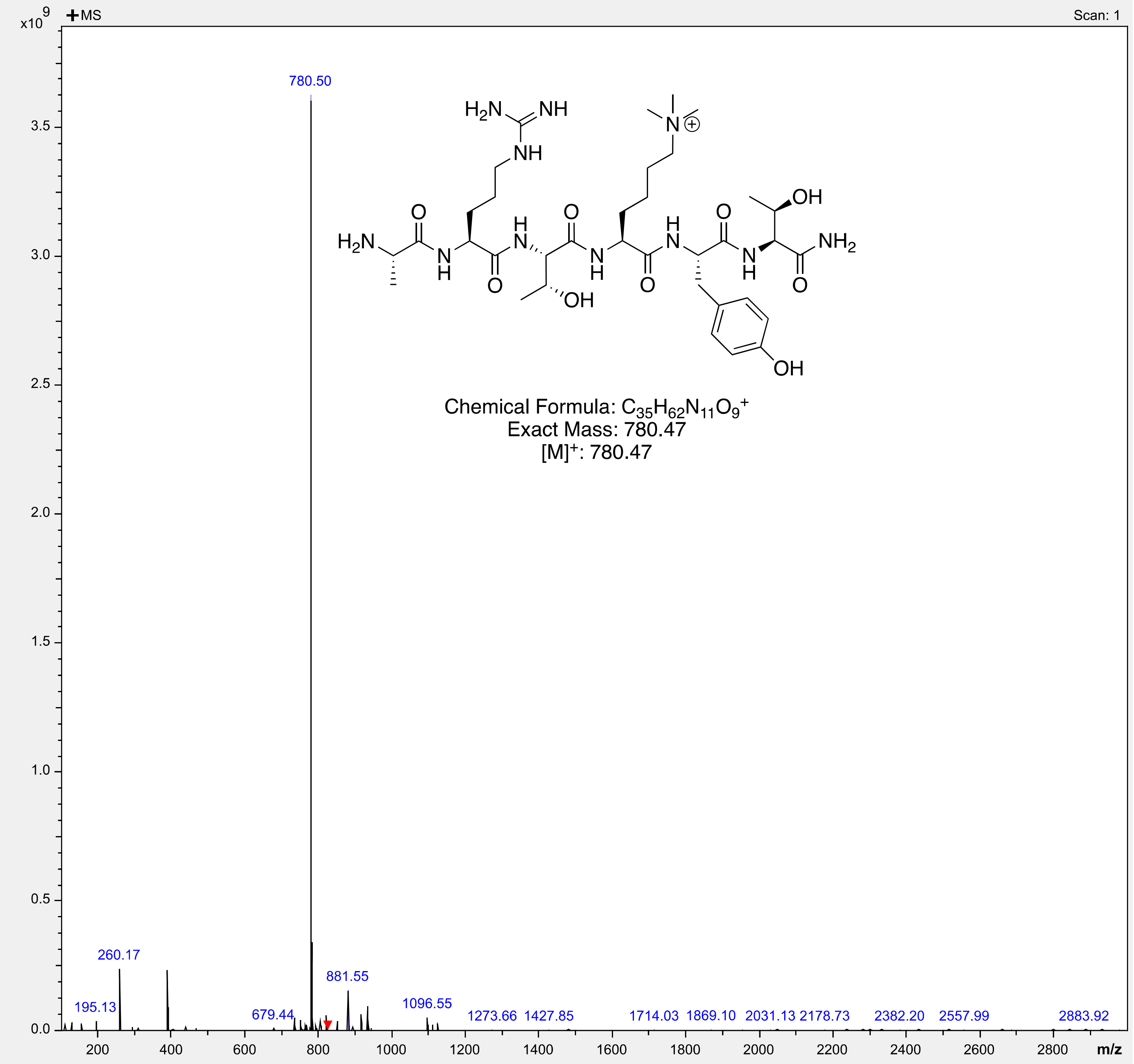

x10^9
+MS
Scan: 1
780.50
260.17
195.13
679.44
881.55
1096.55
1273.66
1427.85
1714.03
1869.10
2031.13
2178.73
2382.20
2557.99
2883.92
m/z
Chemical Formula: C35H62N11O9+
Exact Mass: 780.47
[M]+: 780.47

Macrocyclization Synthetic Procedures

Series B: macrolactamization

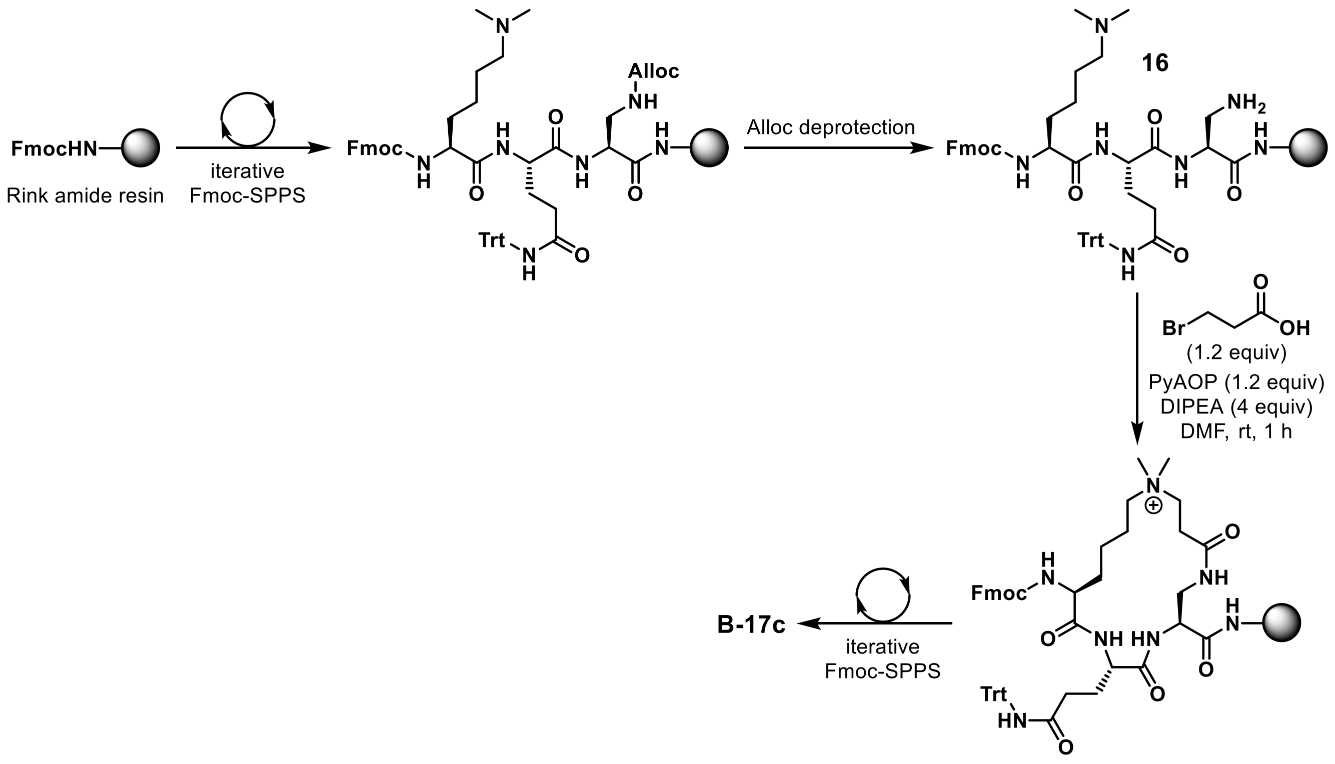

Tripeptide Fmoc-Lys(me2)-Gln(Trt)-Dap(Alloc)-CONH-Rink amide resin (1 equiv) was prepared on Rink amide resin according to the procedures outlined in the **Iterative Fmoc-SPPS workflow**. The Alloc group was deprotected (see **Deprotection procedures**) and the resin-bound tripeptide intermediate **16** was treated with a solution of 3-bromopropionic acid (1.2 equiv), PyAOP (1.2 equiv) and DIPEA (2.4 equiv) in DMF and agitated at room temperature for one hour, at which time consumption of starting material was observed by LCMS analysis. The resin-bound peptide was subsequently elongated following the **Iterative Fmoc-SPPS workflow** to afford lactam **B-17c**.

Compound **B-17c**

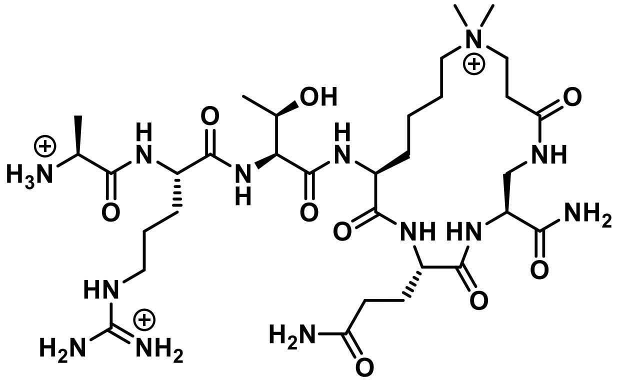

Yield: 8%

Exact mass: 772.48 $[C_{32}H_{62}N_{13}O_9]^{3+}$

HPLC trace (Method 1):

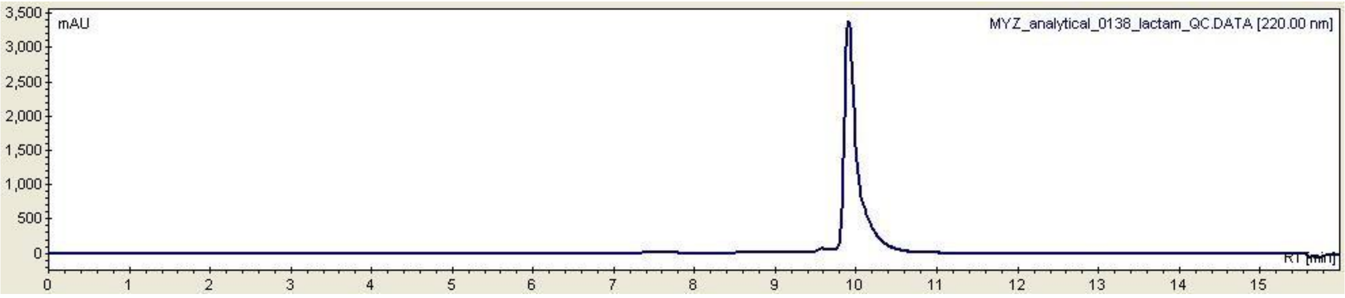

LCMS

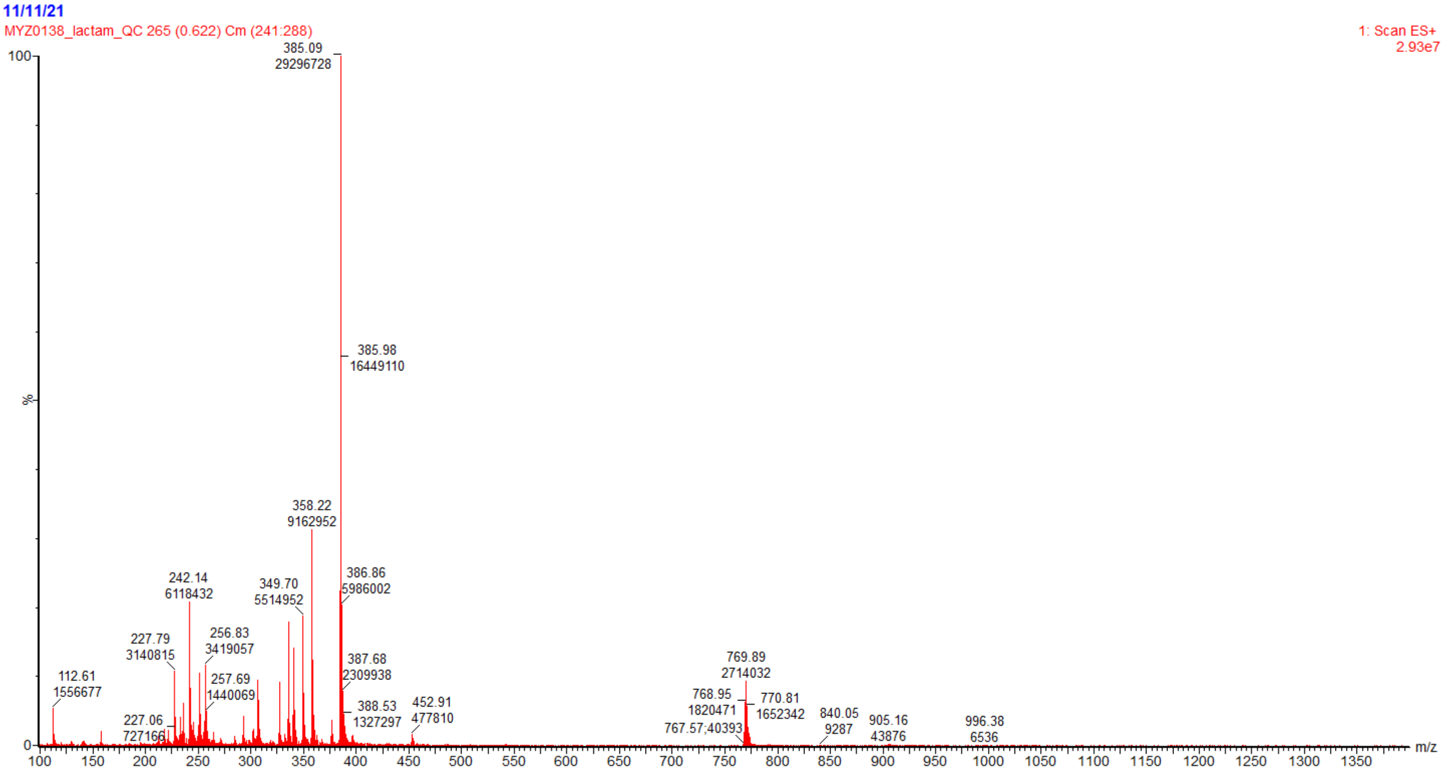

MALDI

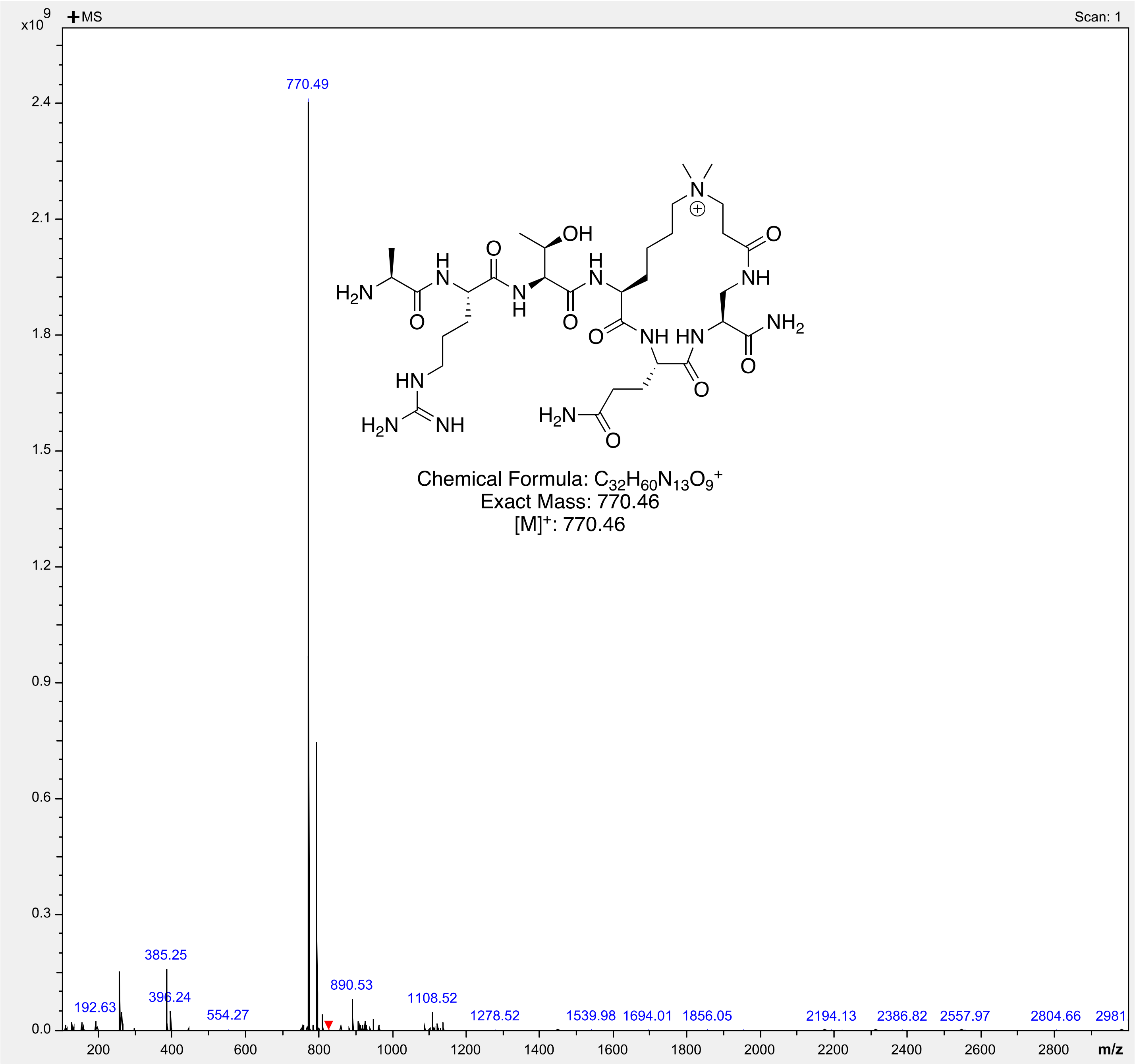

x10^9
+MS
Scan: 1
770.49
385.25
396.24
192.63
554.27
890.53
1108.52
1278.52
1539.98
1694.01
1856.05
2194.13
2386.82
2557.97
2804.66
2981
m/z
Chemical Formula: $C_{32}H_{60}N_{13}O_9^+$
Exact Mass: 770.46
$[M]^+$: 770.46

Series C: thioether macrocyclization

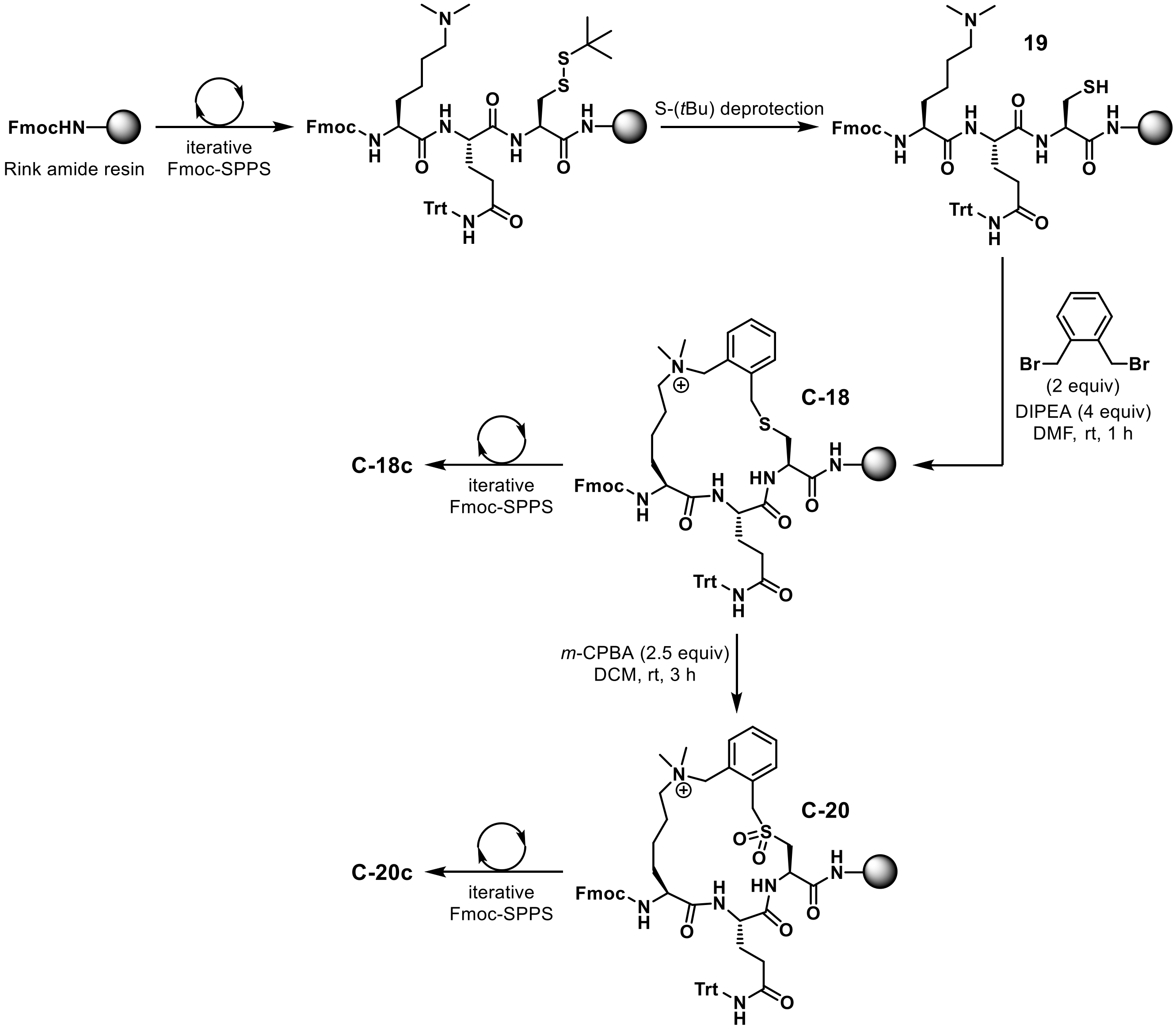

Tripeptide Fmoc-Lys(me2)-Gln(Trt)-Cys(S*t*Bu)-CONH-rink amide resin (1 equiv) was prepared on Rink amide resin according to the procedures outlined in the **Iterative Fmoc-SPPS workflow**. The resin was washed with DMF (5 × 3 mL) and the S-(*t*Bu) group was deprotected (see **Deprotection procedures**). The resin-bound tripeptide intermediate **19** was washed with DMF (5 × 3 mL) and treated with a solution of DIPEA (4 equiv) in DMF, followed by a solution of 1,2-bis(bromomethyl)benzene (2 equiv) in DMF. The resin was agitated at room temperature for one hour, whilst monitoring the reaction using LCMS analysis. Once the formation of **C-18** and consumption of starting material was confirmed, the resin was washed with DMF (5 × 3 mL), DCM (5 × 3 mL) and DMF (5 × 3 mL), and the peptide was elongated following the **Iterative Fmoc-SPPS workflow** to afford thioether cyclopeptide **C-18c**.

Sulfone **C-20c** synthesis: Following the completion of the macrocyclization reaction using 1,2-bis(bromomethyl)benzene and DIPEA in DMF, the resin-bound thioether macrocycle **C-18** was washed with DMF (5 × 3 mL), DCM (5 × 3 mL), DMF (5 × 3 mL) and DCM (5 × 3 mL). The resin was subsequently treated with a solution of *m*-CPBA (2.5 equiv) in DCM and agitated at room temperature for three hours. Once complete oxidation of **C-18** to **C-20** was confirmed by LCMS, the liquid was discarded and the resin was washed with DMF (5 × 3 mL), DCM (5 × 3 mL) and DMF (5 × 3 mL). The peptide was elongated following the **Iterative Fmoc-SPPS workflow** to afford sulfone cyclopeptide **C-20c**.

Compound **C-18c**

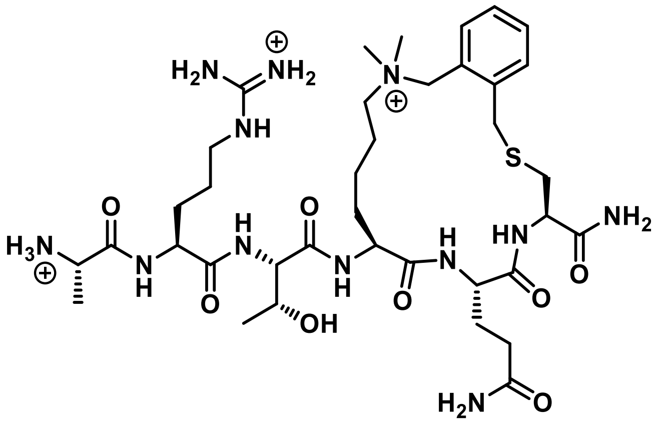

Yield: 10%

Exact mass: 837.48 $[C_{37}H_{65}N_{12}O_8S]^{3+}$

HPLC trace (Method 2):

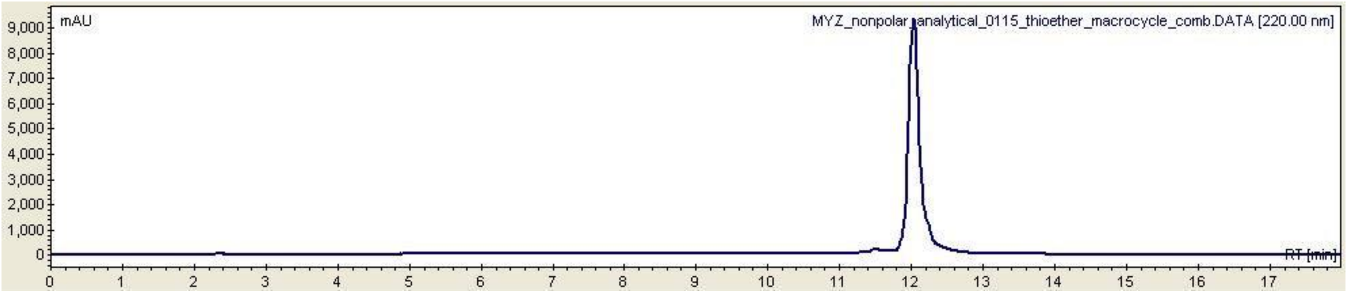

LCMS

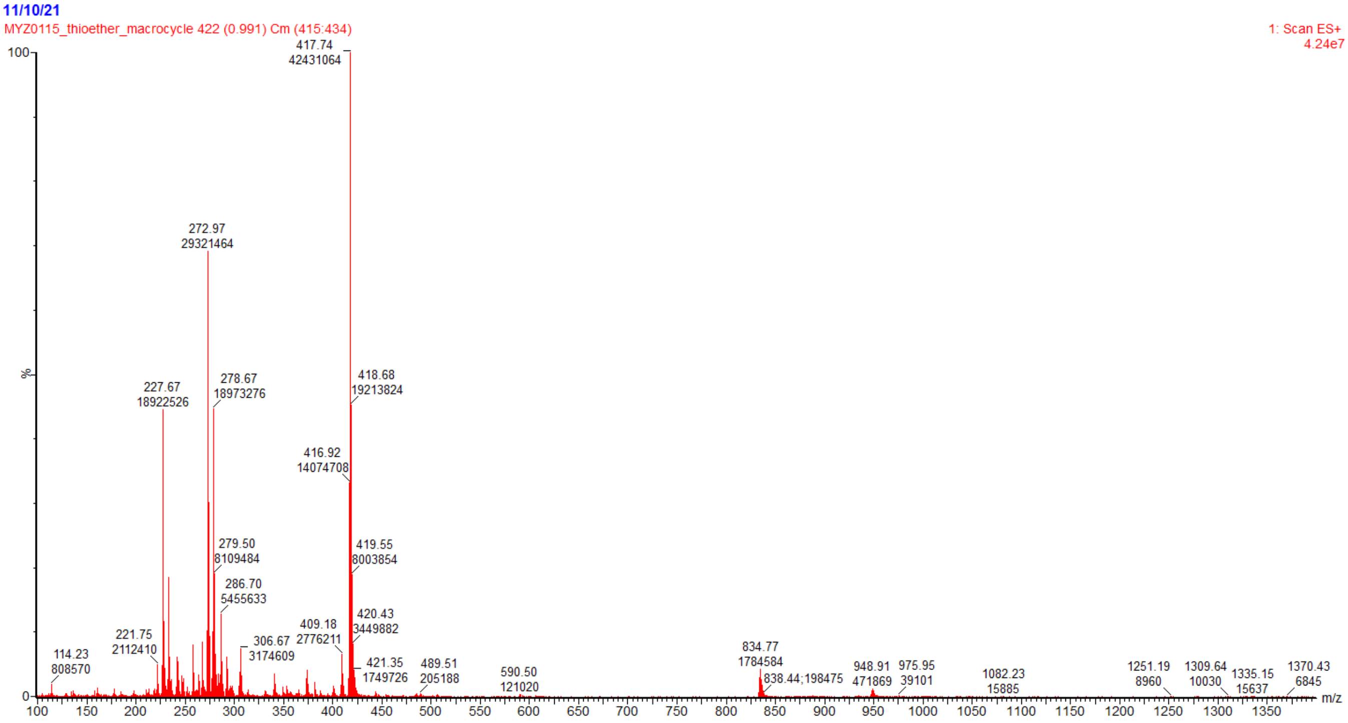

MALDI

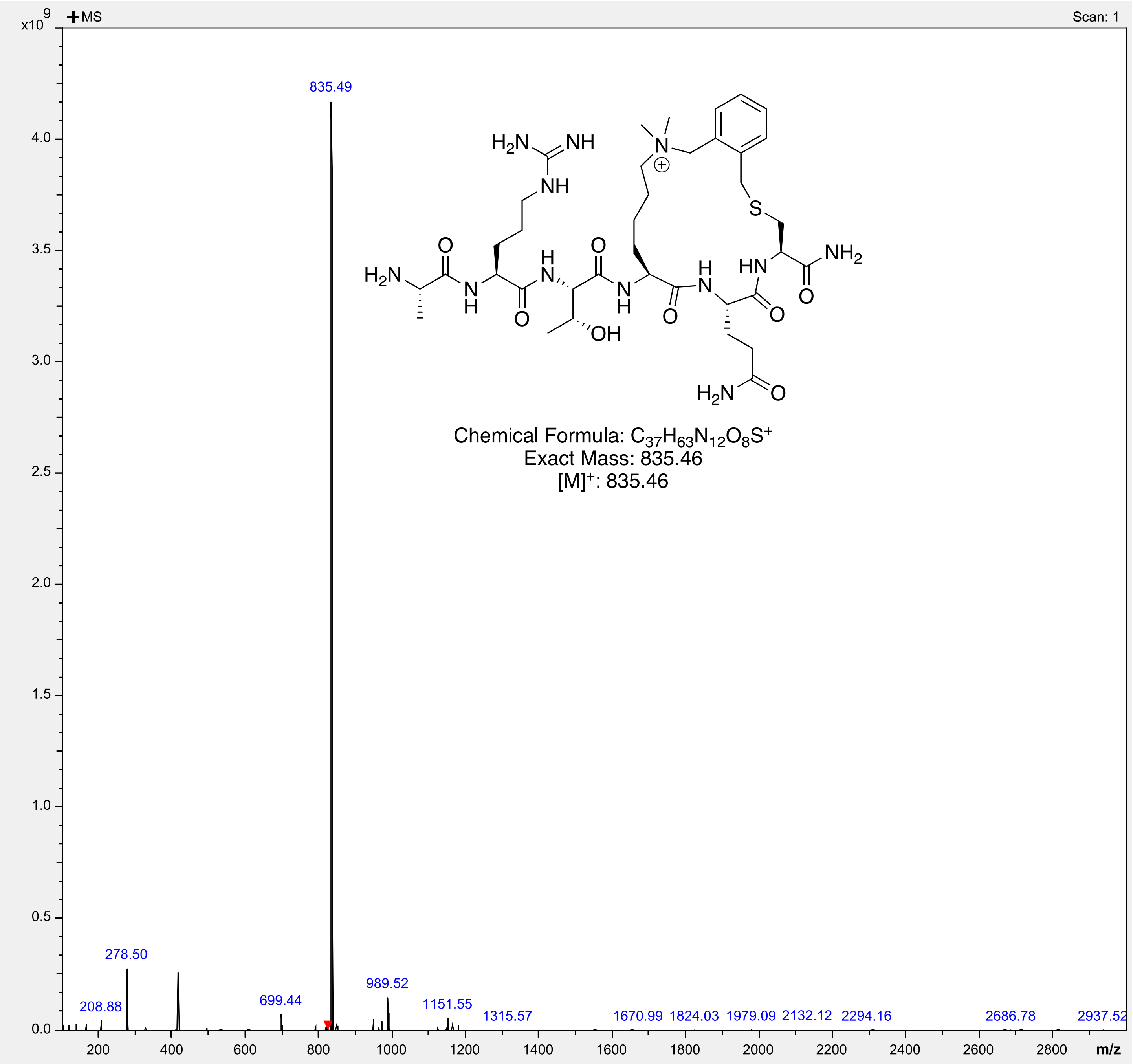

x10^9
+MS
Scan: 1
835.49
278.50
208.88
699.44
989.52
1151.55
1315.57
1670.99
1824.03
1979.09
2132.12
2294.16
2686.78
2937.52
m/z
Chemical Formula: C37H63N12O8S+
Exact Mass: 835.46
[M]+: 835.46

Compound **C-20c**

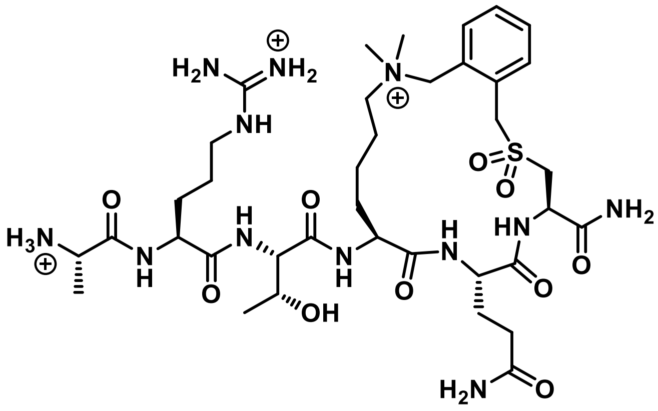

Yield: 1%

Exact mass: 869.47 $[C_{37}H_{65}N_{12}O_{10}S]^{3+}$

HPLC trace (<u>Method 2</u>):

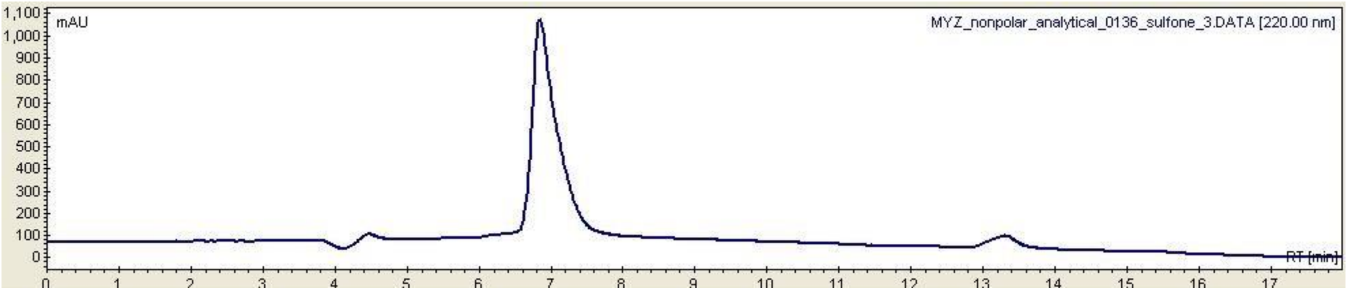

LCMS

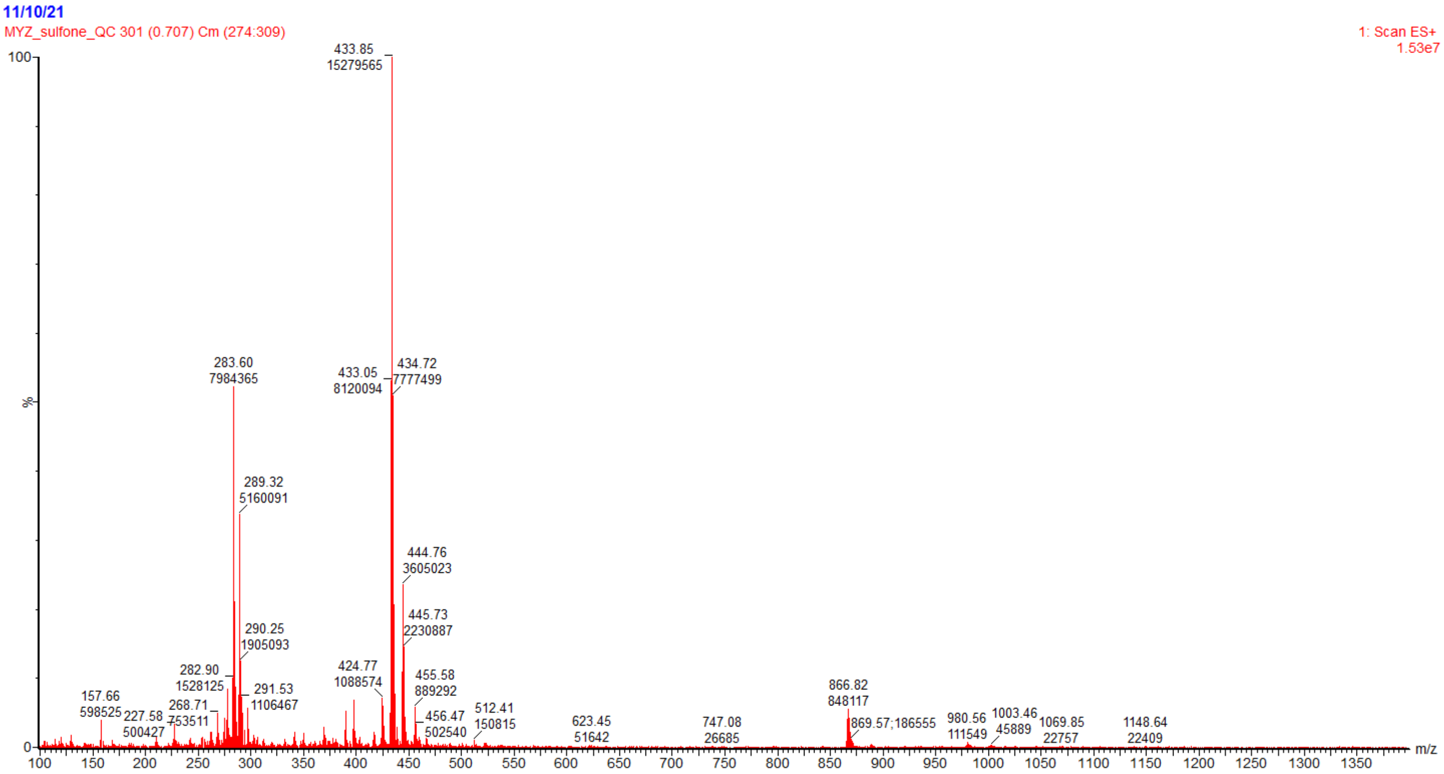

MALDI

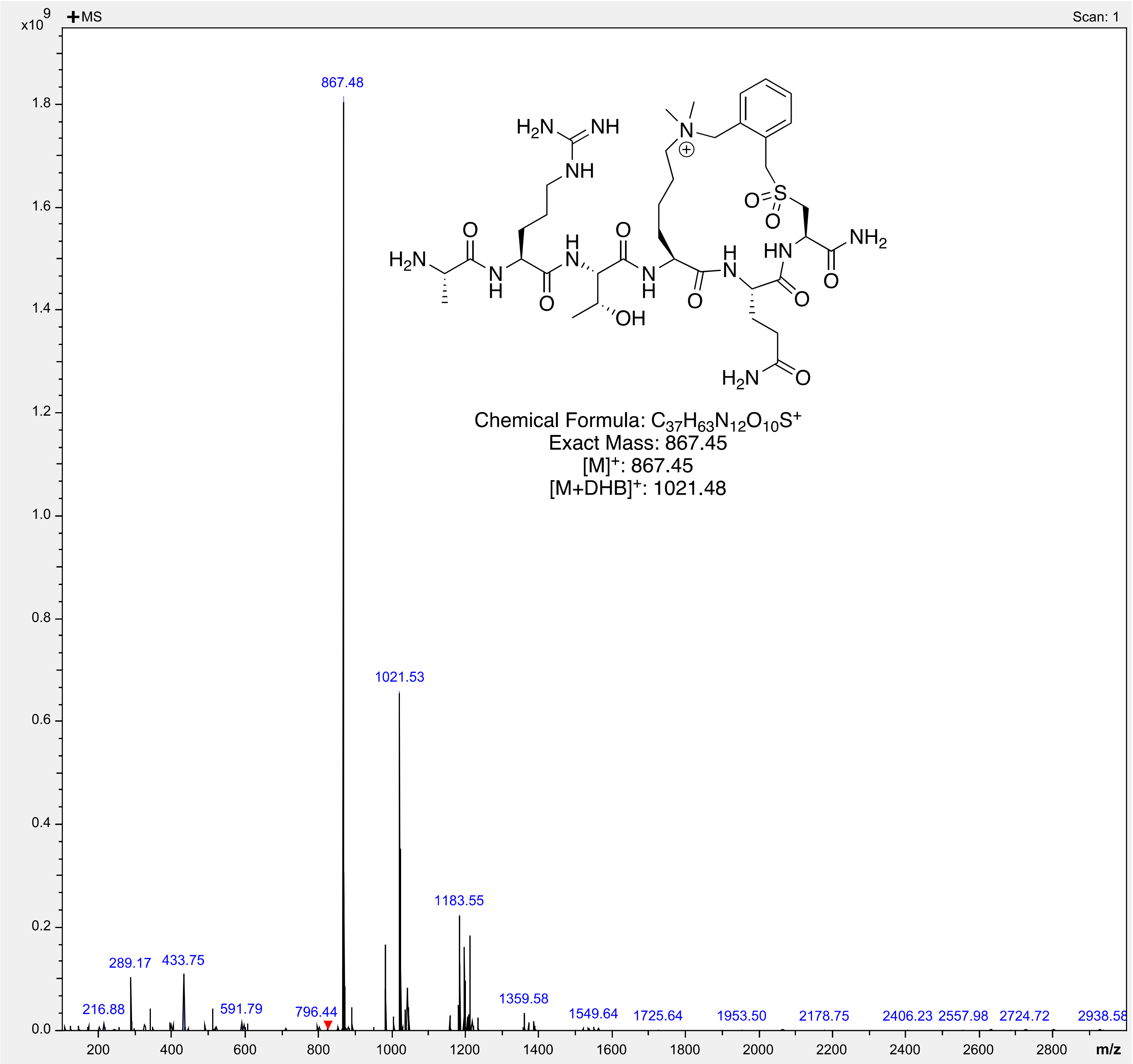
x10^9
+MS
Scan: 1
867.48
1021.53
1183.55
289.17
433.75
216.88
591.79
796.44
1359.58
1549.64
1725.64
1953.50
2178.75
2406.23
2557.98
2724.72
2938.58
m/z
Chemical Formula: $C_{37}H_{63}N_{12}O_{10}S^+$
Exact Mass: 867.45
$[M]^+$: 867.45
$[M+DHB]^+$: 1021.48

Series D: CuAAC reaction

Condition a:

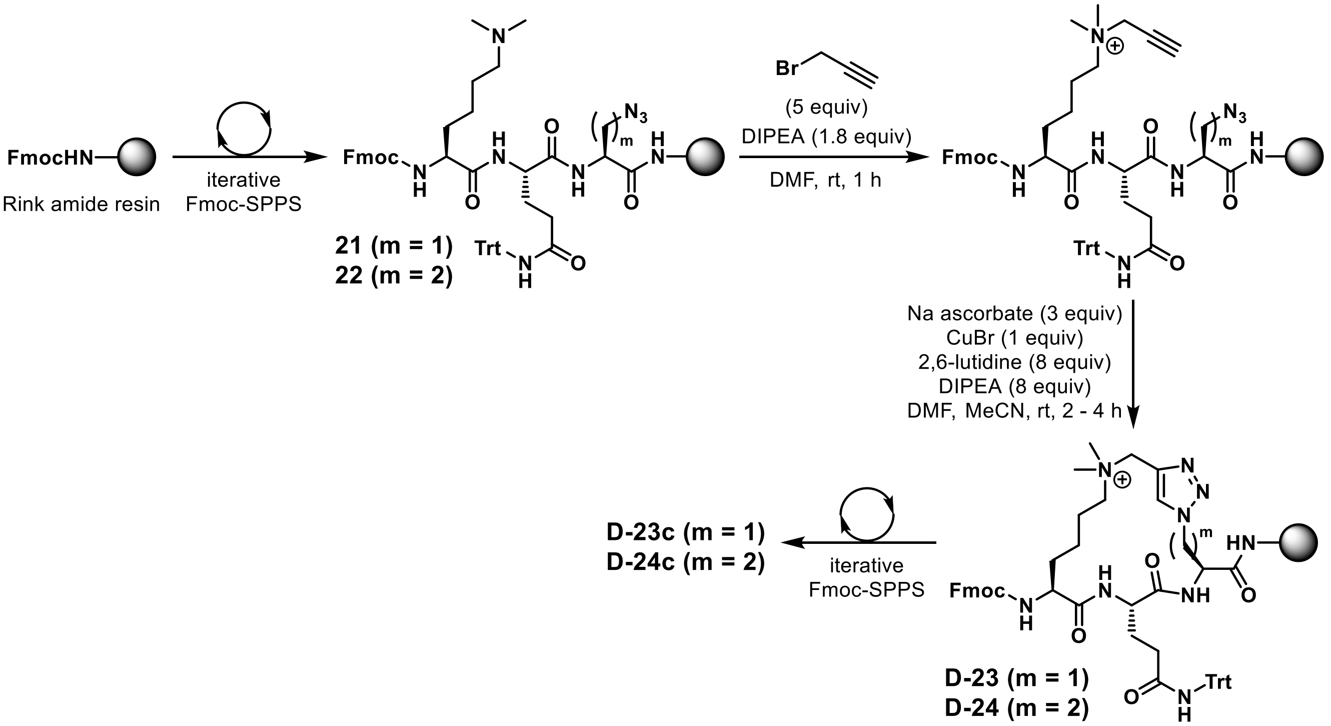

Tripeptide Fmoc-Lys(me2)-Gln(Trt)-Dap(N_3)-CONH-rink amide resin (**21**, 1 equiv) or Fmoc-Lys(me2)-Gln(Trt)-Dab(N_3)-CONH-rink amide resin (**22**, 1 equiv) was prepared on Rink amide resin according to the procedures outlined in the **Iterative Fmoc-SPPS workflow**. The resin was washed with DMF (5 × 3 mL) and treated with a solution of propargyl bromide (5 equiv) and DIPEA (1.8 equiv) in DMF at room temperature for one hour. The alkylation reaction was monitored by LCMS, and upon consumption of starting material the liquid was discarded, and the resin was washed with DMF (5 × 3 mL), DCM (5 × 3 mL) and DMF (5 × 3 mL).

The subsequent CuAAC reaction followed a modified literature procedure.[2] The resin was transferred to a Synthware™ cylindrical pressure vessel (15 mL O.D.) sealed with a rubber septum using 1 mL of DMF, and the reaction flask was degassed and refilled with nitrogen. 2,6-Lutidine (8 equiv) and DIPEA (8 equiv) were added to the resin followed by the addition of sodium ascorbate (3 equiv as a 1% solution in degassed DMF) and copper(I) bromide (1 equiv as a 1% solution in degassed acetonitrile) was added last. The resin mixture was stirred at room temperature under nitrogen for 2-4 hours whilst monitoring the reaction using analytical HPLC. Following consumption of starting material, the reaction was transferred to a plastic fritted syringe and washed with DMF (5 × 3 mL), DCM (5 × 3 mL) and DMF (5 × 3 mL). The cyclized tripeptide intermediates **D-23** and **D-24** were elongated following the procedures outlined in the **Iterative Fmoc-SPPS workflow** to afford triazoles **D-23c** and **D-24c**.

Compound **D-23c**

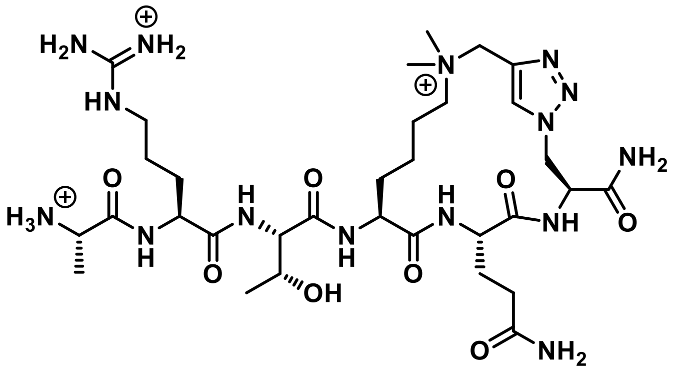

Yield: 2%

Exact mass: 782.47 $[C_{32}H_{60}N_{15}O_8]^{3+}$

HPLC trace (<u>Method 1</u>):

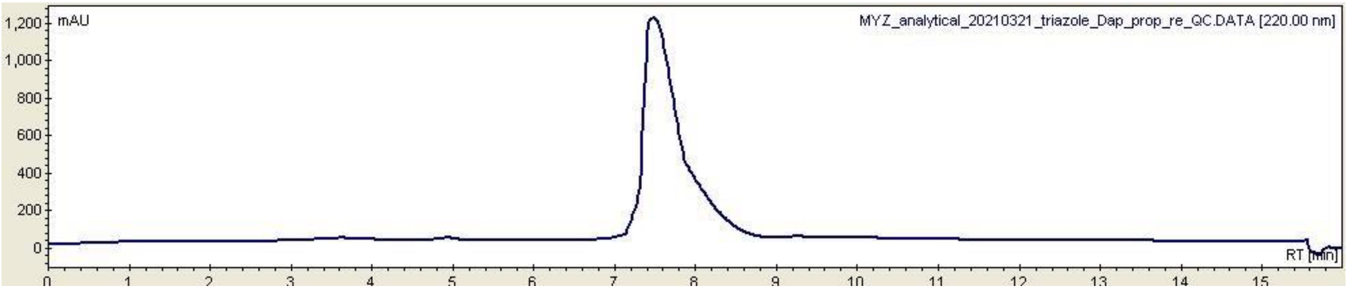

LCMS

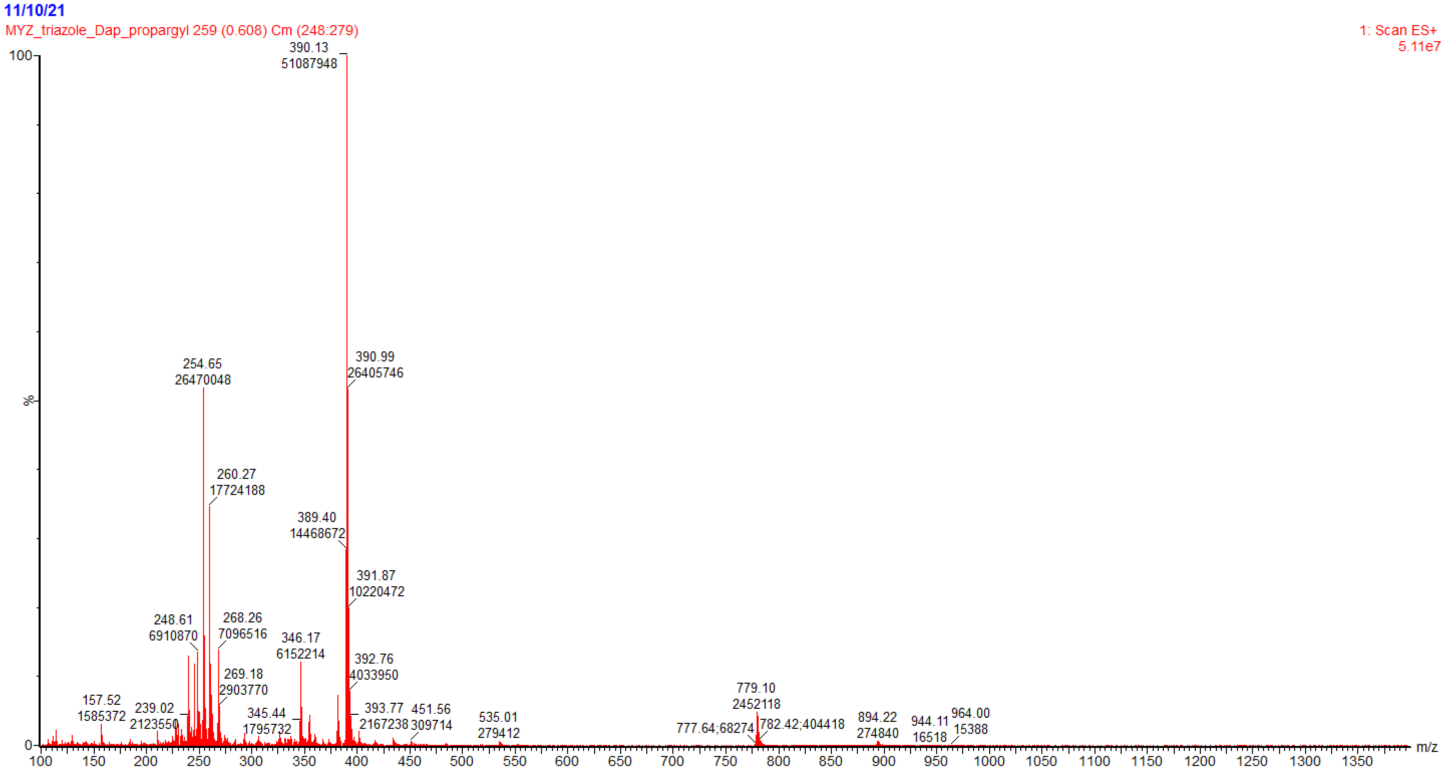

MALDI

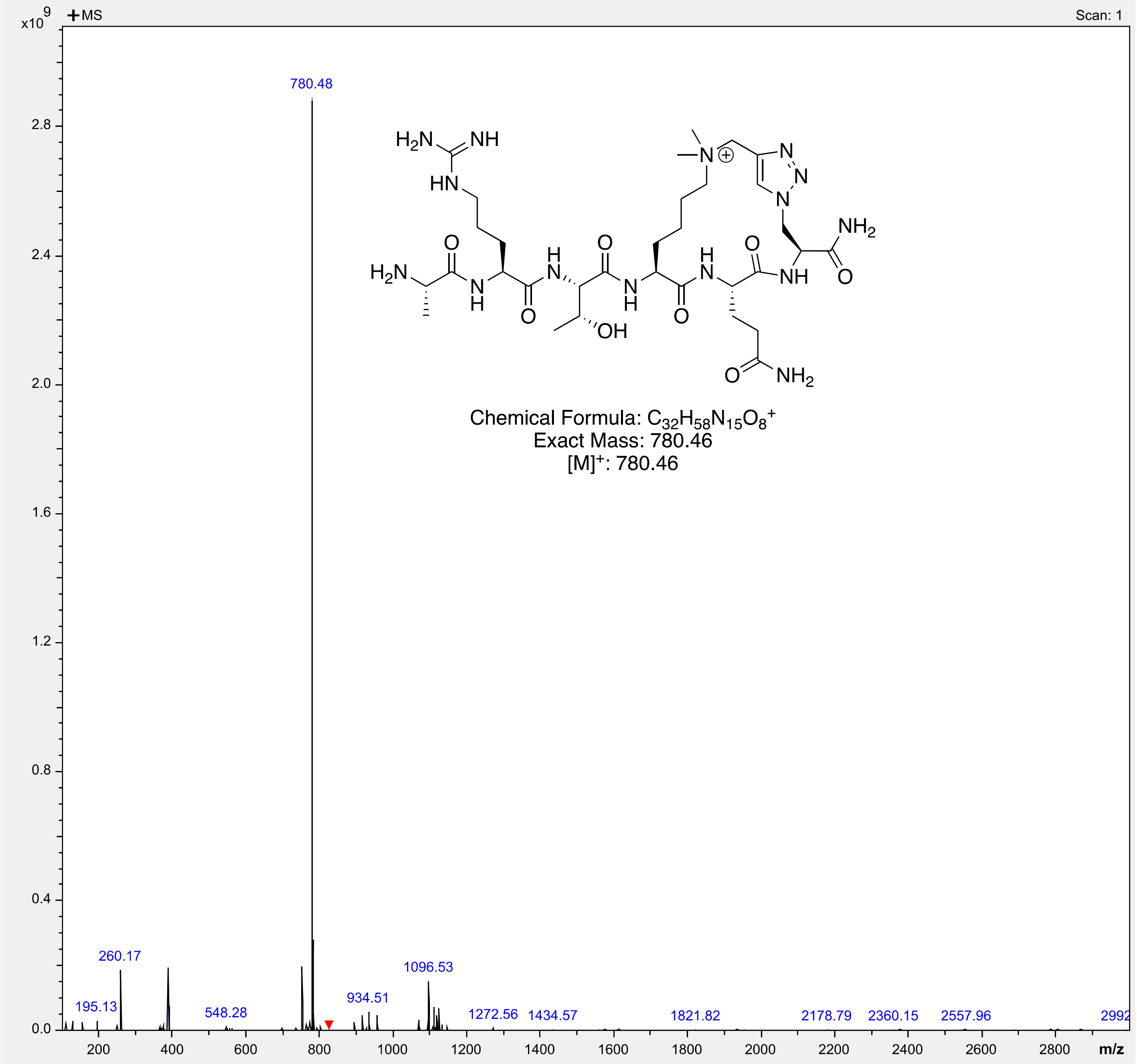

x10^9
+MS
Scan: 1
780.48
260.17
195.13
548.28
934.51
1096.53
1272.56
1434.57
1821.82
2178.79
2360.15
2557.96
2992
m/z
Chemical Formula: C32H58N15O8+
Exact Mass: 780.46
[M]+: 780.46

Compound **D-24c**

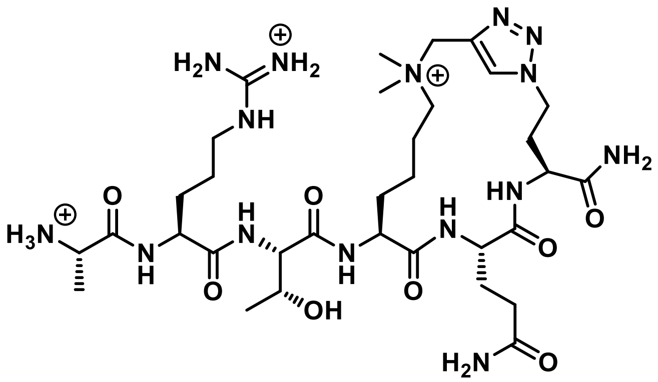

Yield: 5%

Exact mass: 796.49 $[C_{33}H_{62}N_{15}O_8]^{3+}$

HPLC trace (<u>Method 1</u>):

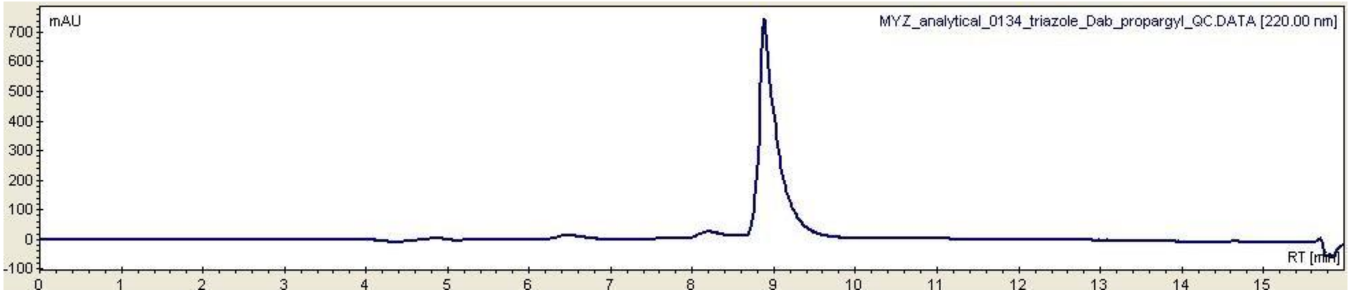

LCMS

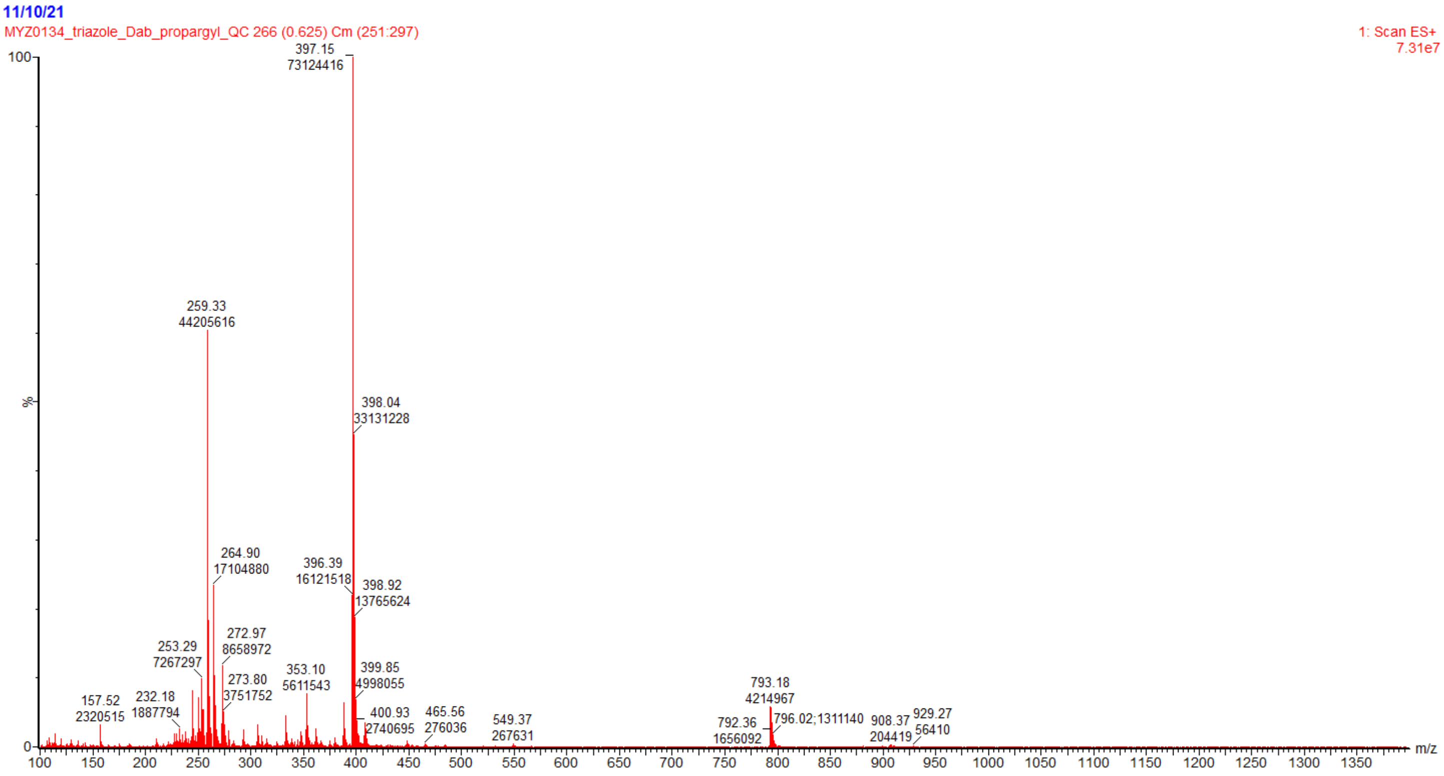

MALDI

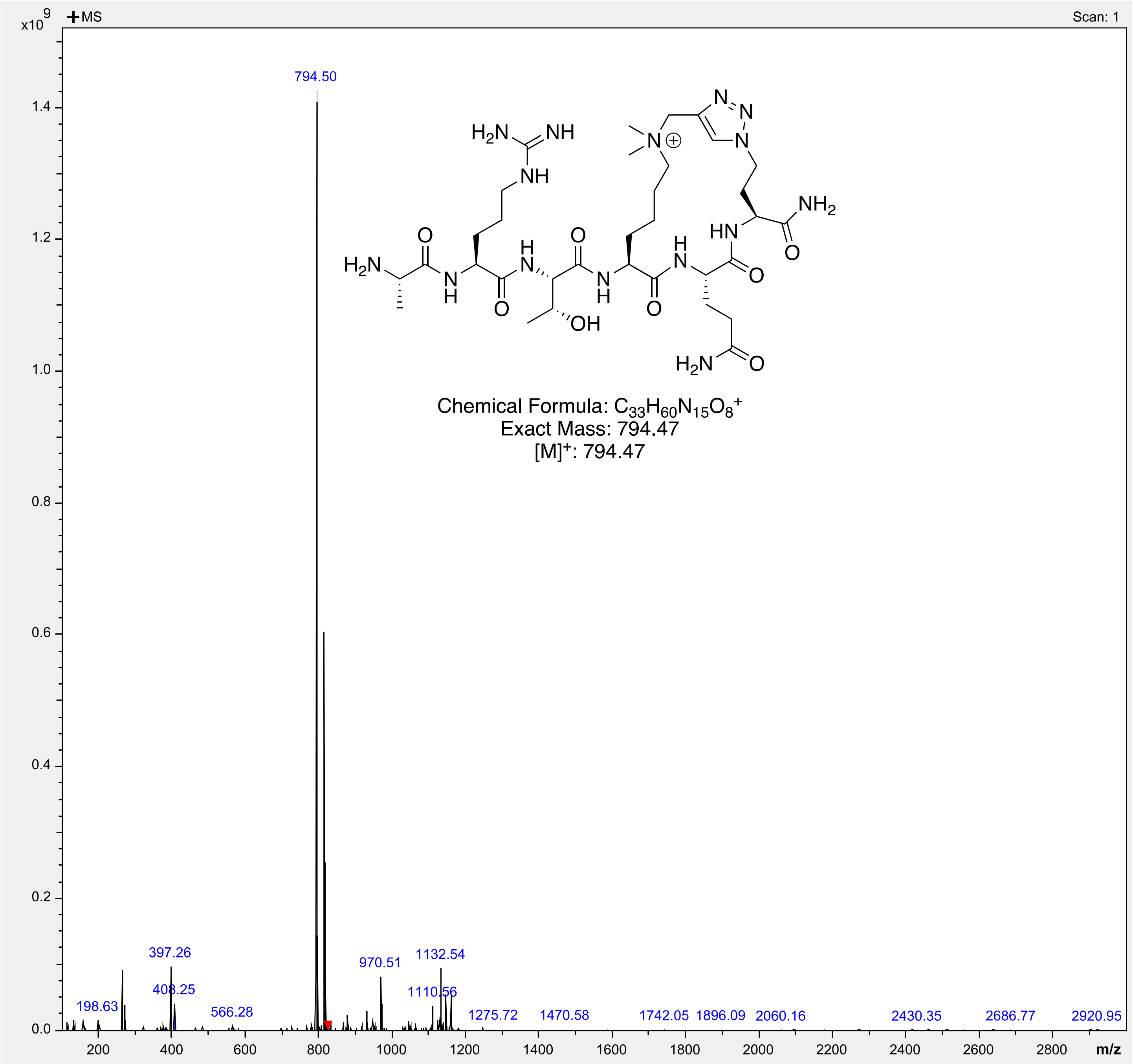
x10^9
+MS
Scan: 1
794.50
397.26
408.25
198.63
566.28
970.51
1132.54
1110.56
1275.72
1470.58
1742.05
1896.09
2060.16
2430.35
2686.77
2920.95
m/z
Chemical Formula: C33H60N15O8+
Exact Mass: 794.47
[M]+: 794.47

Condition b:

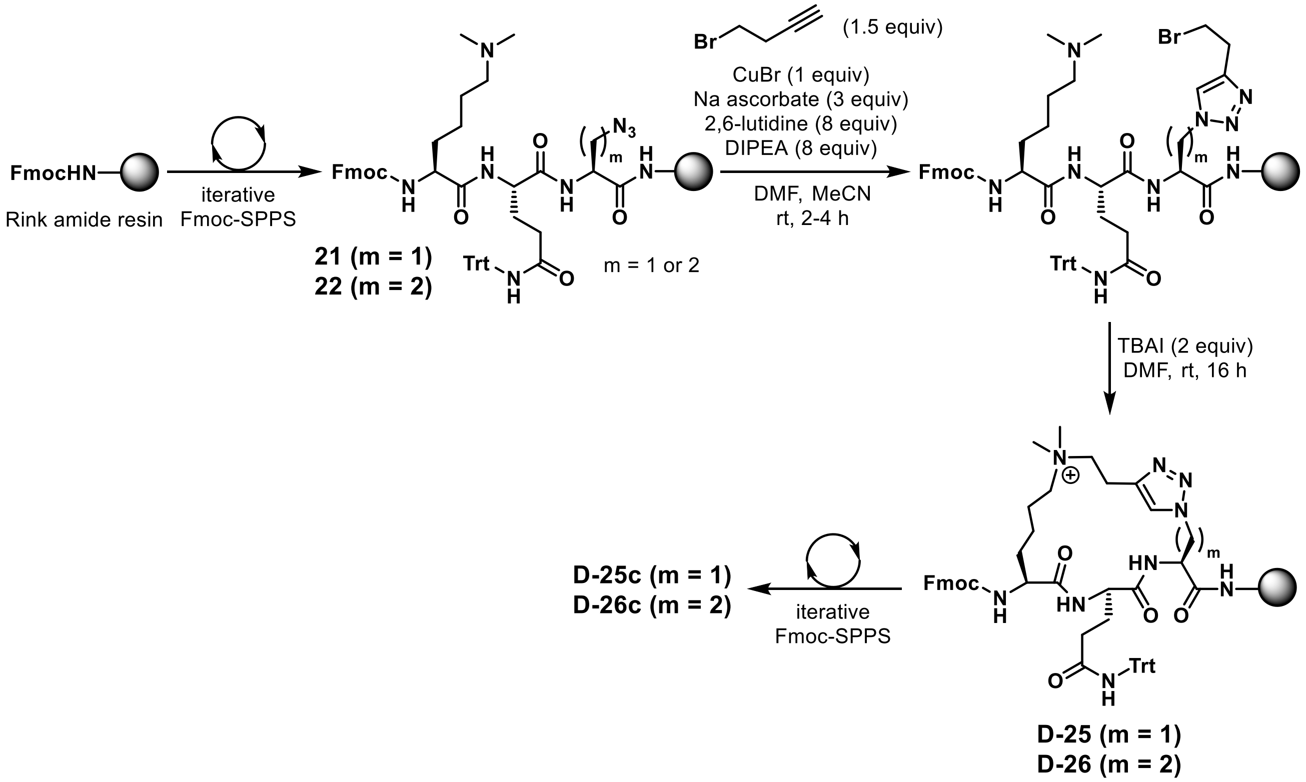

Tripeptide Fmoc-Lys(me2)-Gln(Trt)-Dap(N_3)-CONH-rink amide resin (**21**, 1 equiv) or Fmoc-Lys(me2)-Gln(Trt)-Dab(N_3)-CONH-rink amide resin (**22**, 1 equiv) was prepared on Rink amide resin according to the procedures outlined in the **Iterative Fmoc-SPPS workflow**. The resin was washed with DMF (5 × 3 mL).

The CuAAC reaction procedures from Condition a was used with the addition of 4-bromo-1-butyne (1.5 equiv). Following the consumption of starting material (monitored by LCMS analysis), the resin was transferred to a plastic fritted syringe and washed with DMF (5 × 3 mL), DCM (5 × 3 mL) and DMF (5 × 3 mL). The resin was treated with a solution of tetrabutylammonium iodide (2 equiv) in DMF and agitated at room temperature for 16 hours, at which time the liquid was expelled and the resin was washed with DMF (5 × 3 mL), DCM (5 × 3 mL) and DMF (5 × 3 mL). The cyclized tripeptide intermediates **D-25** and **D-26** were elongated following the **Iterative Fmoc-SPPS workflow** to afford triazoles **D-25c** and **D-26c**.

Compound **D-25c**

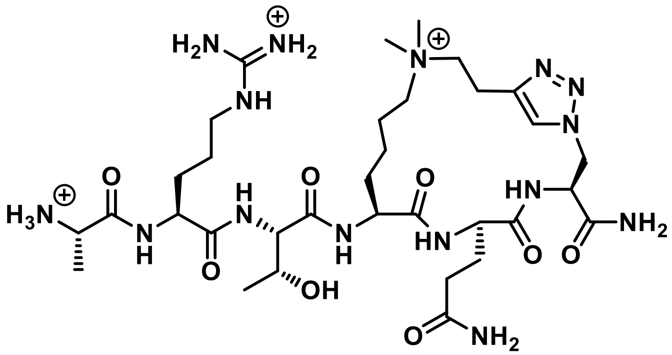

Yield: 2%

Exact mass: 796.49 $[C_{33}H_{62}N_{15}O_8]^{3+}$

HPLC trace (<u>Method 1</u>):

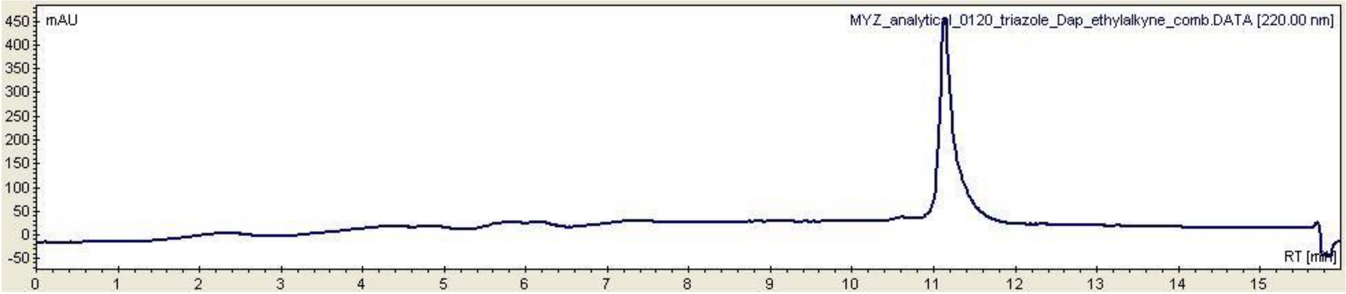

LCMS

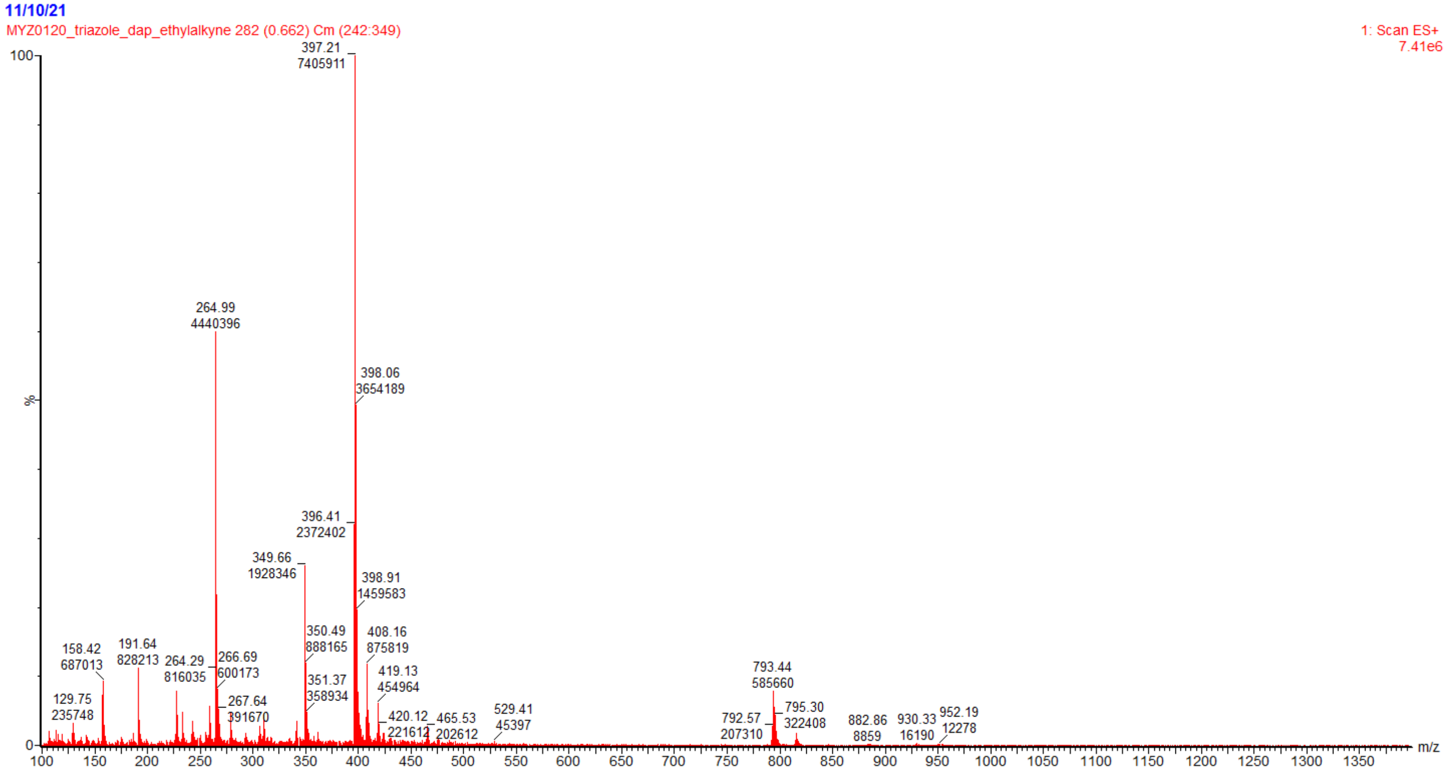

MALDI

%Int. 160 mV[sum= 14561 mV] Profiles 1-91 Smooth Sv-Gl 50 -Baseline 150

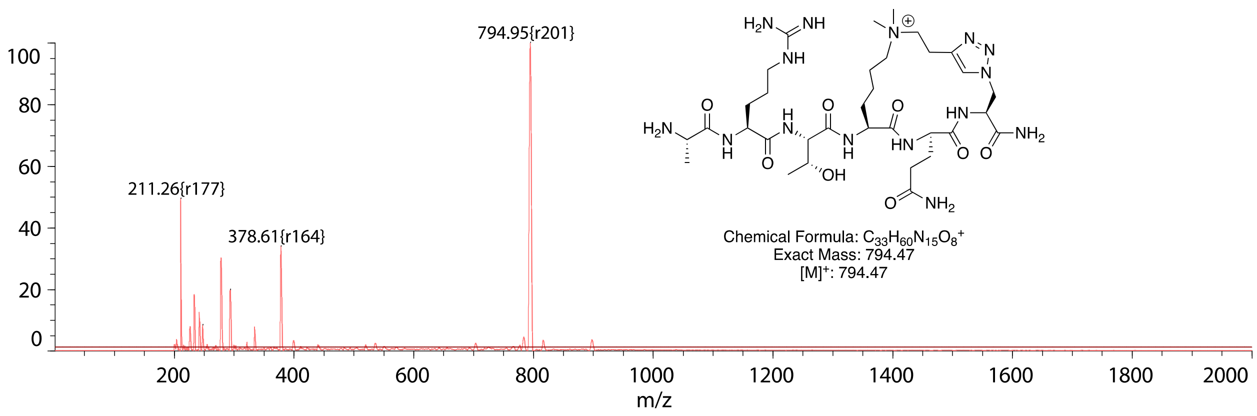

Compound **D-26c**

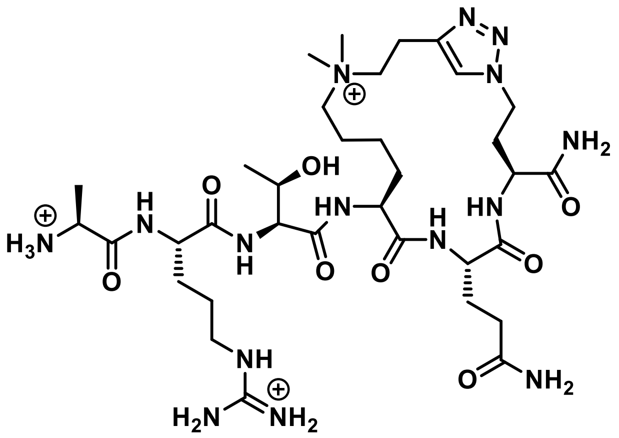

Yield: 2%

Exact mass: 810.50 $[C_{34}H_{64}N_{15}O_8]^{3+}$

HPLC trace (Method 1):

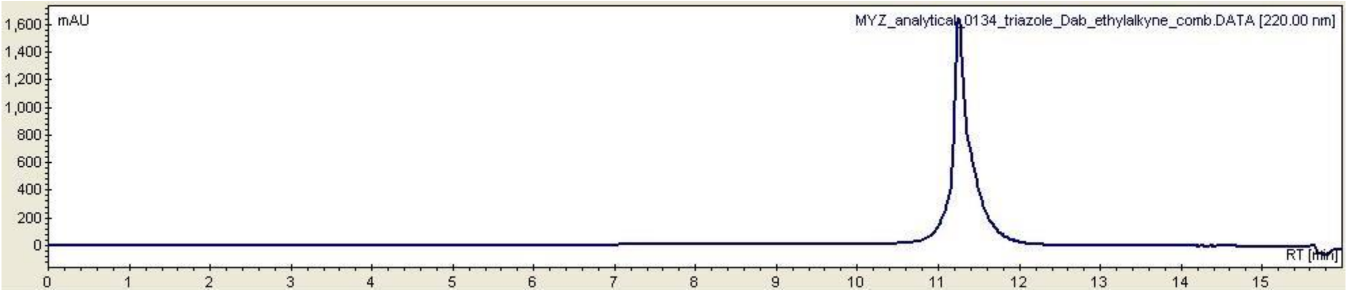

LCMS

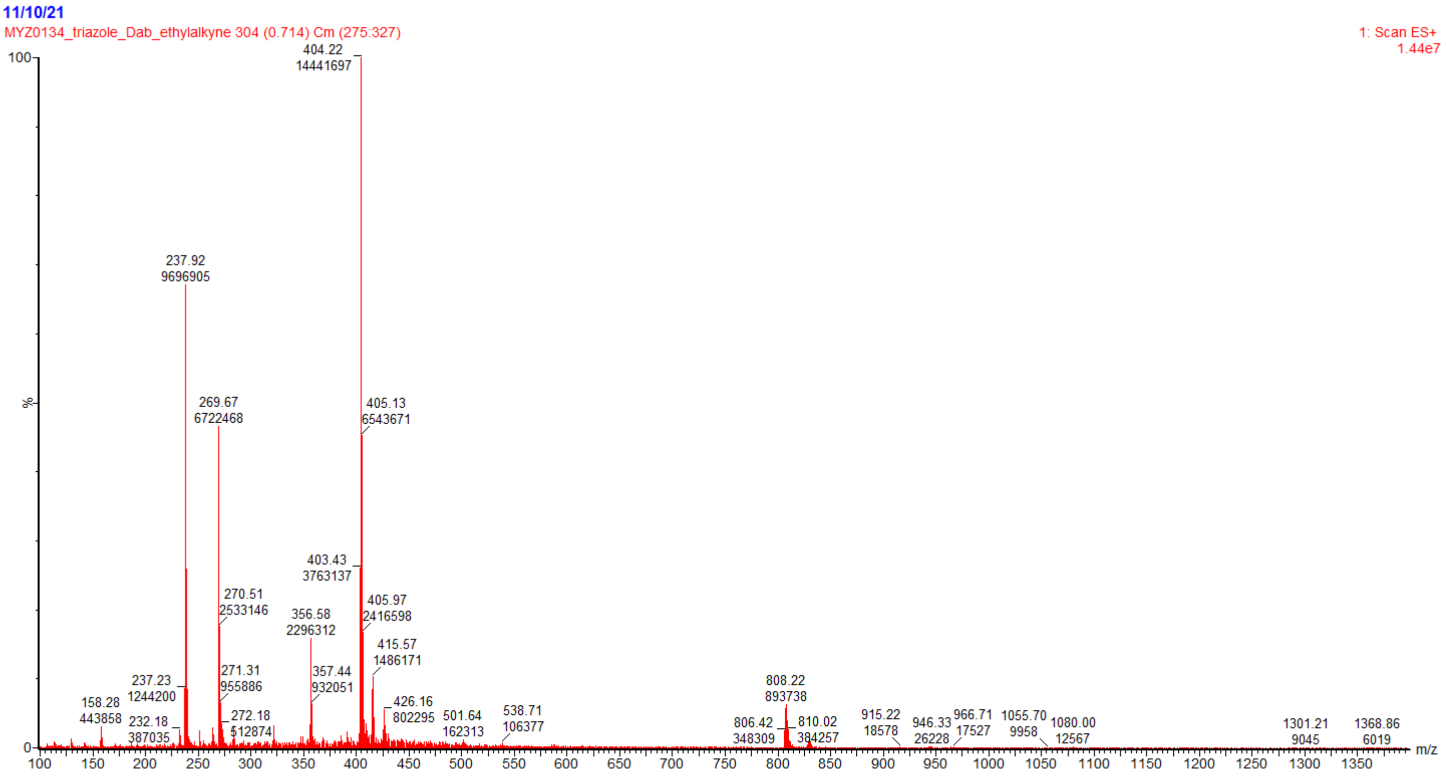

MALDI

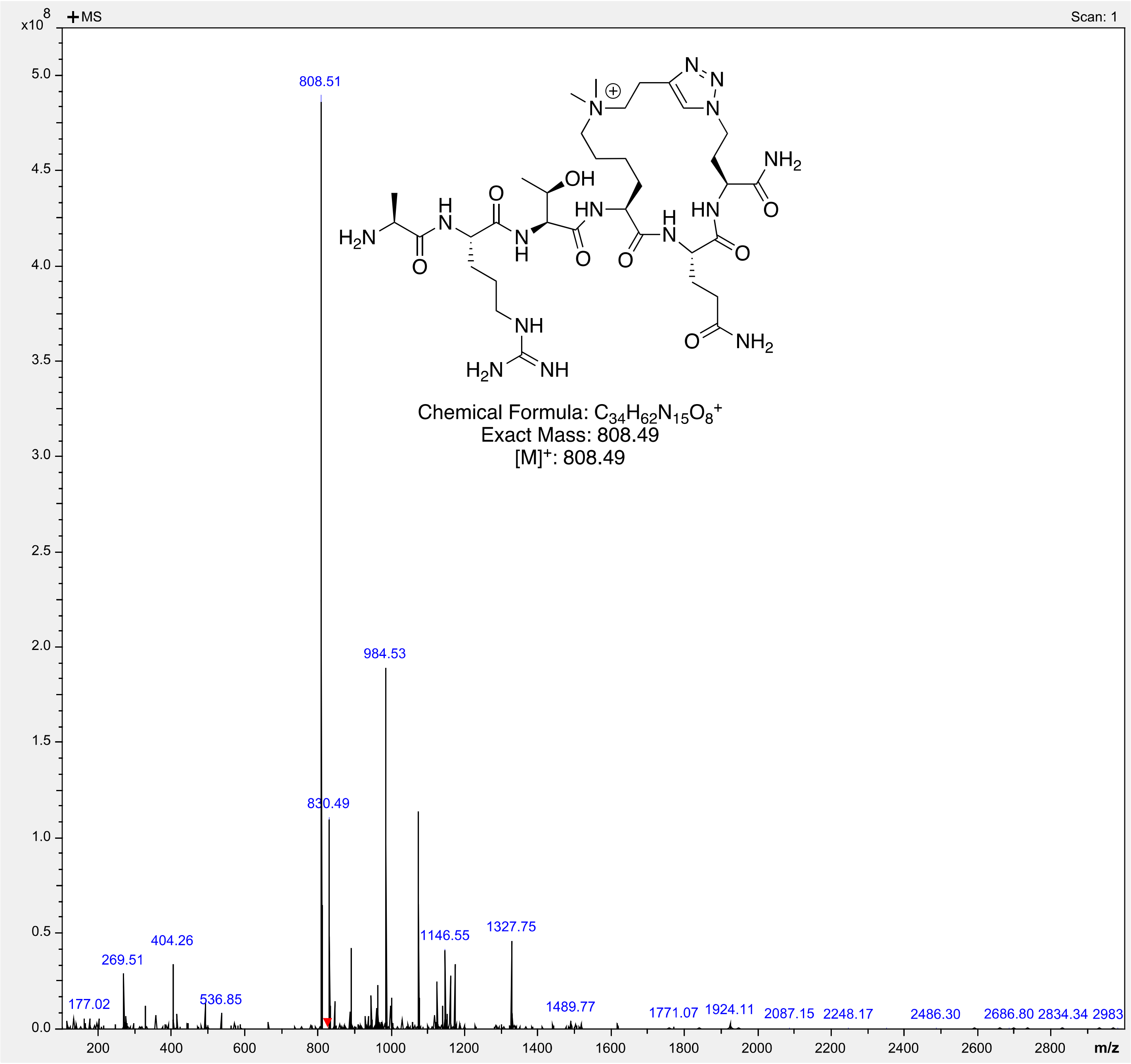

x10^8
+MS
Scan: 1
808.51
984.53
830.49
1146.55
1327.75
404.26
269.51
536.85
177.02
1489.77
1771.07
1924.11
2087.15
2248.17
2486.30
2686.80
2834.34
2983
m/z
Chemical Formula: C34H62N15O8+
Exact Mass: 808.49
[M]+: 808.49

Covalent Warhead Synthetic Procedures

Installation of the arylfluorosulfate covalent warhead using 4-(acetylamino)phenyl]imidodisulfuryl difluoride (AISF)

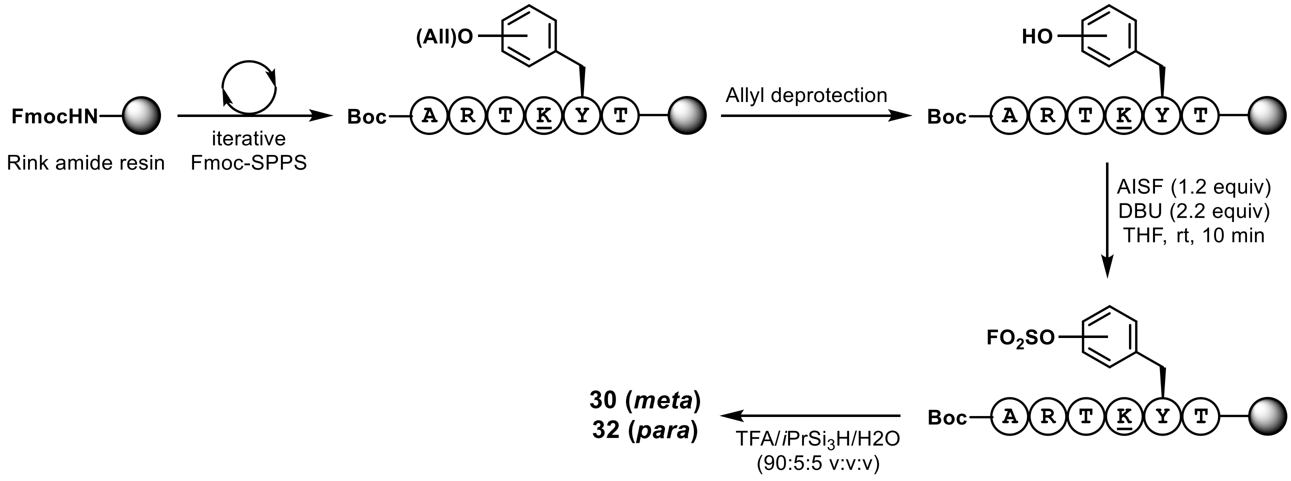

Hexapeptide Boc-Ala-Arg(Pbf)-Thr(*t*Bu)-K(me3)-Tyr(All)-Thr(*t*Bu)-CONH-Rink amide resin (1 equiv) was prepared on Rink amide resin according to the procedures outlined in the **Iterative Fmoc-SPPS workflow**. The resin was washed with DMF (5 × 3 mL), DCM (5 × 3 mL), DMF (5 × 3 mL) and DCM (5 × 3 mL). The Allyl group was deprotected according to the protocols outlined in the **Deprotection procedures**.

The arylfluorosulfate peptide synthesis followed a slightly modified literature procedure.[3] A solution of AISF (1.2 equiv) in THF was added to resin, followed by the addition of a solution of 1,8-diazabicyclo[5.4.0]undec-7-ene (2.2 equiv) in THF. The resin mixture was shaken at room temperature for 10 minutes at which time the liquid was expelled and the resin was washed with DMF (5 × 3 mL), DCM (5 × 3 mL), DMF (5 × 3 mL) and DCM (5 × 3 mL). Incorporation of the fluorosulfate functionality was confirmed by LCMS analysis. The peptide was cleaved from resin and purified according to the procedures outlined in the **Iterative Fmoc-SPPS workflow**.

Compound **32**

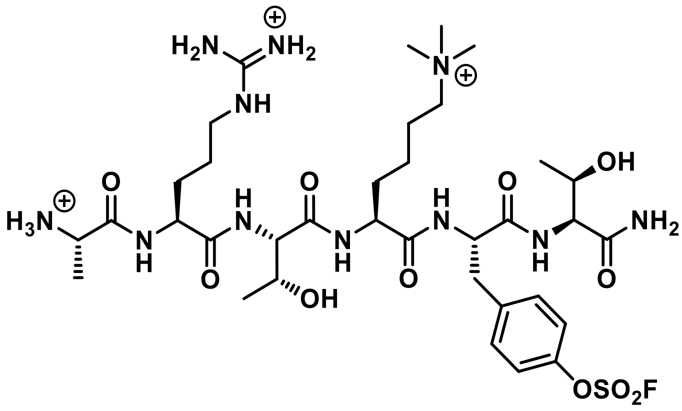

Yield: 11%

Exact mass: 864.44 $[C_{35}H_{63}FN_{11}O_{11}S]^{3+}$

HPLC trace (<u>Method 3</u>):

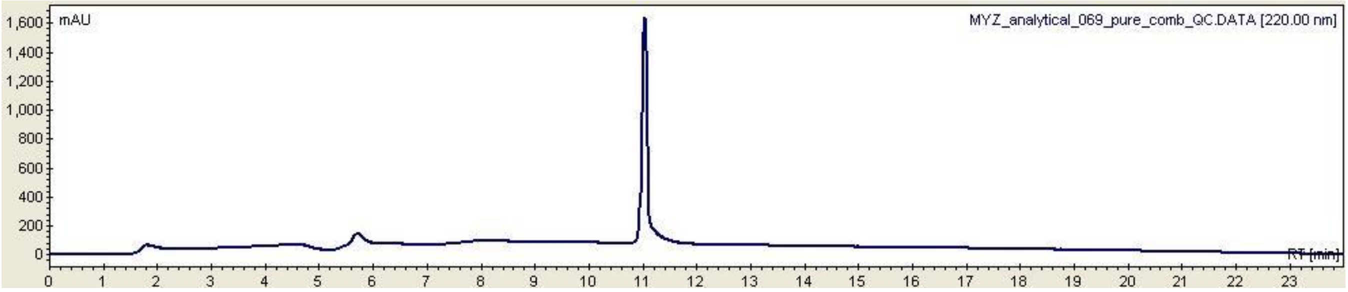

LCMS

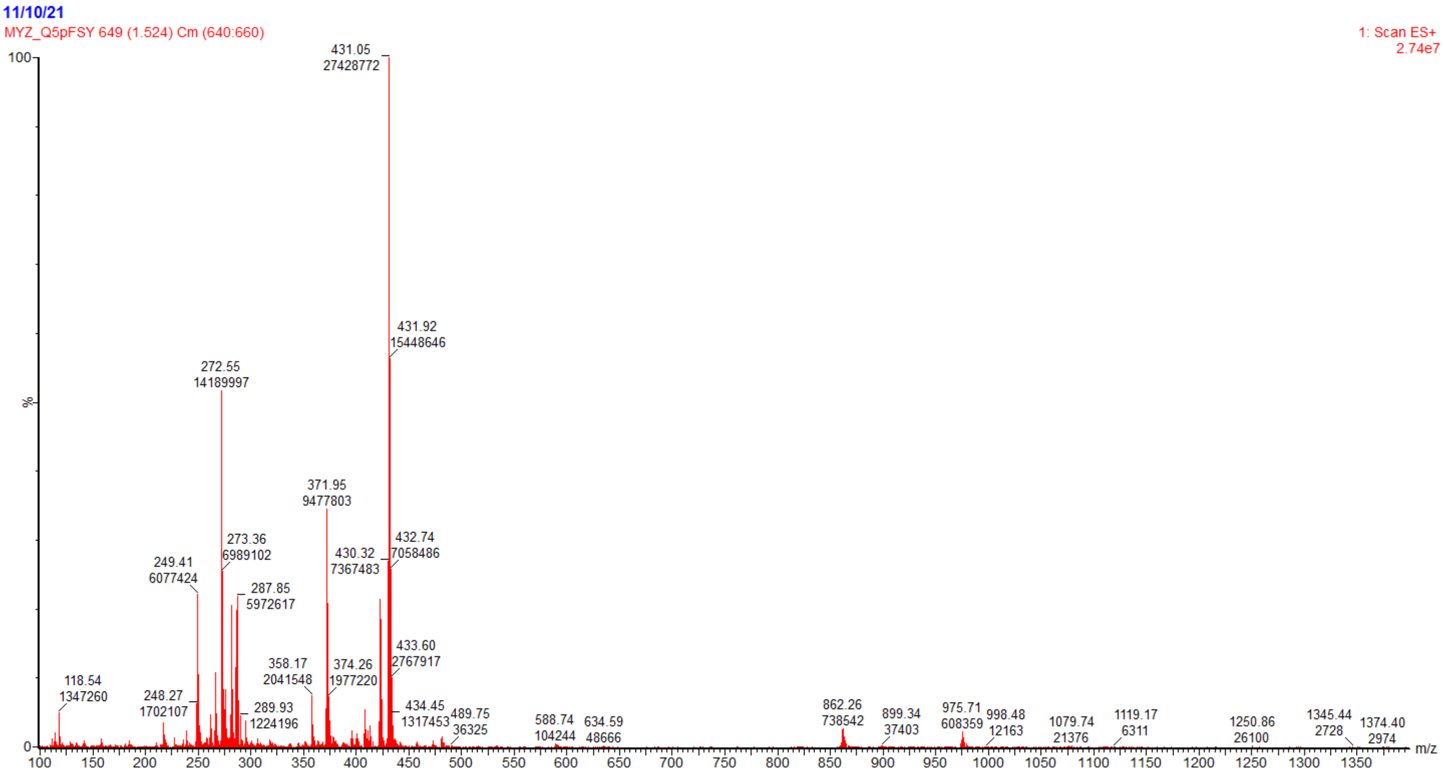

MALDI

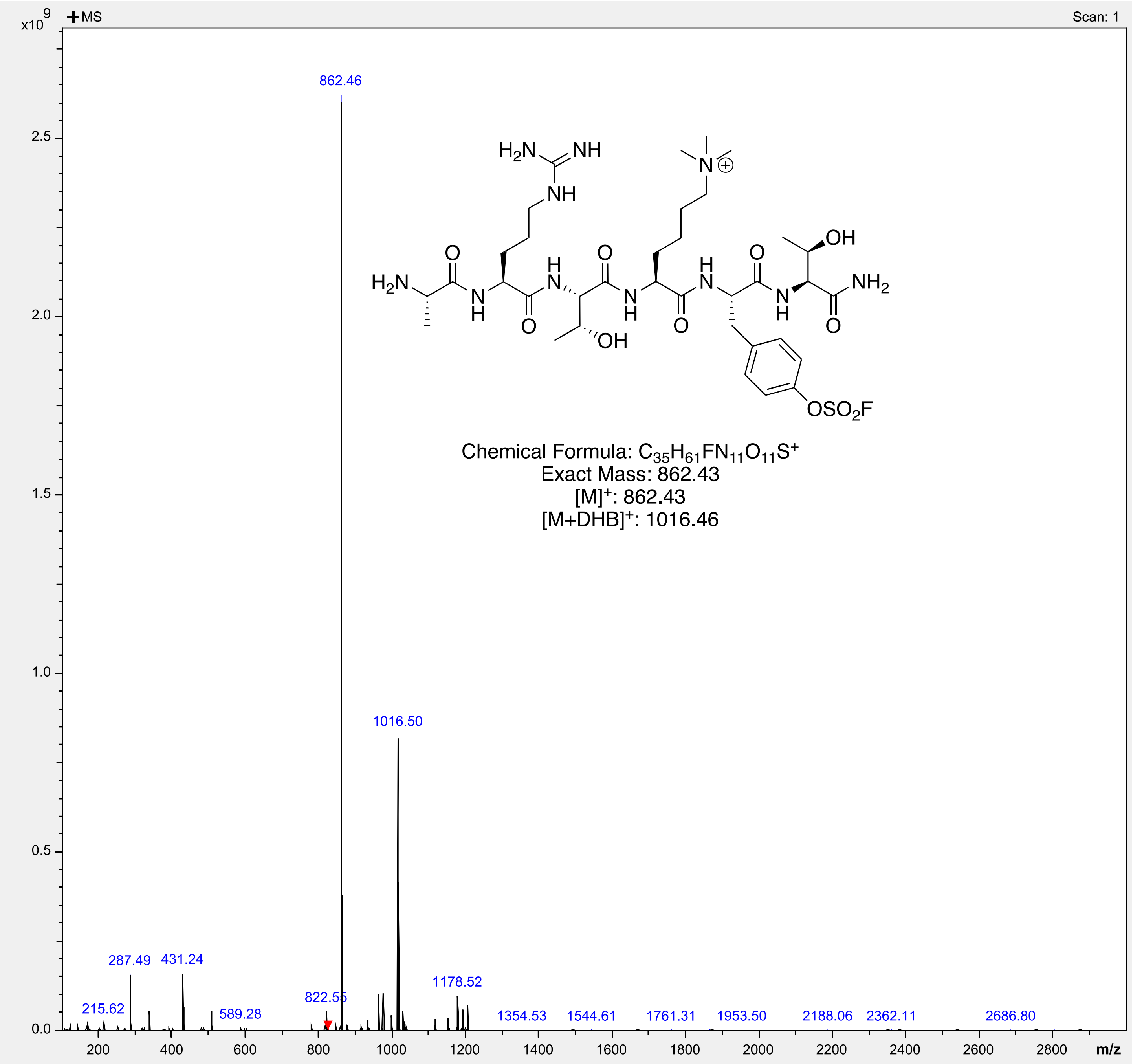

x10^9
+MS
Scan: 1
862.46
1016.50
287.49
431.24
215.62
589.28
822.55
1178.52
1354.53
1544.61
1761.31
1953.50
2188.06
2362.11
2686.80
m/z
Chemical Formula: C35H61FN11O11S+
Exact Mass: 862.43
[M]+: 862.43
[M+DHB]+: 1016.46

Compound **30**

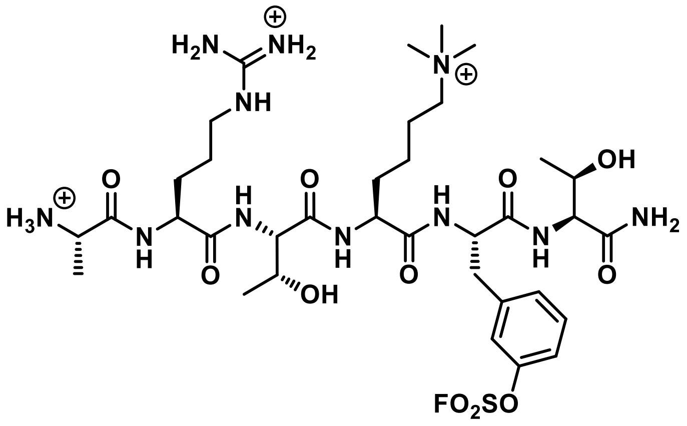

Yield: 27%

Exact mass: 864.44 $[C_{35}H_{63}FN_{11}O_{11}S]^{3+}$

HPLC trace (Method 2):

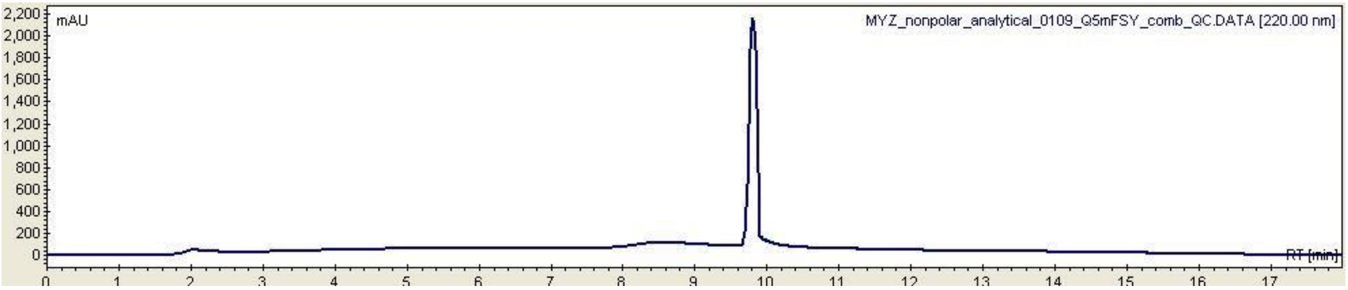

LCMS

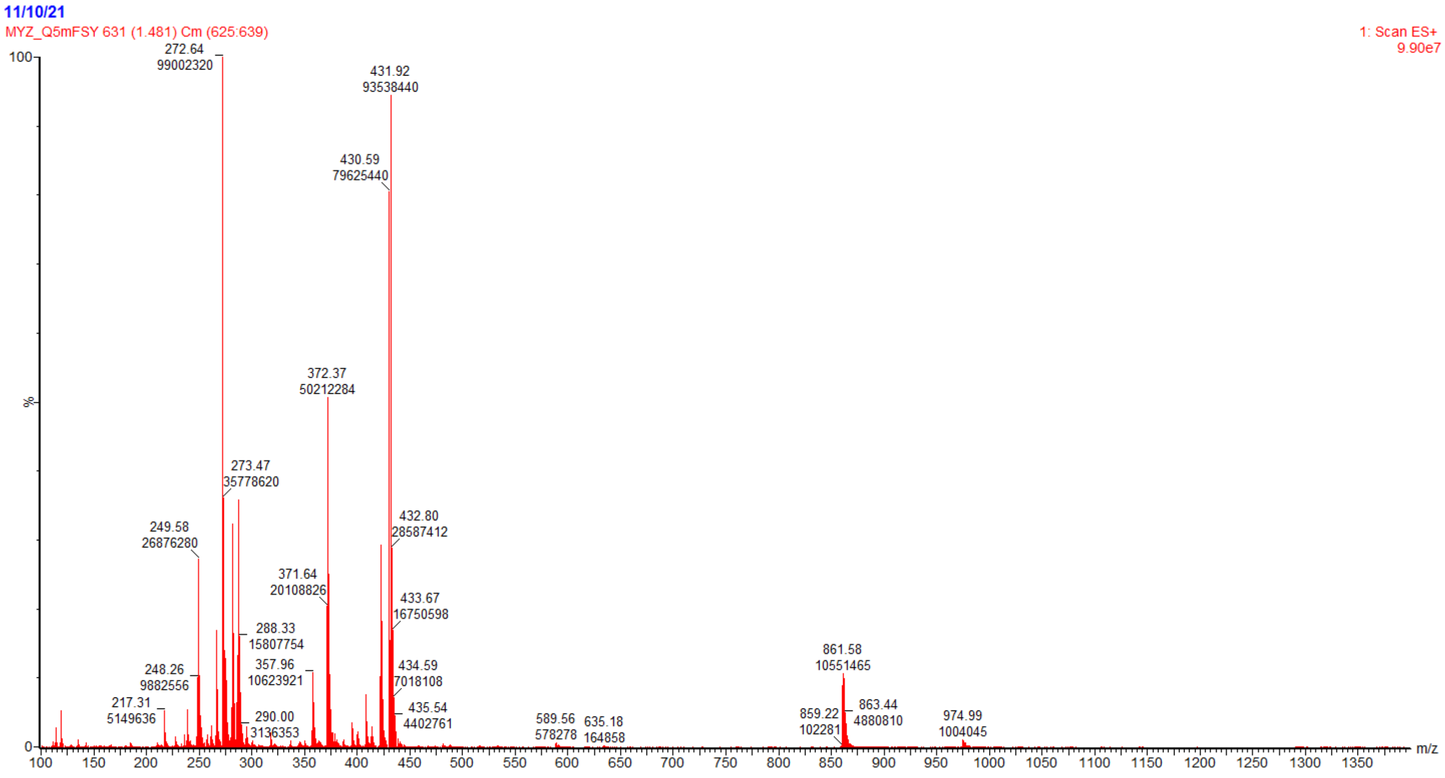

MALDI

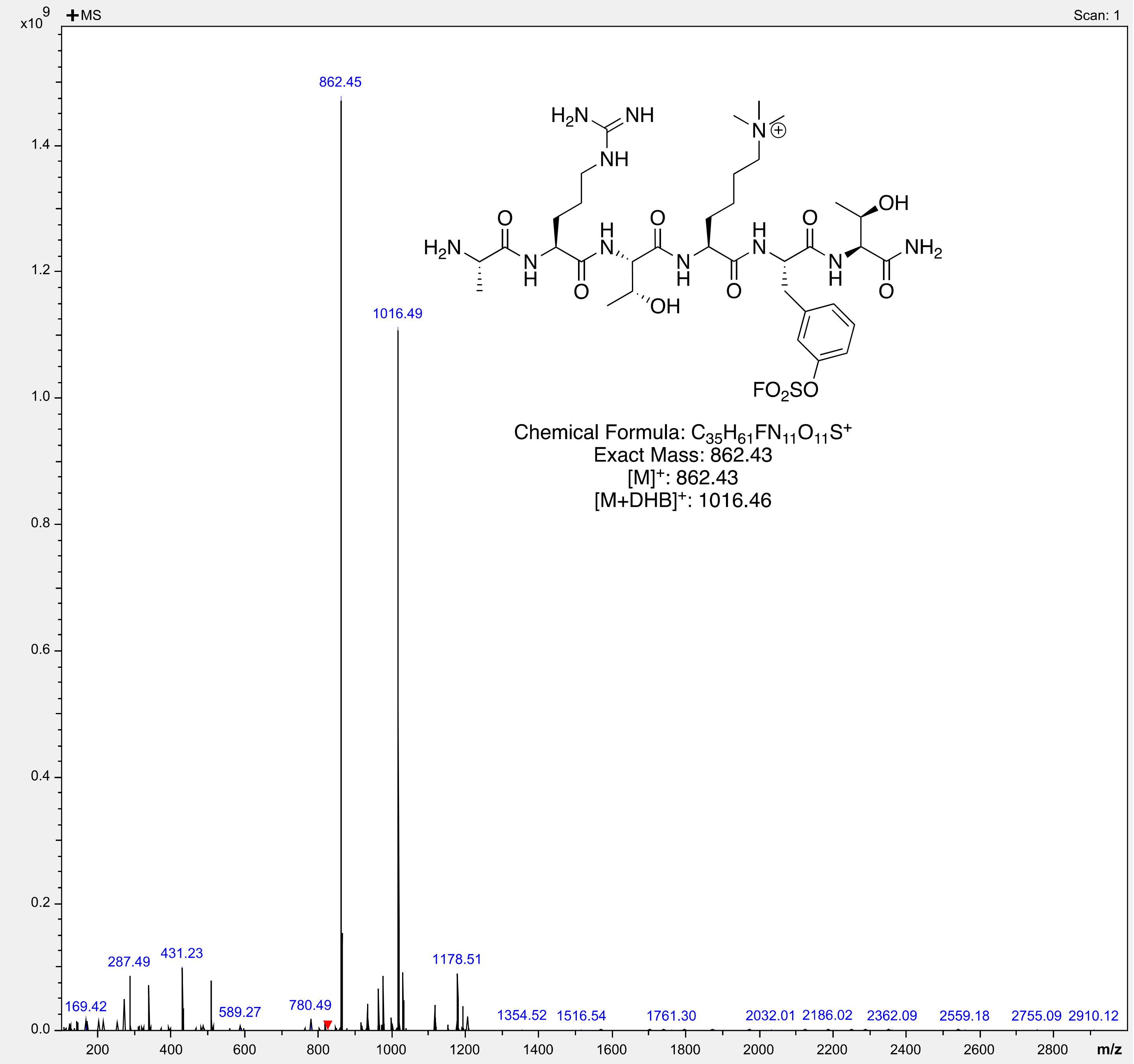

x10^9 +MS
Scan: 1
862.45
1016.49
1178.51
287.49
431.23
169.42
589.27
780.49
1354.52
1516.54
1761.30
2032.01
2186.02
2362.09
2559.18
2755.09
2910.12
m/z
H2N
NH
OH
NH2
FO2SO
Chemical Formula: C35H61FN11O11S+
Exact Mass: 862.43
[M]+: 862.43
[M+DHB]+: 1016.46

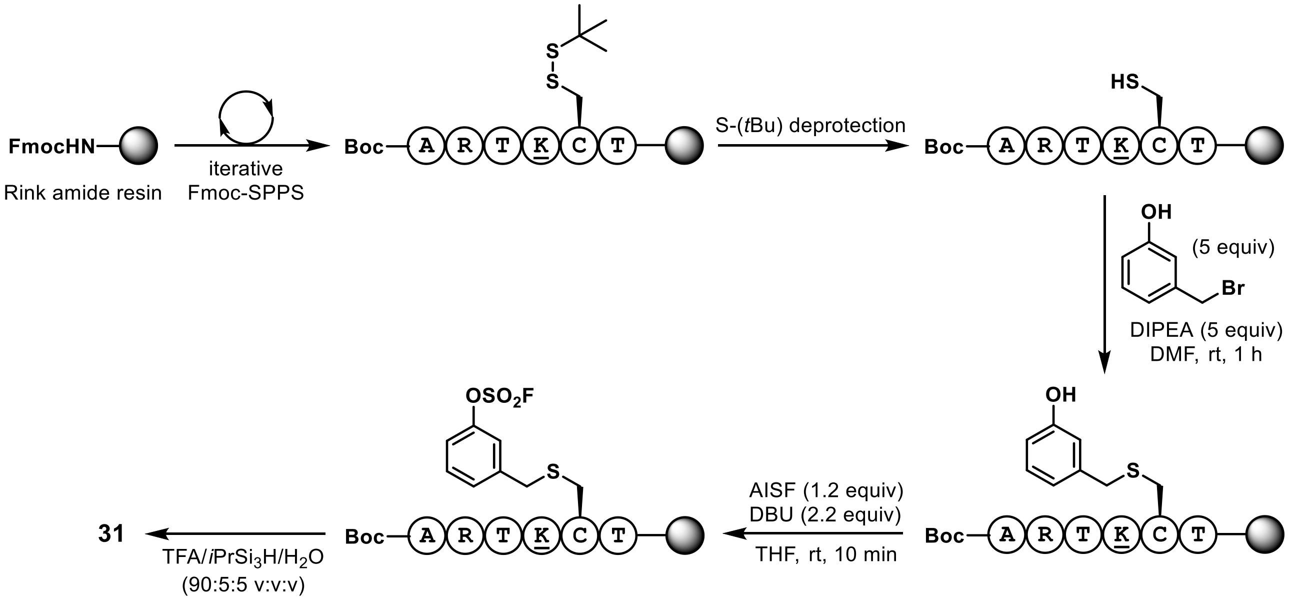

Hexapeptide Boc-Ala-Arg(Pbf)-Thr(*t*Bu)-K(me3)-Cys(S*t*Bu)-Thr(*t*Bu)-CONH-Rink amide resin (1 equiv) was prepared on Rink amide resin according to the procedures outlined in the **Iterative Fmoc-SPPS workflow**. The S-(*t*Bu) group was deprotected according to the protocols outlined in the **Deprotection procedures**. The resin was washed with DMF (5 × 3 mL) and treated with a solution of DIPEA (5 equiv) and 3-(bromomethyl)phenol (5 equiv) in DMF and agitated at room temperature for 30 minutes or until consumption of starting material was confirmed by LCMS analysis. The liquid was discarded, and the resin was washed with DMF (5 × 3 mL), DCM (5 × 3 mL), DMF (5 × 3 mL) and DCM (5 × 3 mL). Installation of the fluorosulfate group was achieved using the synthetic procedures outlined for arylfluorosulfate peptides **30** and **32**. The peptide was cleaved from resin and purified according to the procedures outlined in the **Iterative Fmoc-SPPS workflow** to afford arylfluorosulfate peptide **31**.

Compound **31**

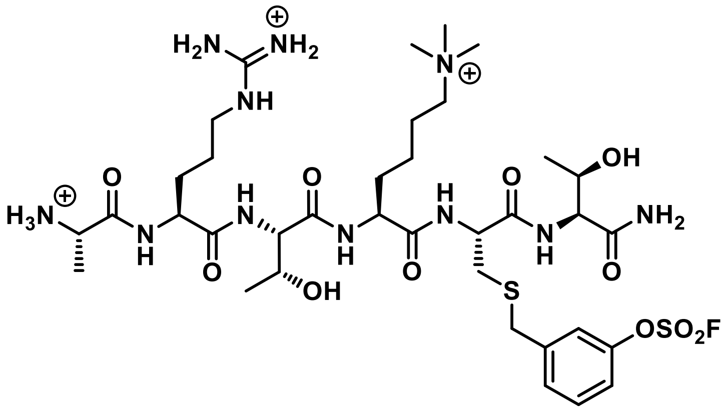

Yield: 16%

Exact mass: 910.43 $[C_{36}H_{65}FN_{11}O_{11}S_2]^{3+}$

HPLC trace (Method 2):

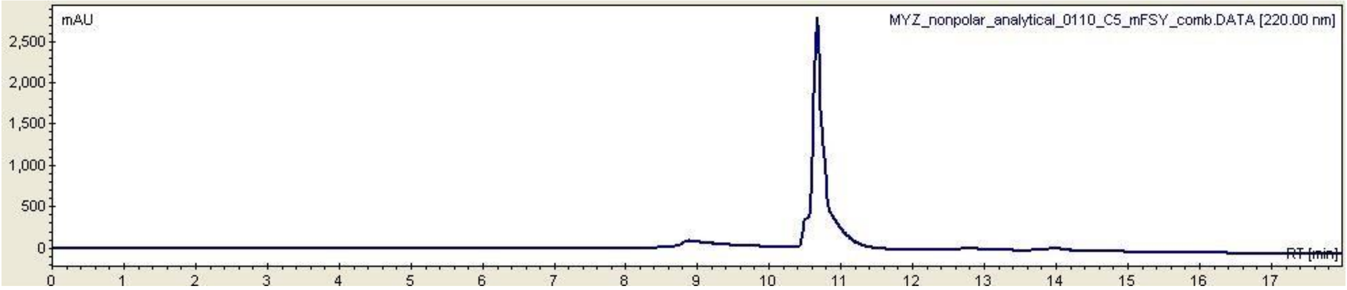

LCMS

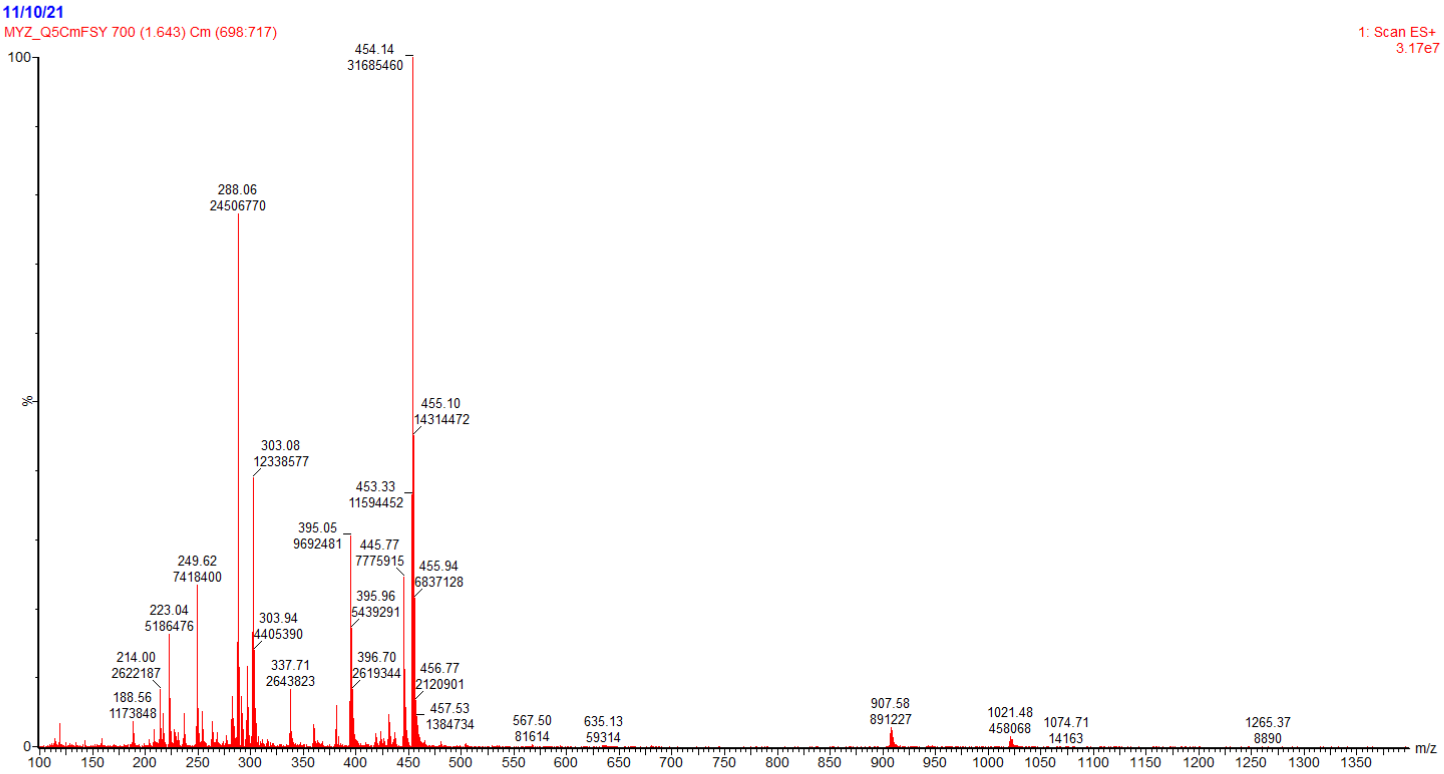

MALDI

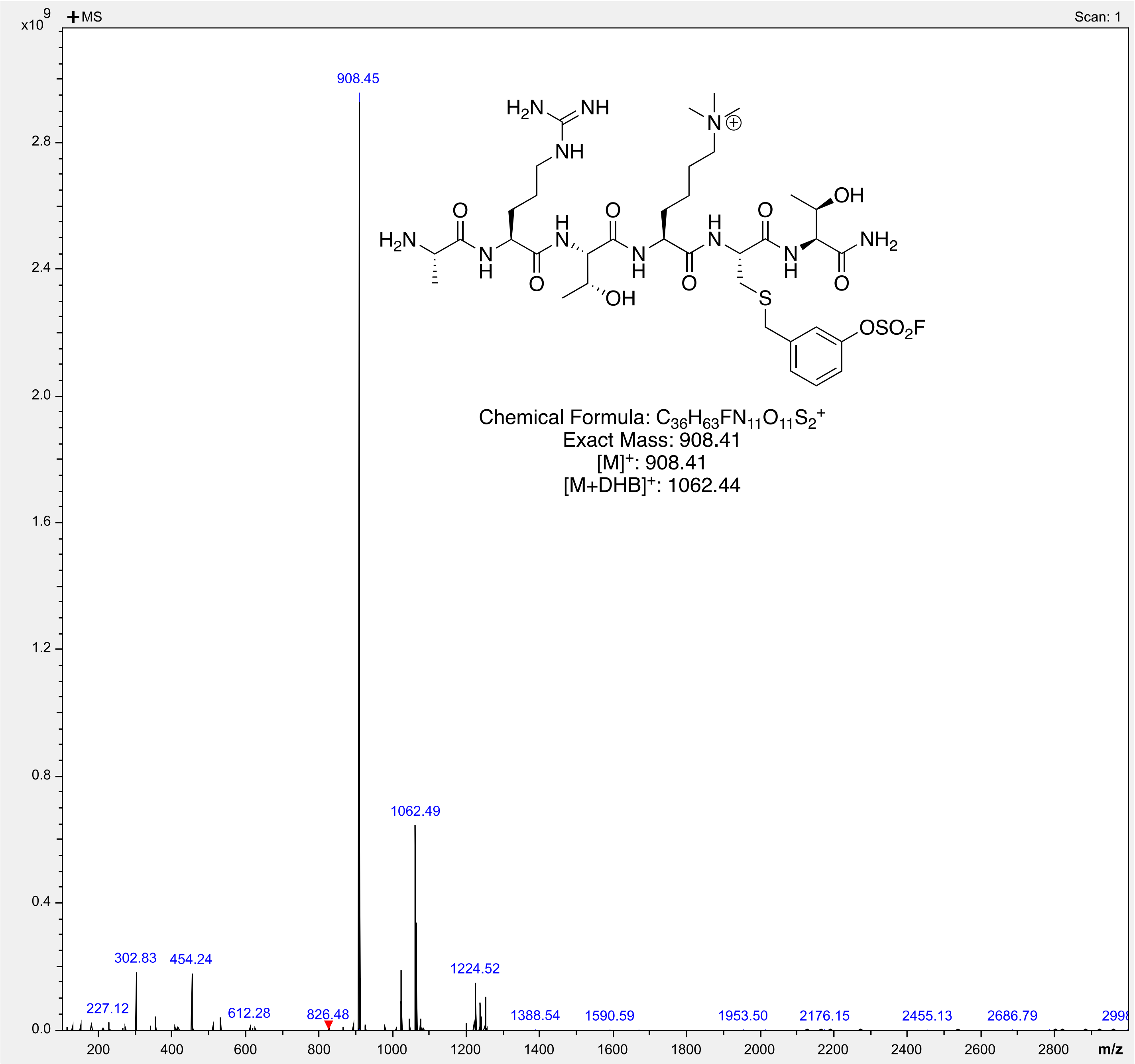

x10^9
+MS
Scan: 1
908.45
1062.49
302.83
454.24
1224.52
227.12
612.28
826.48
1388.54
1590.59
1953.50
2176.15
2455.13
2686.79
m/z
Chemical Formula: C36H63FN11O11S2+
Exact Mass: 908.41
[M]+: 908.41
[M+DHB]+: 1062.44

Installation of the 4-benzylsulfonyl fluoride covalent warhead

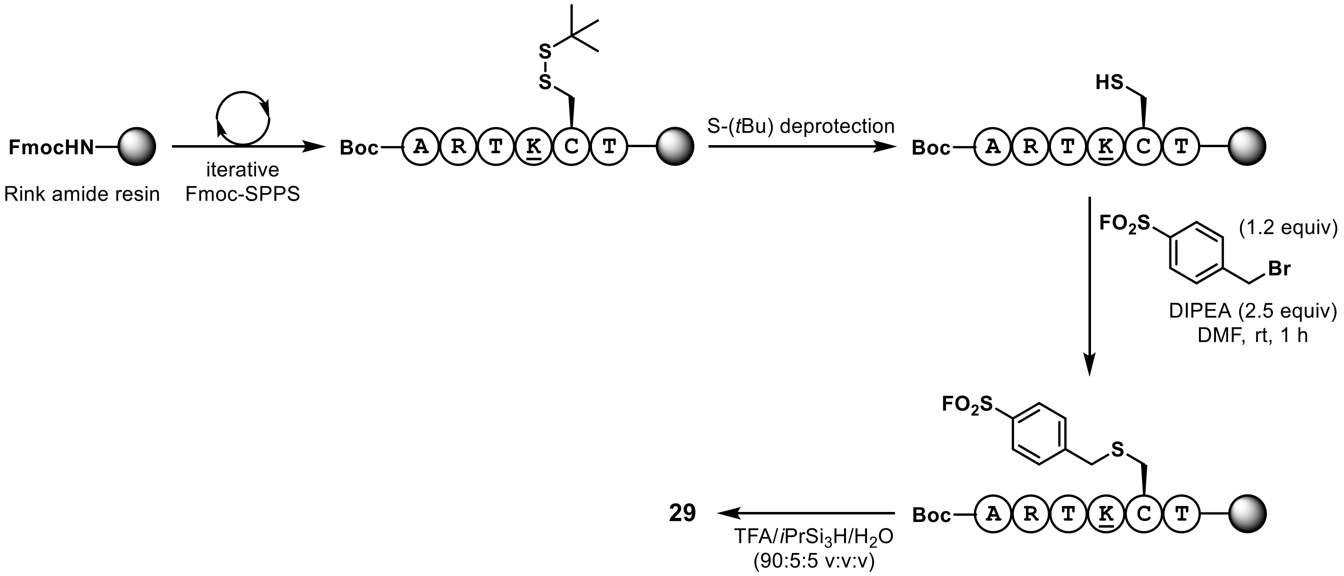

Hexapeptide Boc-Ala-Arg(Pbf)-Thr(*t*Bu)-K(me3)-Cys(S*t*Bu)-Thr(*t*Bu)-CONH-Rink amide resin (1 equiv) was prepared on Rink amide resin according to the procedures outlined in the **Iterative Fmoc-SPPS workflow**. The S-(*t*Bu) group was deprotected according to the protocols outlined in **Deprotection procedures**. The resin was washed with DMF (5 × 3 mL) and treated with a solution of DIPEA (2.5 equiv) and 4-(bromomethyl)benzenesulfonyl fluoride (1.2 equiv) in DMF and agitated at room temperature for 30 minutes or until consumption of starting material was confirmed (monitored by LCMS analysis). The liquid was discarded, and the resin was washed with DMF (5 × 3 mL), DCM (5 × 3 mL), DMF (5 × 3 mL) and DCM (5 × 3 mL). The peptide was cleaved and purified according to the procedures outlined in the **Iterative Fmoc-SPPS workflow** to afford sulfonyl fluoride peptide **29**.

Compound **29**

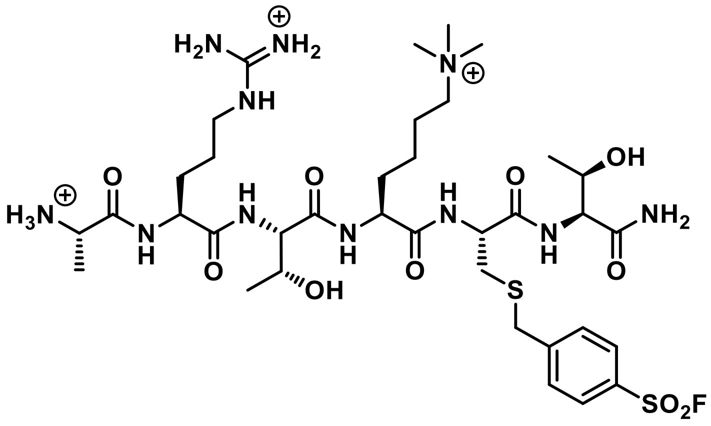

Yield:14%

Exact mass: 894.43 $[C_{36}H_{65}FN_{11}O_{10}S_2]^{3+}$

HPLC trace (Method 2):

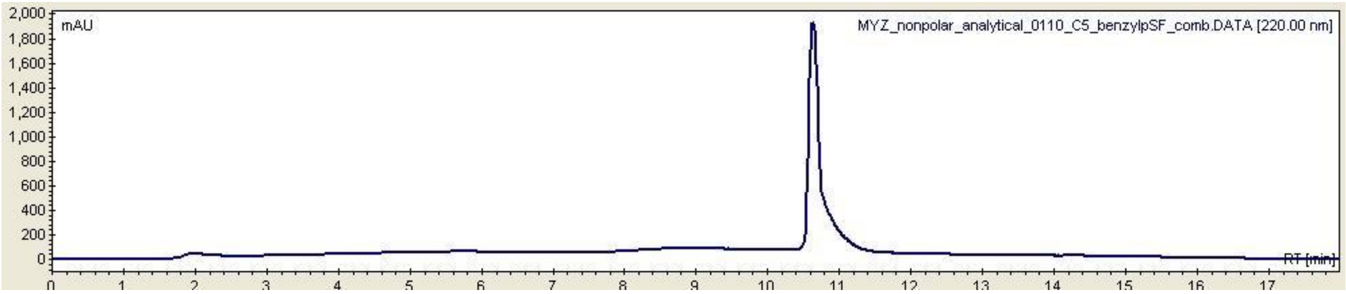

LCMS

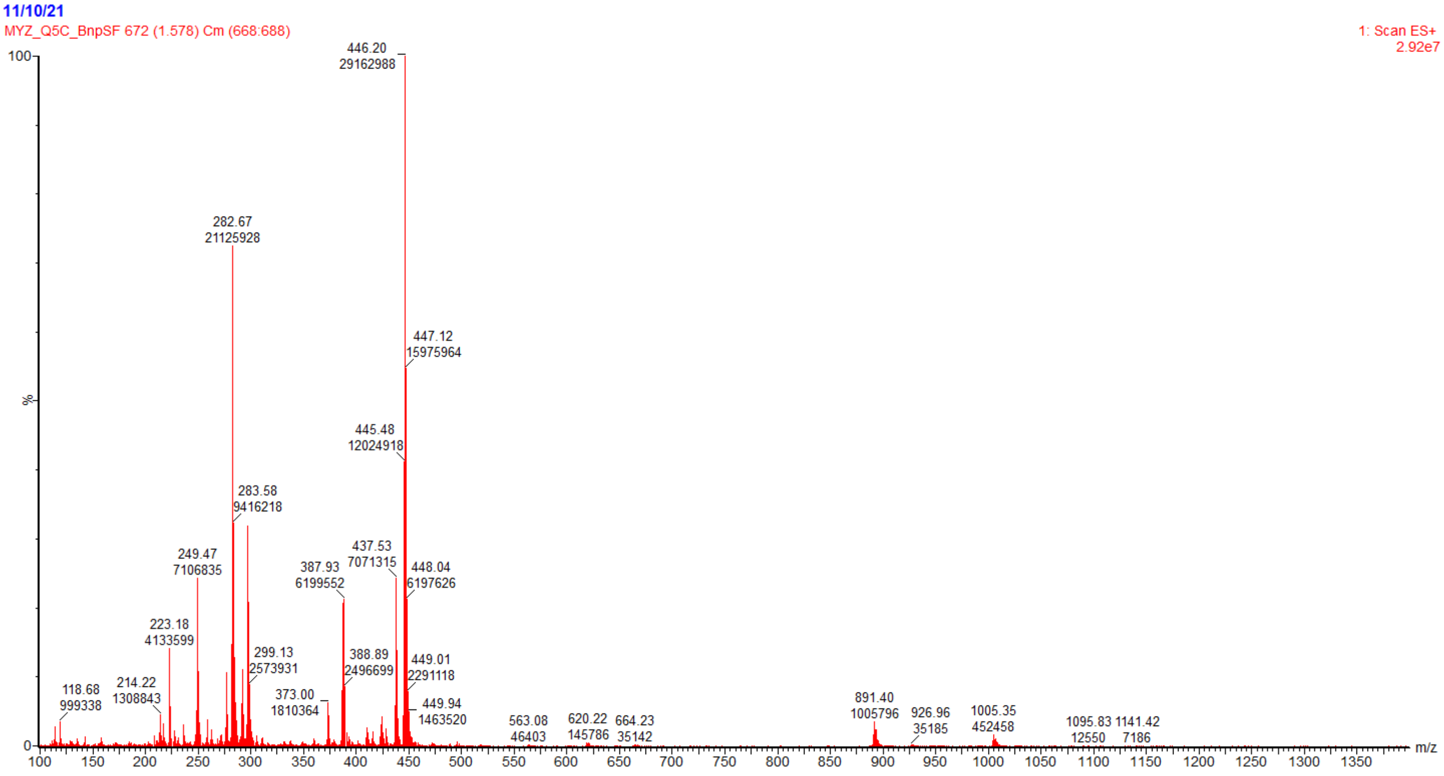

MALDI

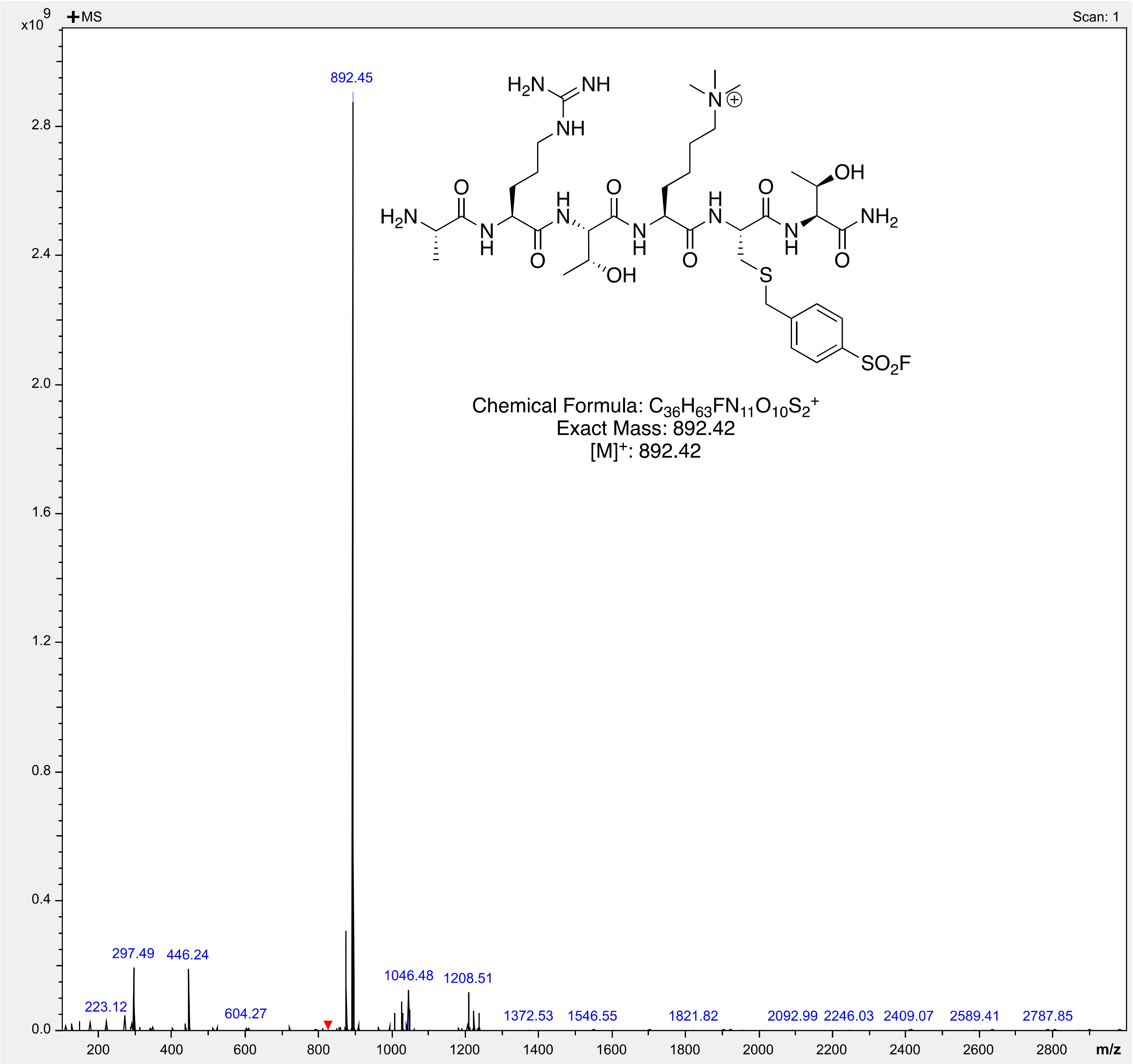
x10^9
+MS
Scan: 1
892.45
297.49
446.24
223.12
604.27
1046.48
1208.51
1372.53
1546.55
1821.82
2092.99
2246.03
2409.07
2589.41
2787.85
m/z
Chemical Formula: C36H63FN11O10S2+
Exact Mass: 892.42
[M]+: 892.42

Installation of the fluorosulfonylbenzamide covalent warhead

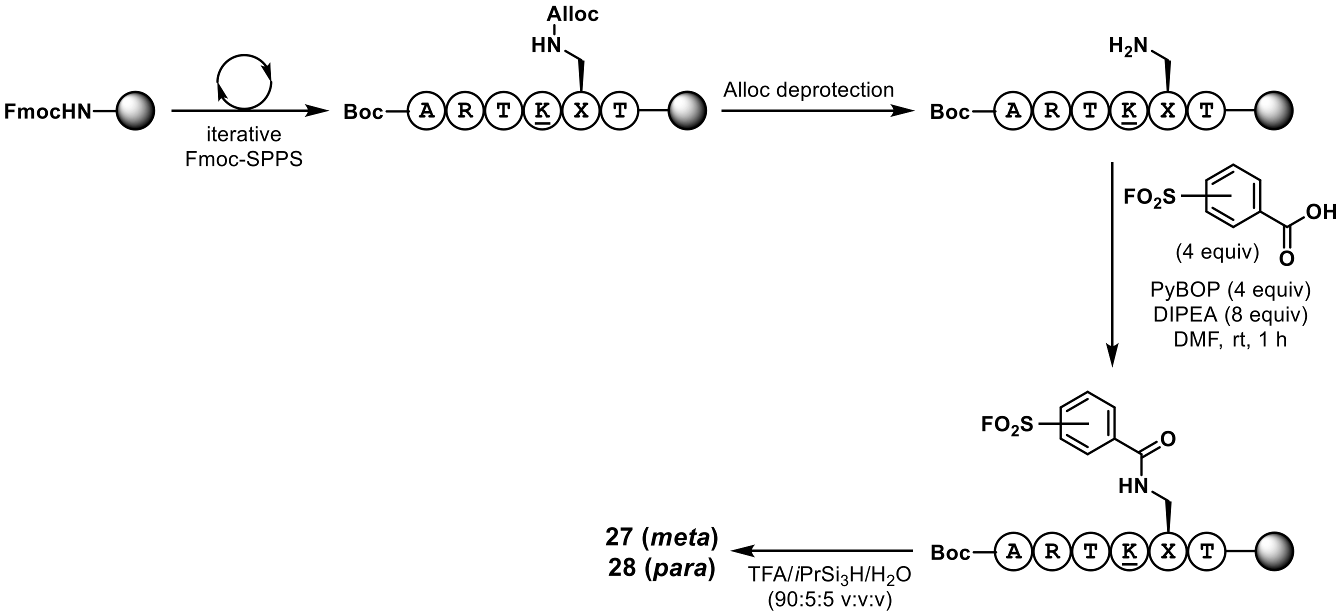

27 (***meta***)
28 (***para***)

Hexapeptide Boc-Ala-Arg(Pbf)-Thr(*t*Bu)-K(me3)-Dap(Alloc)-Thr(*t*Bu)-CONH-Rink amide resin (1 equiv) was prepared on Rink amide resin according to the procedures outlined in the **Iterative Fmoc-SPPS workflow**. The Alloc group was deprotected according to the protocols outlined in **Deprotection procedures**. The resin was washed with DMF (5 × 3 mL), DCM (5 × 3 mL), DMF (5 × 3 mL). The Alloc-deprotected resin was treated with a solution of *para*, or *meta*-fluorosulfonylbenzoic acid (4 equiv), PyBOP (4 equiv) and DIPEA (8 equiv) in DMF and agitated at room temperature for one hour or until consumption of starting material was confirmed (monitored by LCMS analysis), at which time the liquid was discarded and the resin was washed with DMF (5 × 3 mL), DCM (5 × 3 mL), DMF (5 × 3 mL) and DCM (5 × 3 mL). The peptide was cleaved from resin and purified according to the procedures outlined in the **Iterative Fmoc-SPPS workflow** to afford **27** and **28**.

Compound **28**

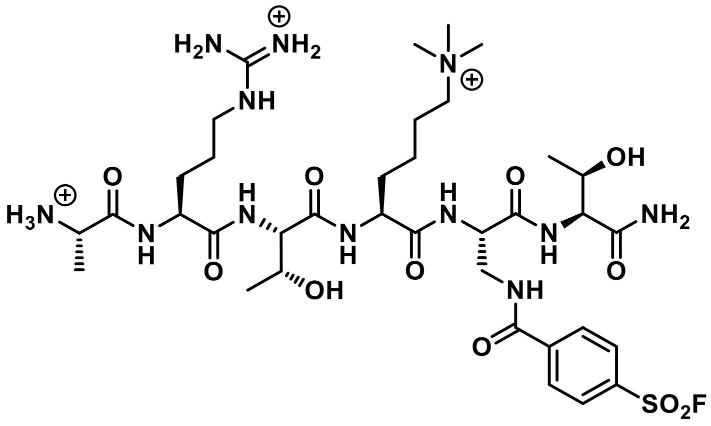

Yield: 20%

Exact mass: 891.45 $[C_{36}H_{64}FN_{12}O_{11}S]^{3+}$

HPLC trace (Method 2):

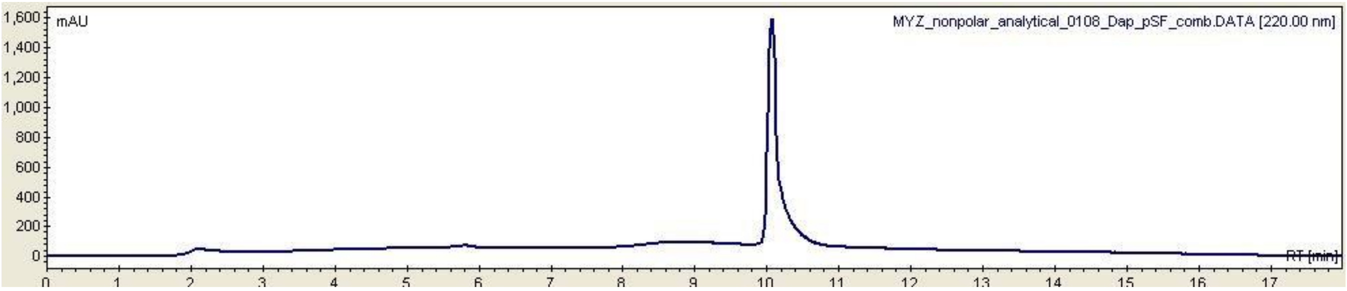

LCMS

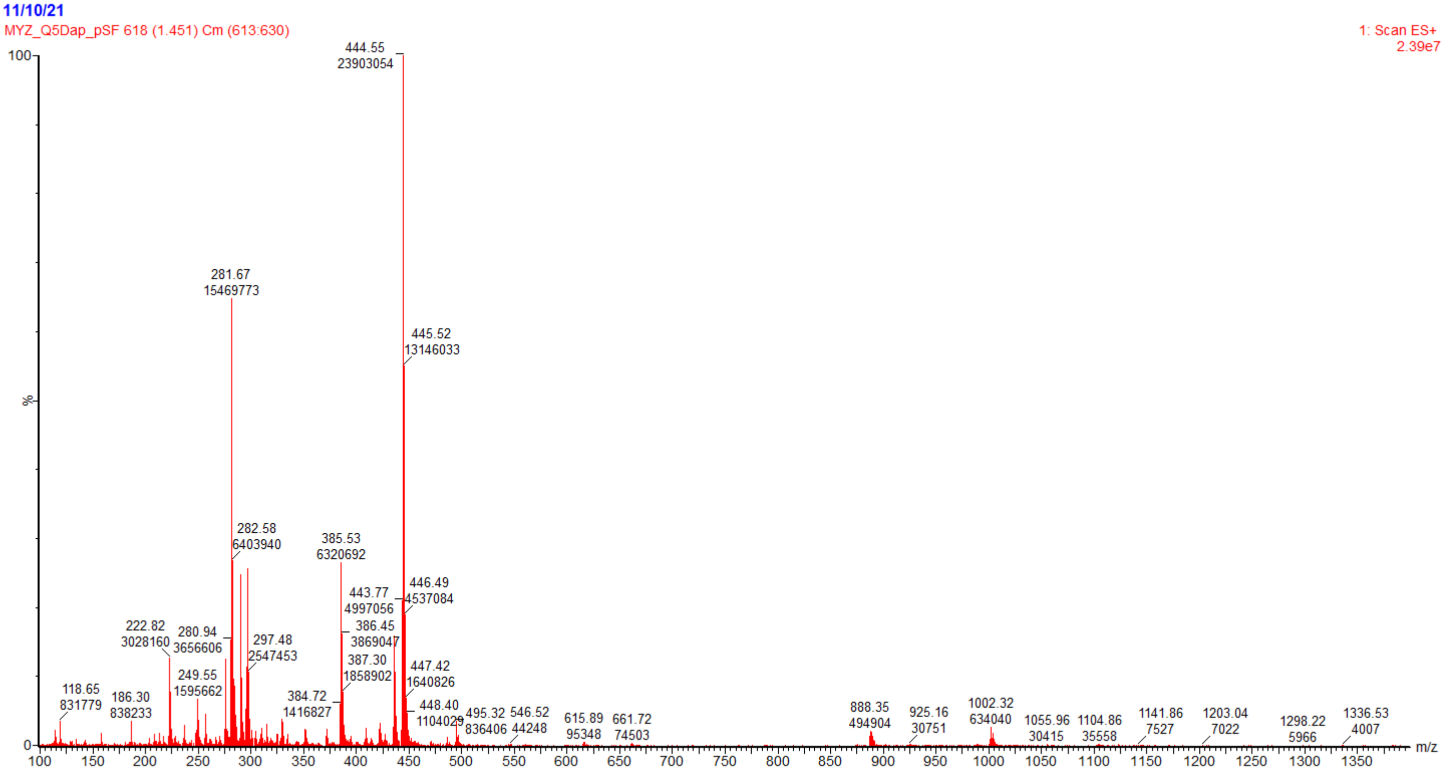

MALDI

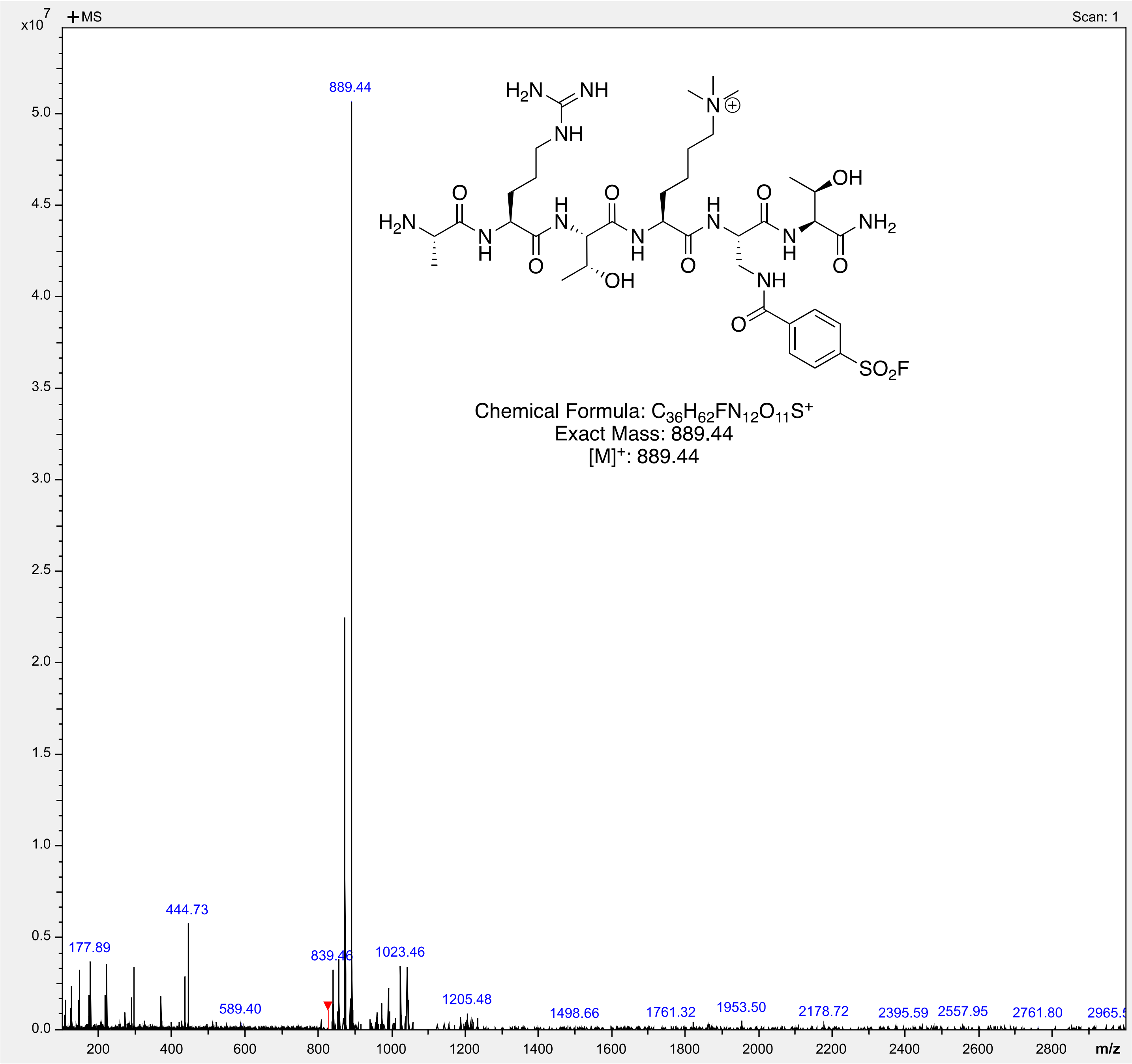
x10^7 +MS
Scan: 1
889.44
444.73
177.89
589.40
839.46
1023.46
1205.48
1498.66
1761.32
1953.50
2178.72
2395.59
2557.95
2761.80
m/z
Chemical Formula: C36H62FN12O11S+
Exact Mass: 889.44
[M]+: 889.44

Compound **27**

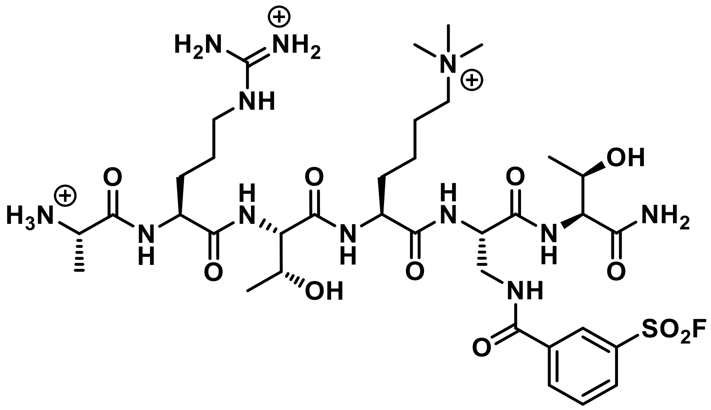

Yield: 10%

Exact mass: 891.45 $[C_{36}H_{64}FN_{12}O_{11}S]^{3+}$

HPLC trace (Method 2):

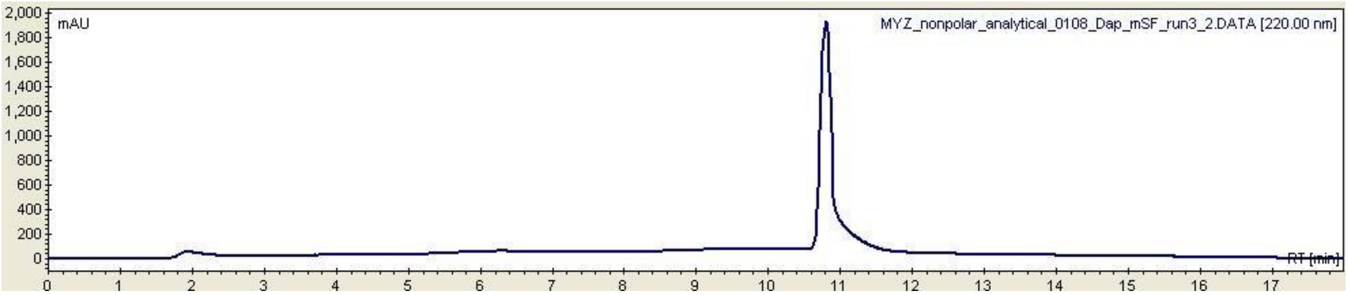

LCMS

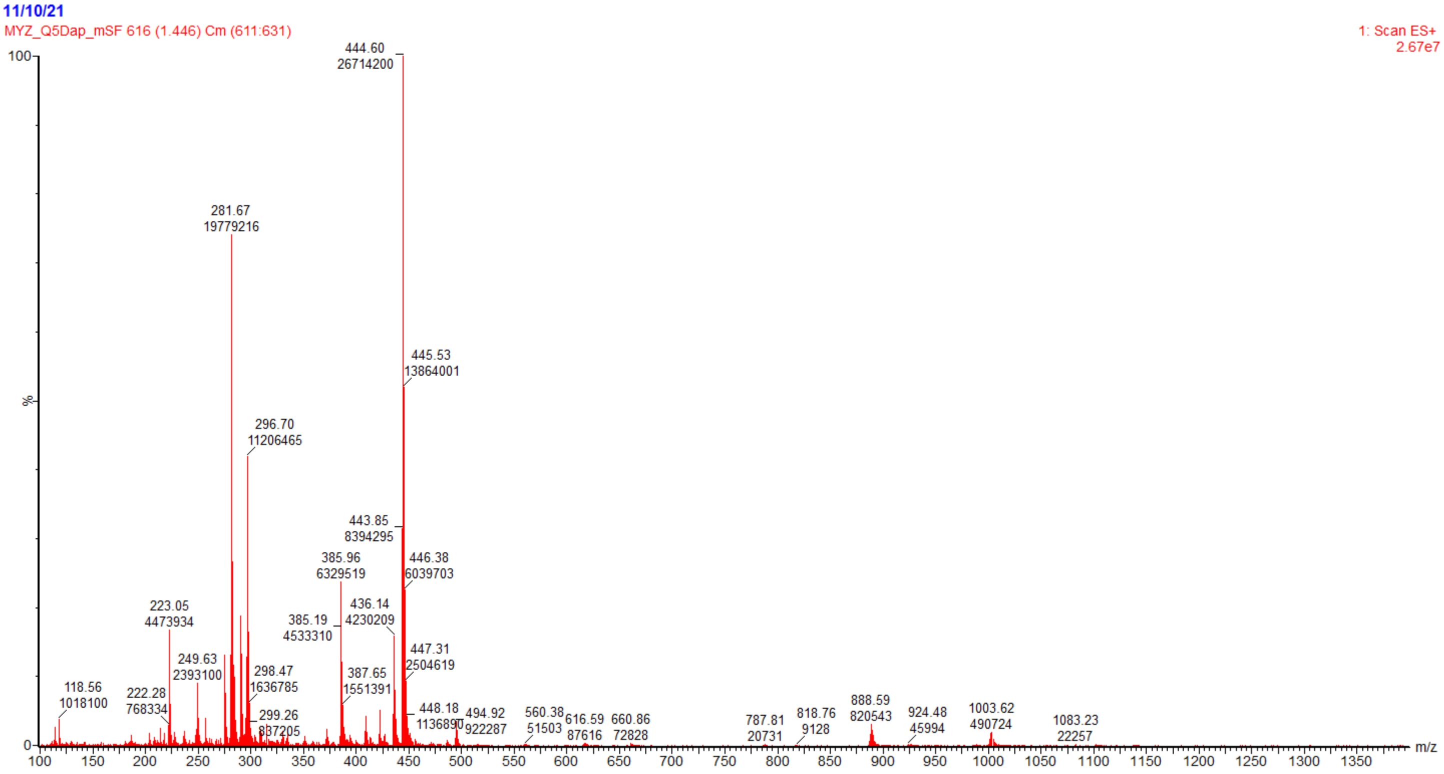

MALDI

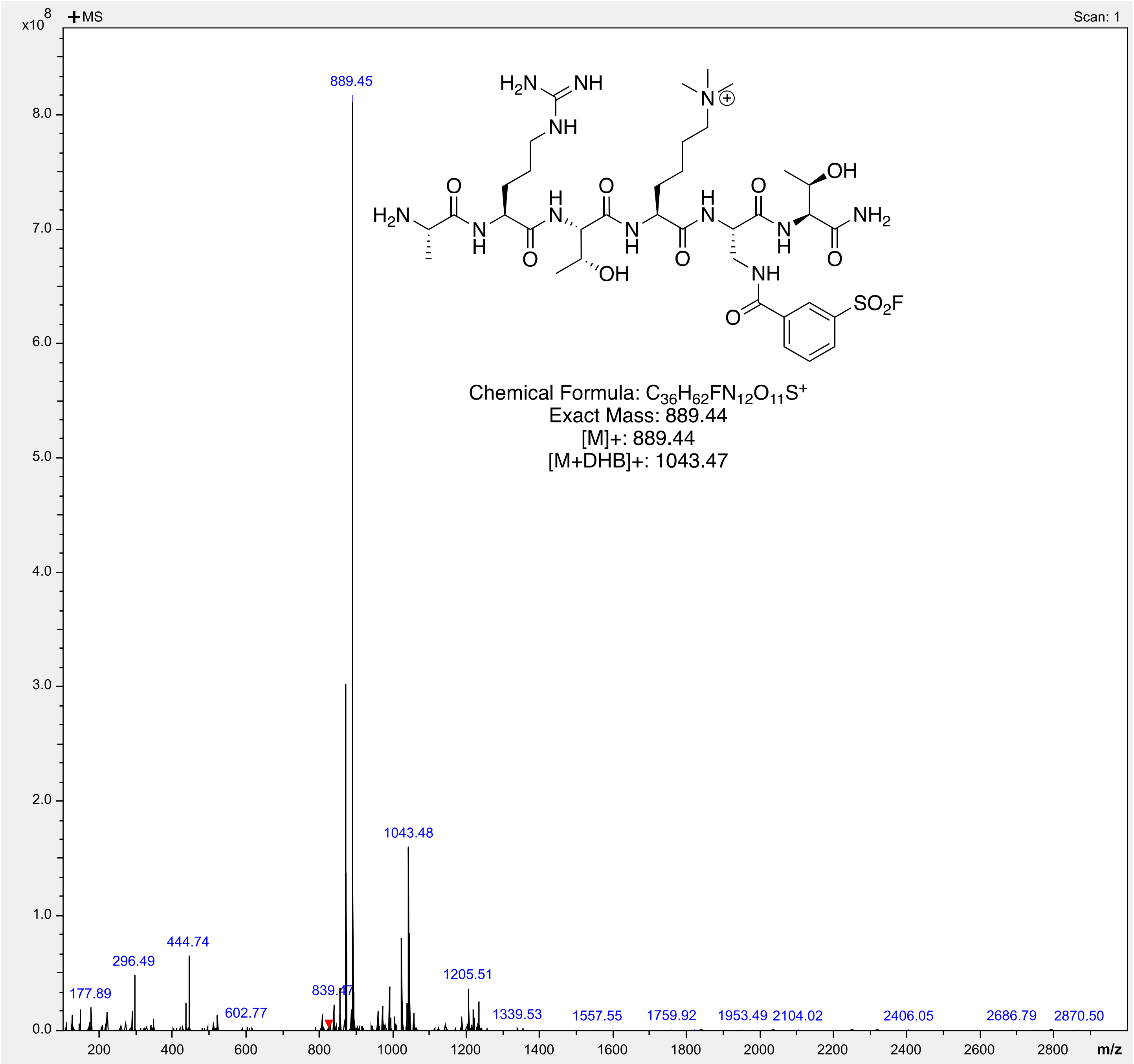

x10^8 +MS
Scan: 1
889.45
1043.48
1205.51
444.74
296.49
177.89
602.77
839.47
1339.53
1557.55
1759.92
1953.49
2104.02
2406.05
2686.79
2870.50
m/z
Chemical Formula: C36H62FN12O11S+
Exact Mass: 889.44
[M]+: 889.44
[M+DHB]+: 1043.47

Synthesis of covalent cyclopeptides

Synthesis of covalent thioether cyclopeptide C-33

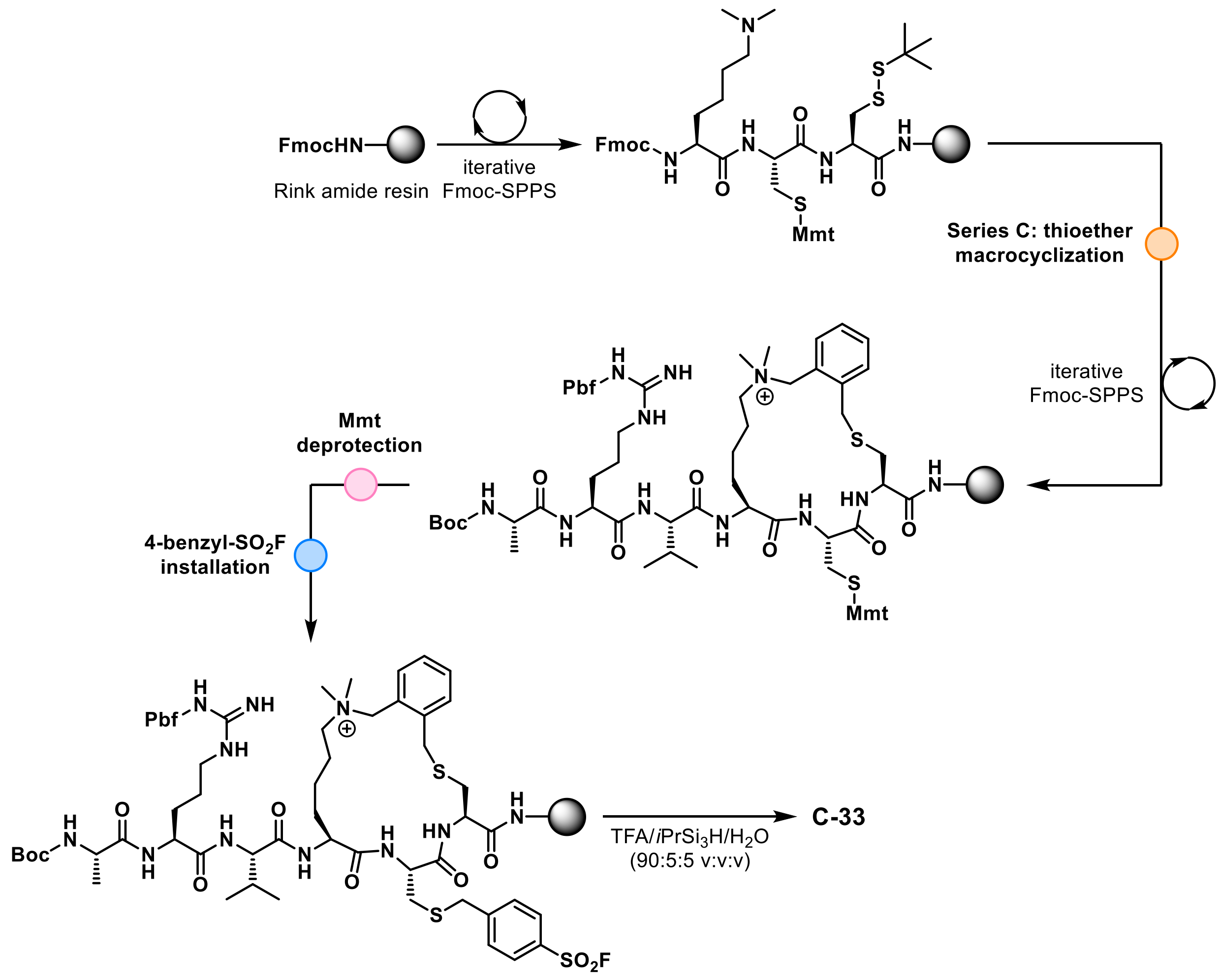

Tripeptide Fmoc-Lys(me2)-Cys(Mmt)-Cys(S*t*Bu)-CONH-Rink amide resin (1 equiv) was prepared on Rink amide resin according to the procedures outlined in the **Iterative Fmoc-SPPS workflow**. The tripeptide intermediate was subjected to **Series C: thioether macrocyclization** conditions and elongated using the procedures outlined in the **Iterative Fmoc-SPPS workflow**. The Mmt group was cleaved using the procedures described in **Deprotection procedures**, and the 4-benzylsulfonyl fluoride functionality was introduced according to the protocols outlined in **Installation of the 4-benzylsulfonyl fluoride covalent warhead**. The resin was washed with DMF (5 × 3 mL), DCM (5 × 3 mL), DMF (5 × 3 mL) and DCM (5 × 3 mL). The peptide was cleaved from resin and purified according to the procedures outlined in the **Iterative Fmoc-SPPS workflow** to afford **C-33**.

Compound **C-33**

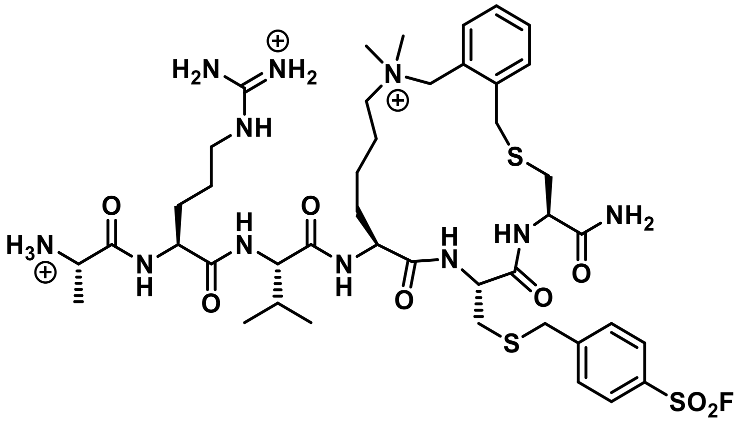

Yield: 6%

Exact mass: 982.45 $[C_{43}H_{69}FN_{11}O_8S_3]^{3+}$

HPLC trace (Method 3):

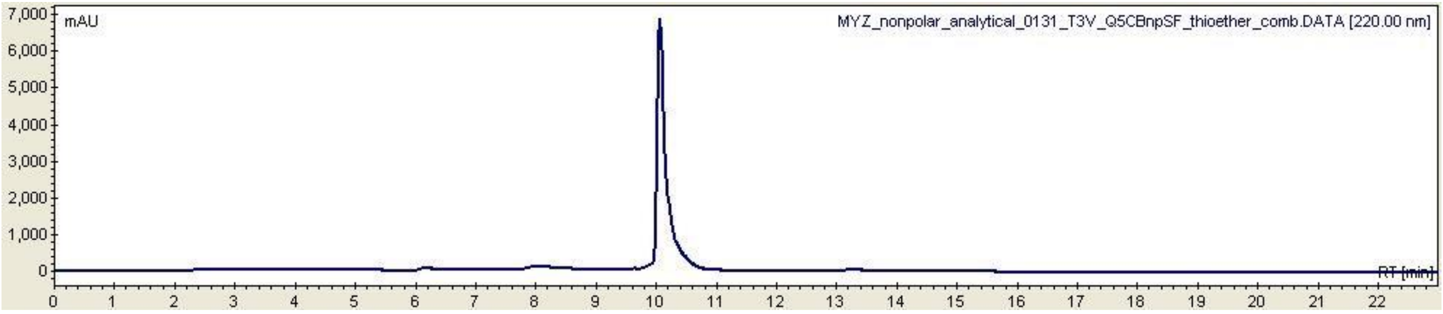

LCMS

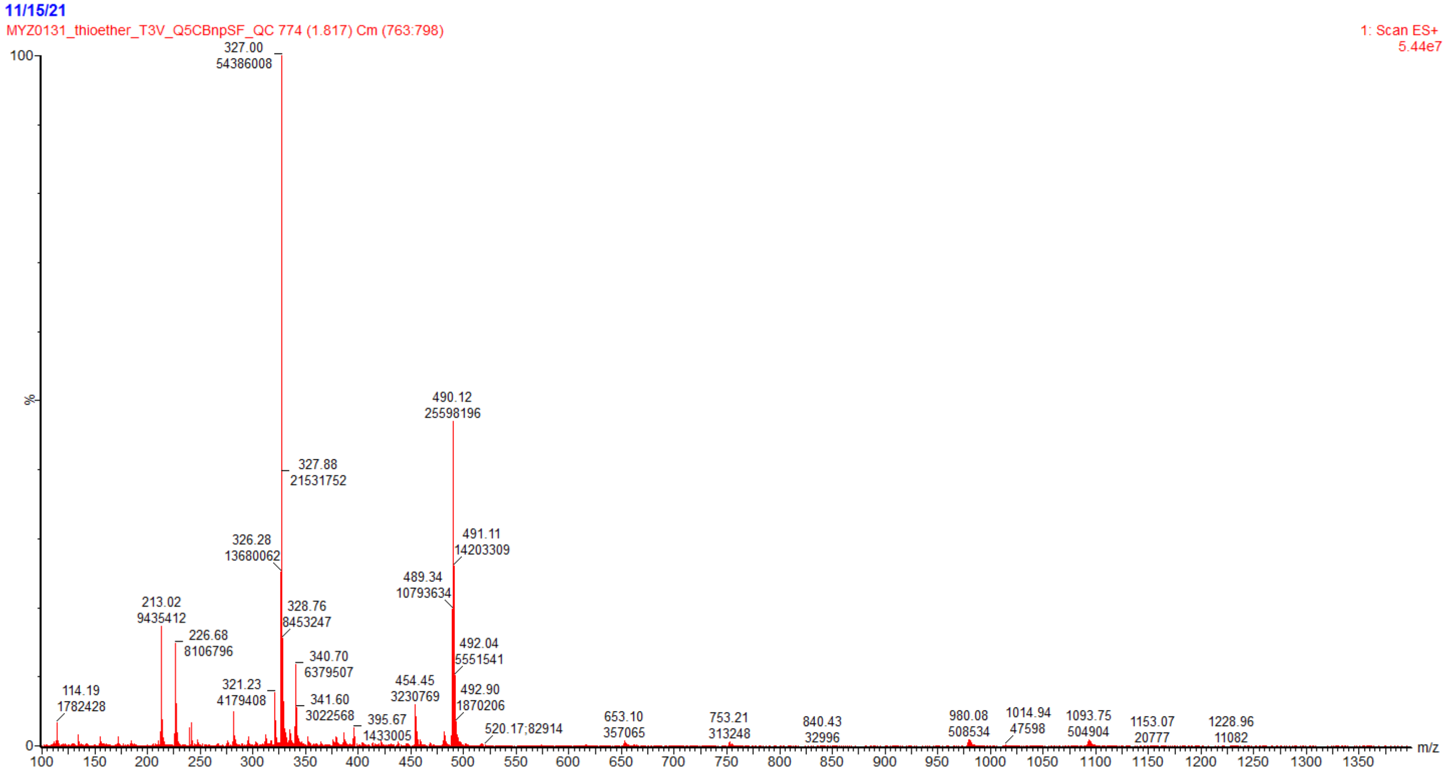

MALDI

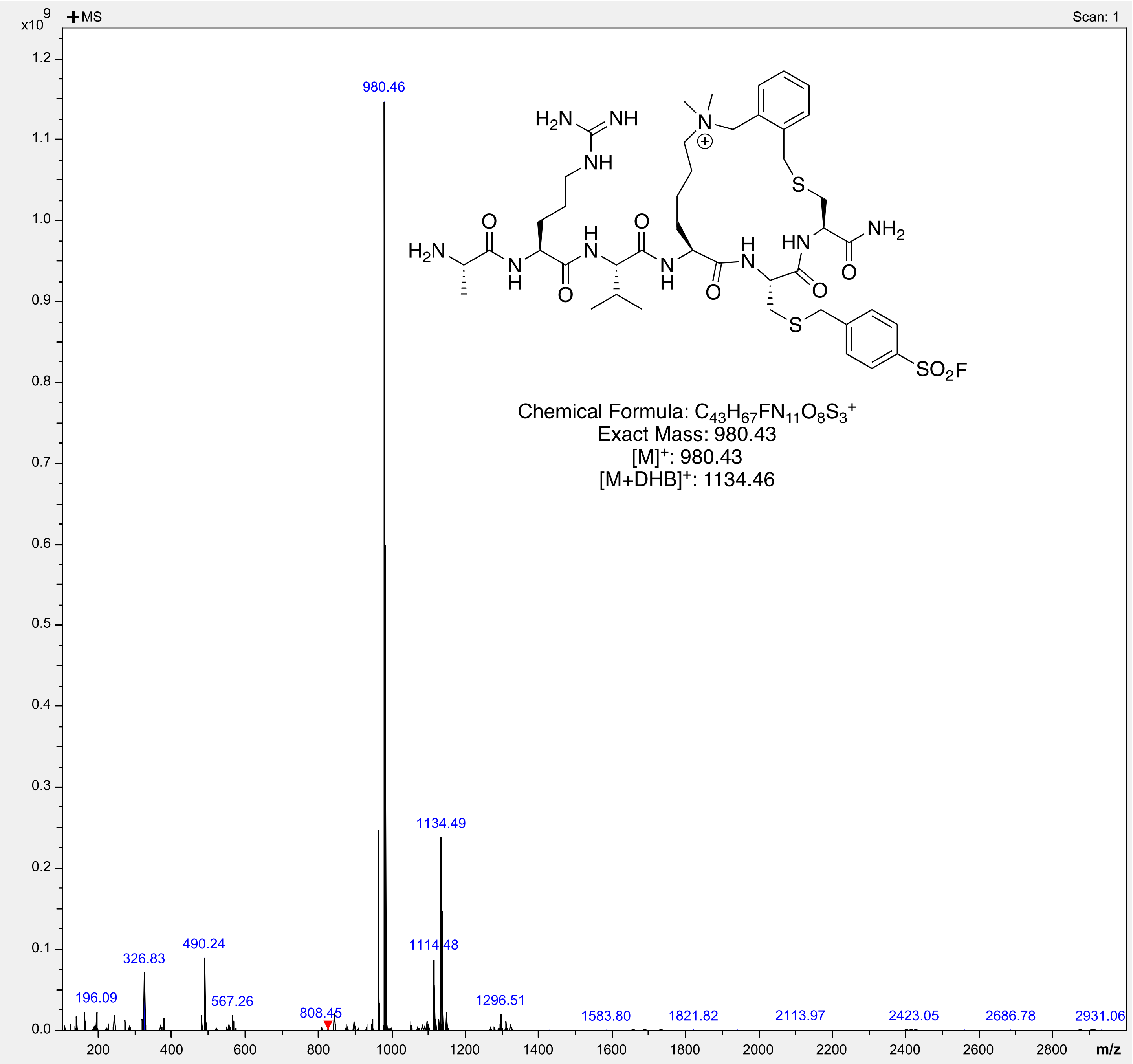

x10^9
+MS
Scan: 1
980.46
1134.49
1114.48
326.83
490.24
196.09
567.26
808.45
1296.51
1583.80
1821.82
2113.97
2423.05
2686.78
2931.06
m/z
Chemical Formula: C43H67FN11O8S3+
Exact Mass: 980.43
[M]+: 980.43
[M+DHB]+: 1134.46

Synthesis of covalent thioether peptide D-34 and D-35

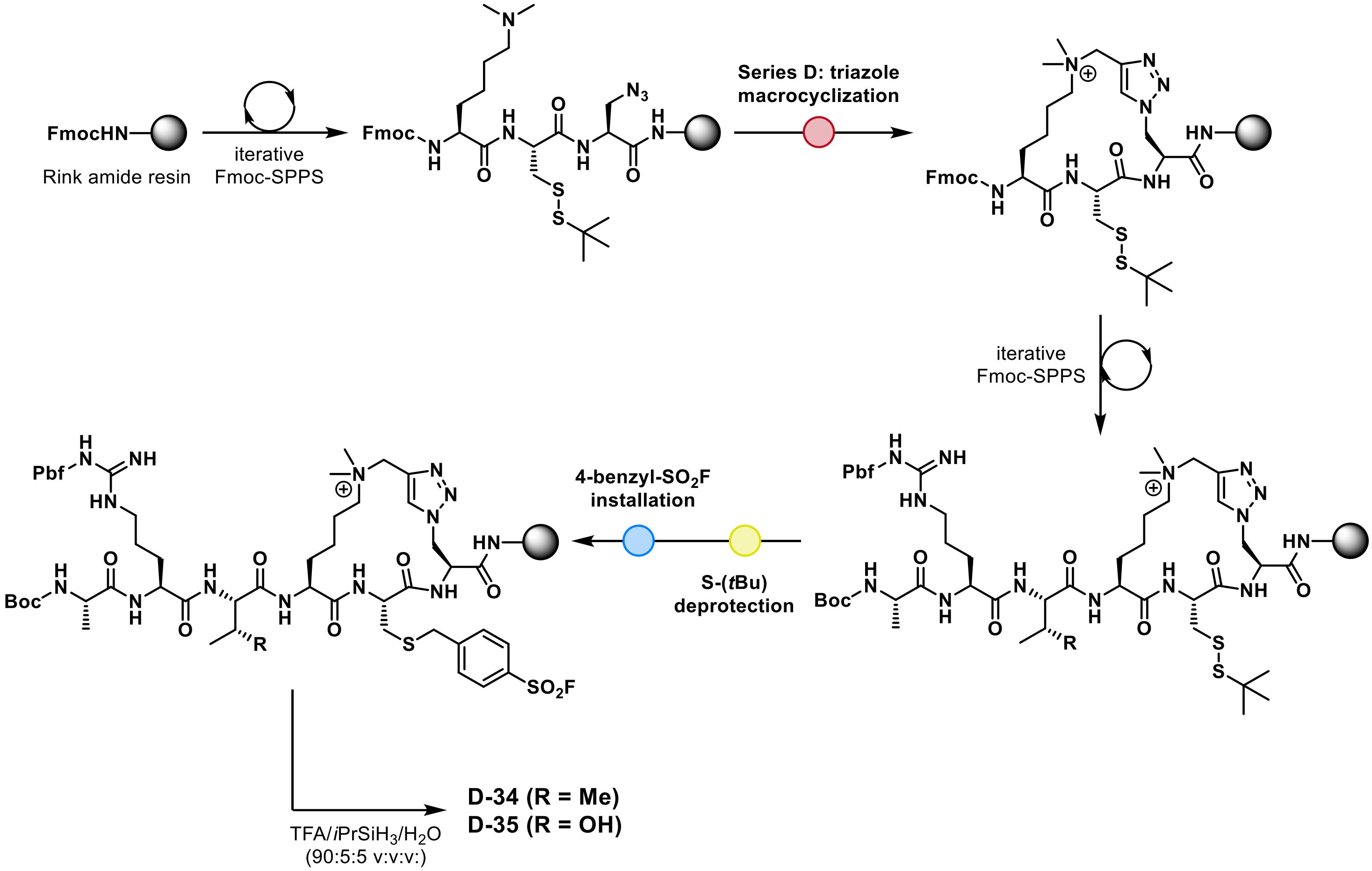

D-34 (R = Me)
D-35 (R = OH)

Tripeptide Fmoc-Lys(me2)-Cys(*St*Bu)-Dap(N_3)-CONH-Rink amide resin (1 equiv) was prepared on Rink amide resin according to the procedures outlined in the **Iterative Fmoc-SPPS workflow**. The tripeptide intermediate was subjected to **Series D: triazole macrocyclization** conditions and elongated using the procedures outlined in the **Iterative Fmoc-SPPS workflow**. The S-(*t*Bu) group was cleaved using the procedures described in **Deprotection procedures**, and the 4-benzylsulfonyl fluoride functionality was introduced according to the protocols outlined in **Installation of the 4-benzylsulfonyl fluoride covalent warhead**. The resin was washed with DMF (5 × 3 mL), DCM (5 × 3 mL), DMF (5 × 3 mL) and DCM (5 × 3 mL). The peptide was cleaved from resin and purified according to the procedures outlined in the **Iterative Fmoc-SPPS workflow** to afford **D-34 and D-35**.

Compound **D-34**

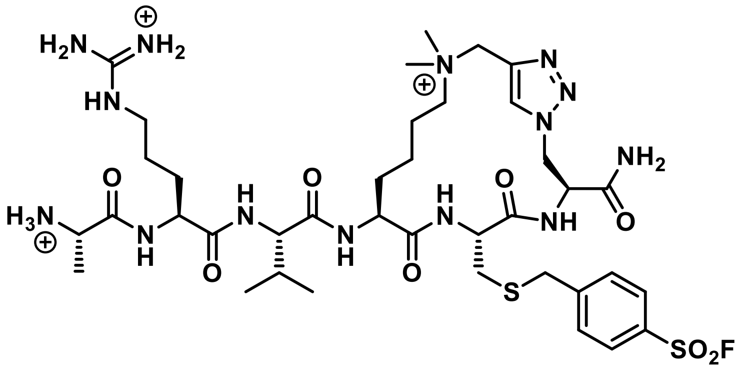

Yield: 2%

Exact mass: 927.44 $[C_{38}H_{64}FN_{14}O_8S_2]^{3+}$

HPLC trace (Method 2):

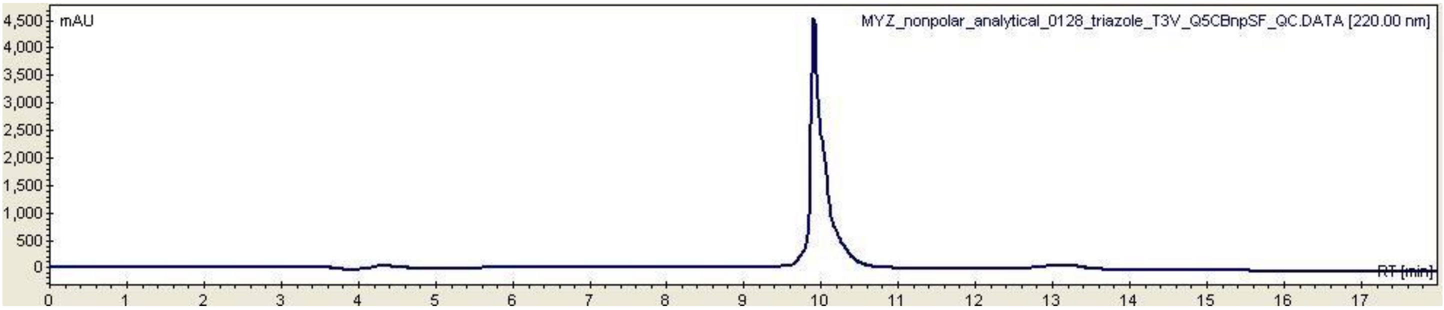

LCMS

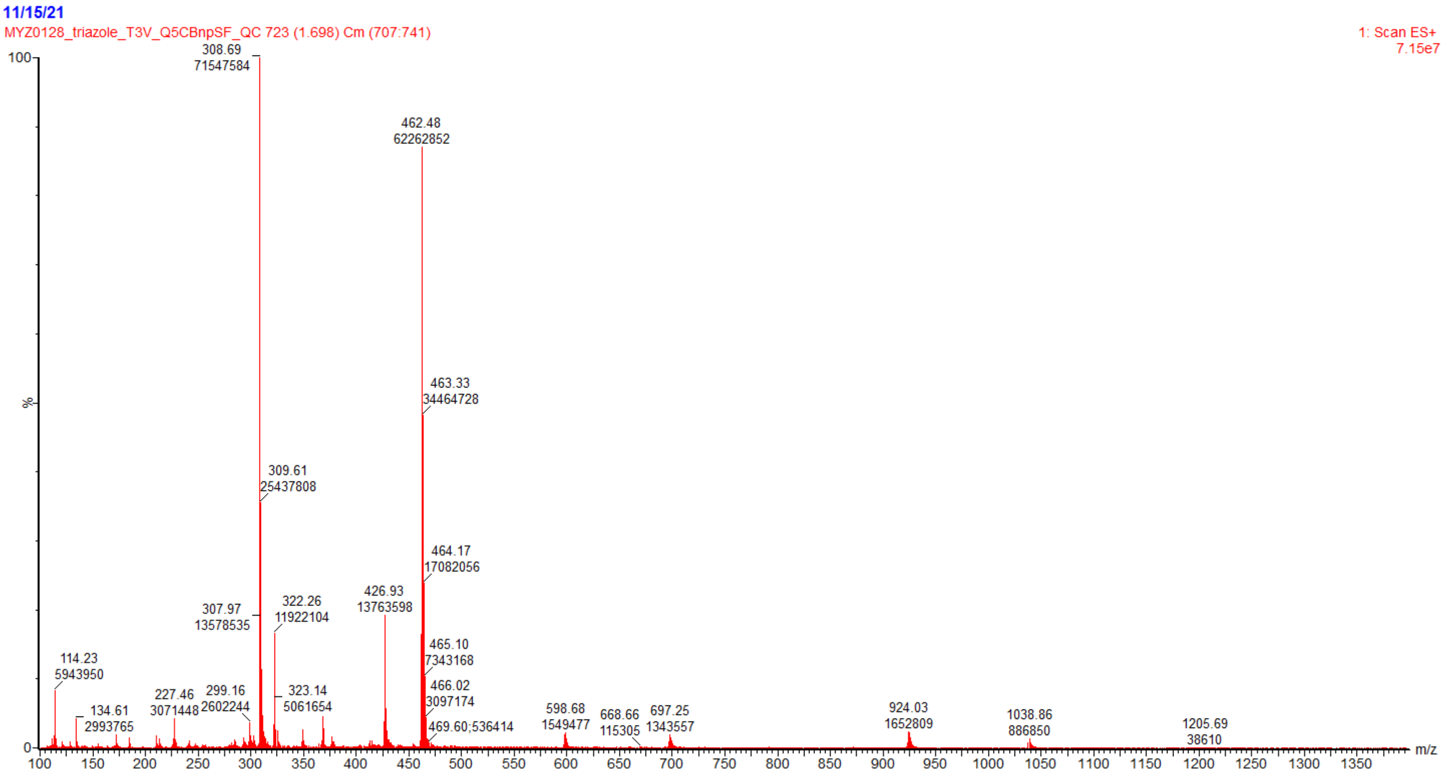

MALDI

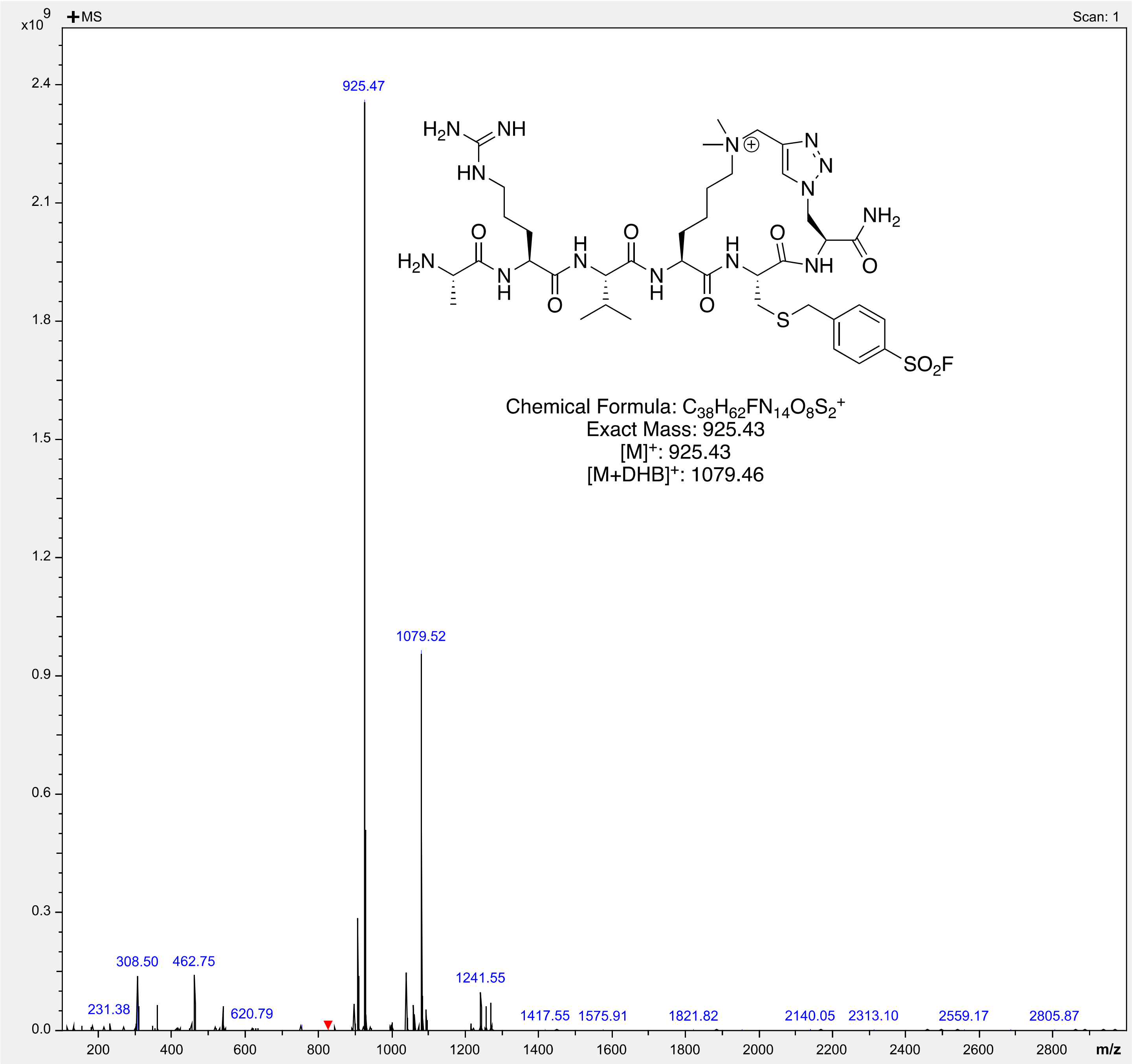
x10^9
+MS
Scan: 1
925.47
1079.52
308.50
462.75
231.38
620.79
1241.55
1417.55
1575.91
1821.82
2140.05
2313.10
2559.17
2805.87
m/z
Chemical Formula: C38H62FN14O8S2+
Exact Mass: 925.43
[M]+: 925.43
[M+DHB]+: 1079.46

Compound **D-35**

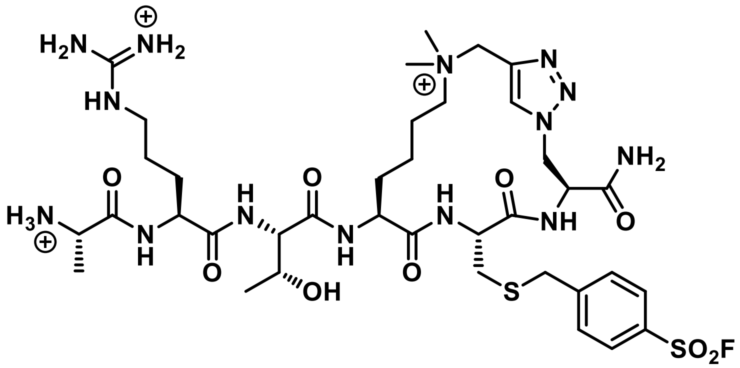

Yield: 4%

Exact mass: 929.42 $[C_{37}H_{62}FN_{14}O_9S_2]^{3+}$

HPLC trace (Method 2):

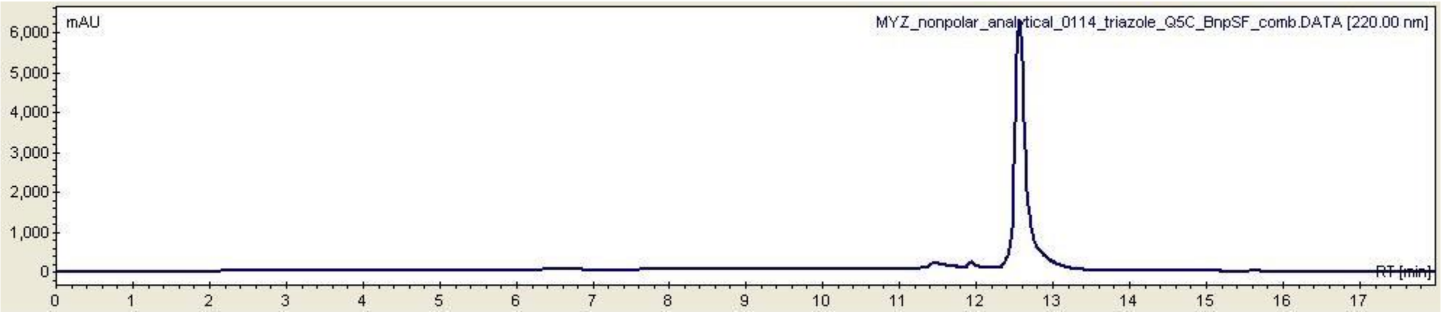

LCMS

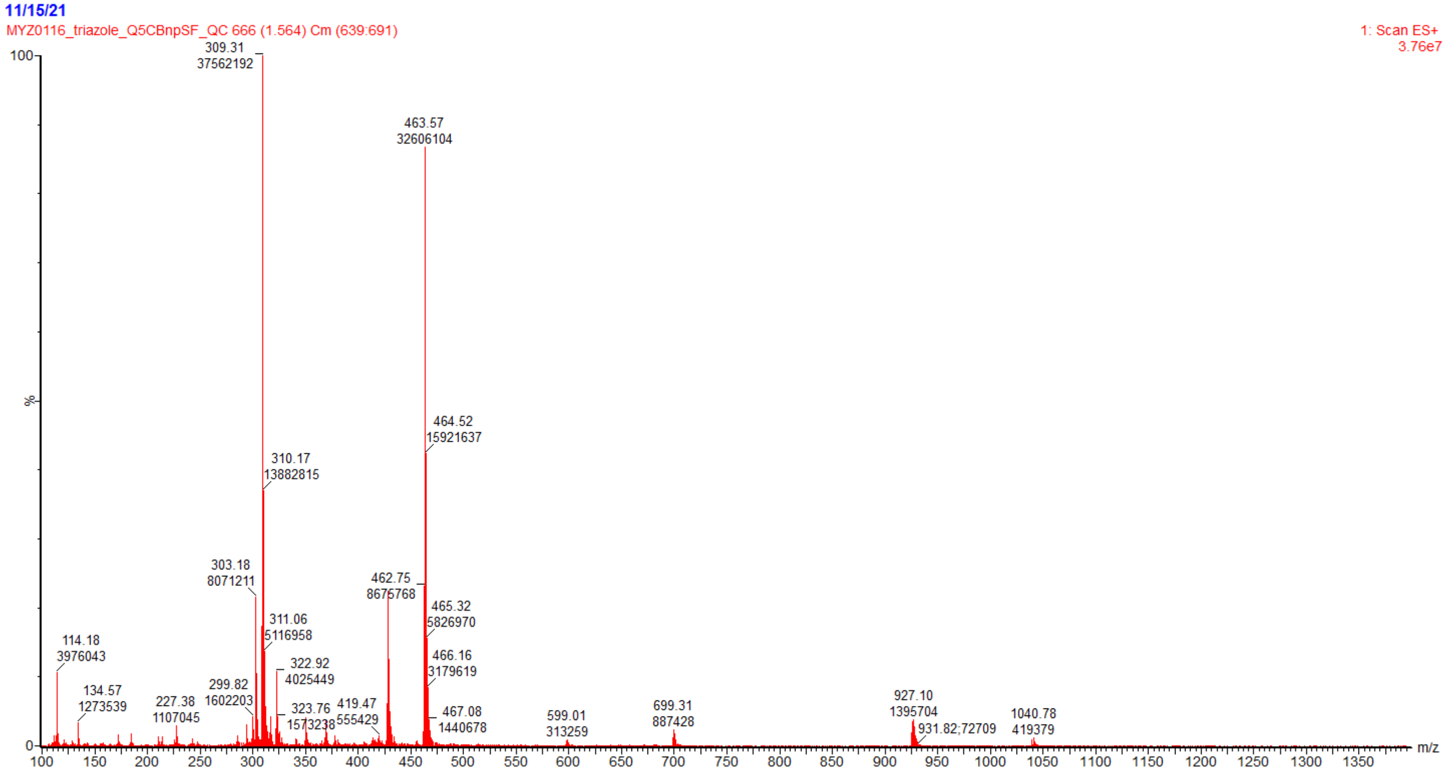

MALDI

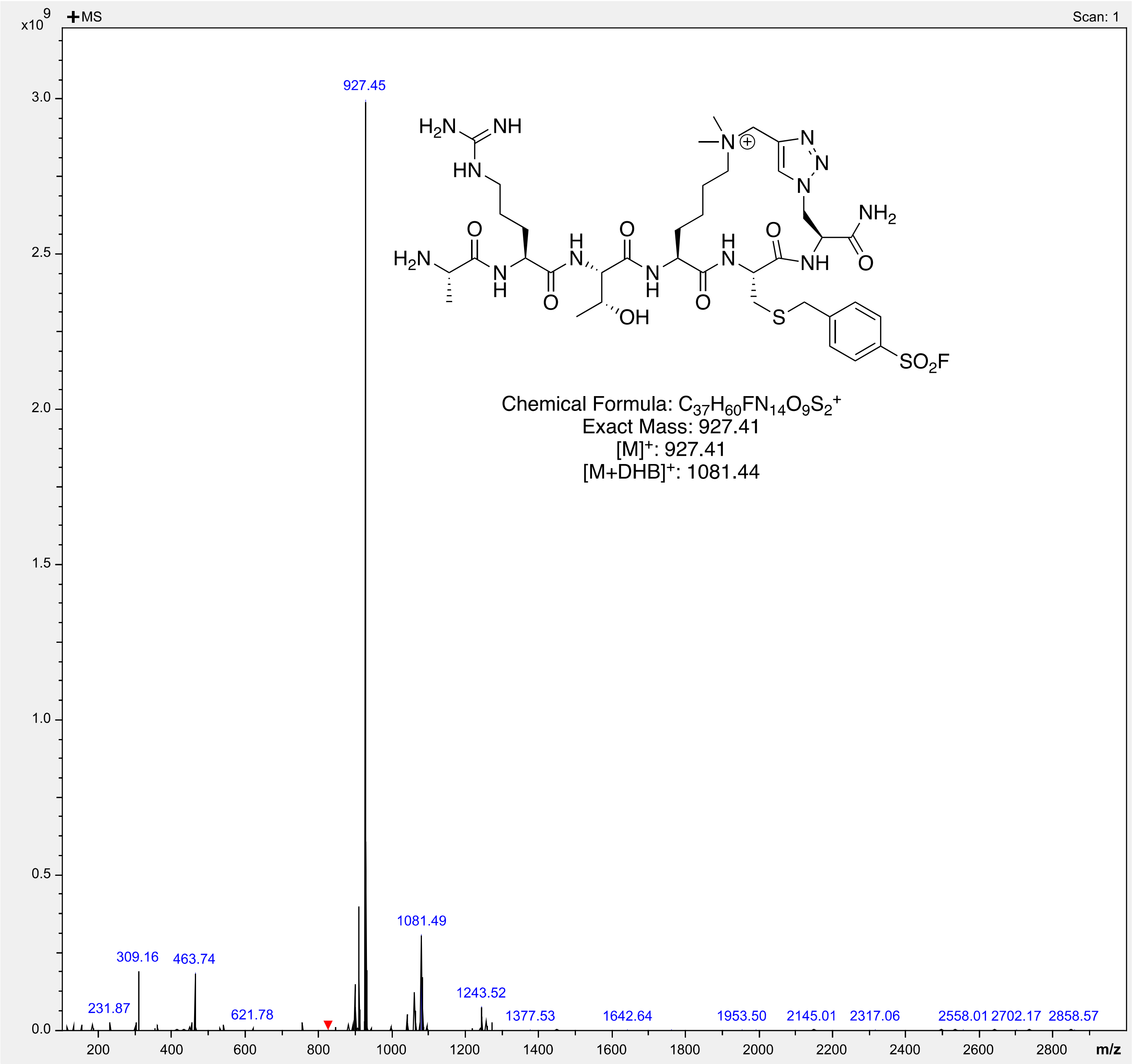

x10^9
+MS
Scan: 1
927.45
309.16
463.74
231.87
621.78
1081.49
1243.52
1377.53
1642.64
1953.50
2145.01
2317.06
2558.01
2702.17
2858.57
m/z
Chemical Formula: $C_{37}H_{60}FN_{14}O_9S_2^+$
Exact Mass: 927.41
$[M]^+$: 927.41
$[M+DHB]^+$: 1081.44

Synthesis of biotinylated covalent thioether cyclopeptide C-36

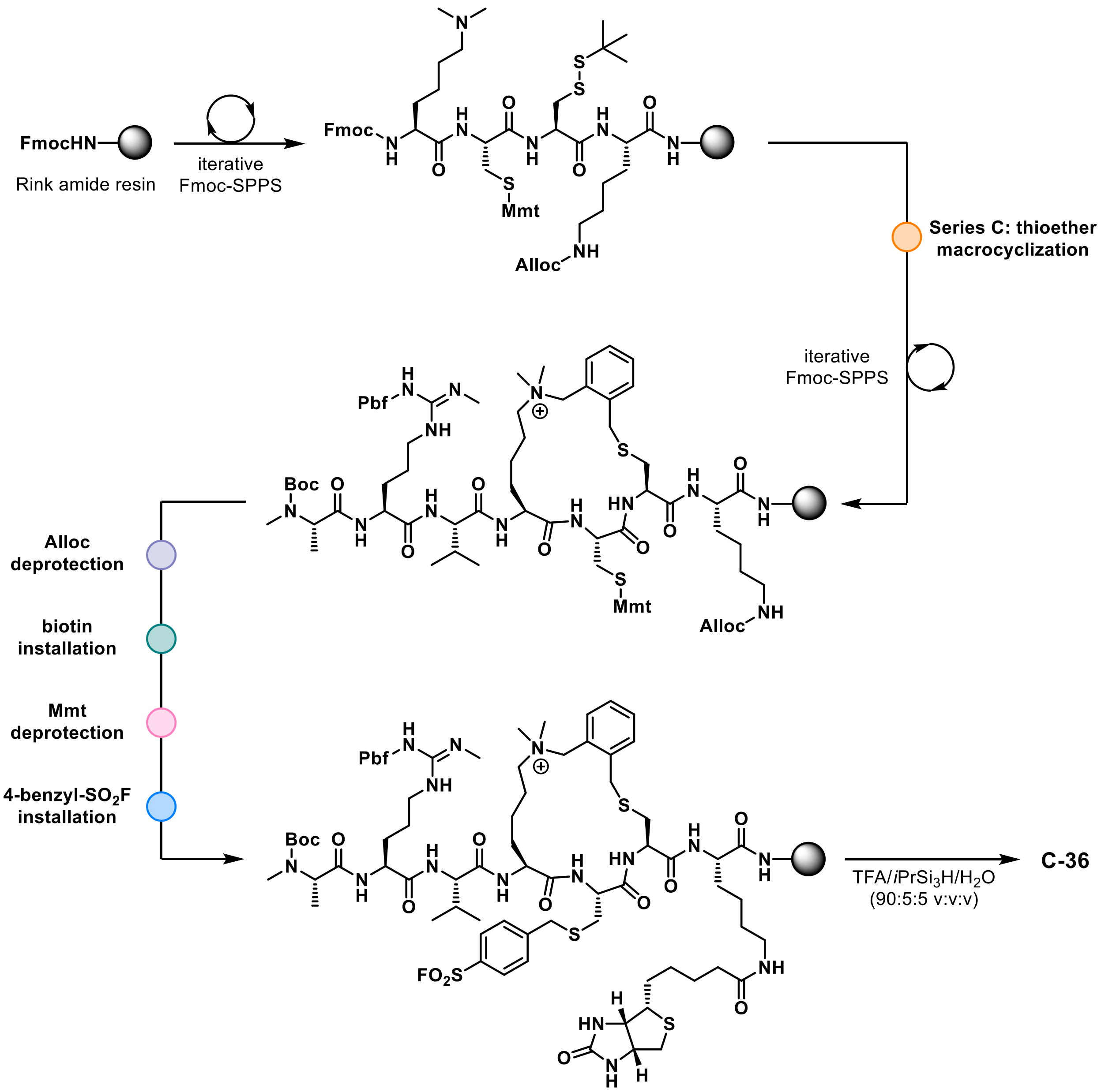

Tetrapeptide Fmoc-Lys(me2)-Cys(Mmt)-Cys(S*t*Bu)-Lys(Alloc)-CONH-Rink amide resin (1 equiv) was prepared on Rink amide resin according to the procedures outlined in the **Iterative Fmoc-SPPS workflow**. The tetrapeptide intermediate was subjected to **Series C: thioether macrocyclization** conditions and elongated using the procedures outlined in the **Iterative Fmoc-SPPs workflow**. The Alloc group was removed using the procedures outlined in **Deprotection procedures**. The resin was treated with a solution of biotin (4 equiv), PyBOP (4 equiv) and DIPEA (8 equiv) in DMF and agitated at room temperature for one hour. The liquid was expelled from the reaction vessel and the resin was washed with DMF (5 × 3 mL), DCM (5 × 3 mL), and DMF (5 × 3 mL). The Mmt group was cleaved using the procedures described in **Deprotection procedures**, and the 4-benzylsulfonyl fluoride functionality was introduced according to the protocols outlined in **Installation of the 4-benzylsulfonyl fluoride covalent warhead**. The resin was washed with DMF (5 × 3 mL), DCM (5 × 3 mL), DMF (5 × 3 mL) and DCM (5 × 3 mL). The peptide was cleaved from resin and purified according to the procedures outlined in the **Iterative Fmoc-SPPS workflow** to afford **C-36**.

Compound **C-36**

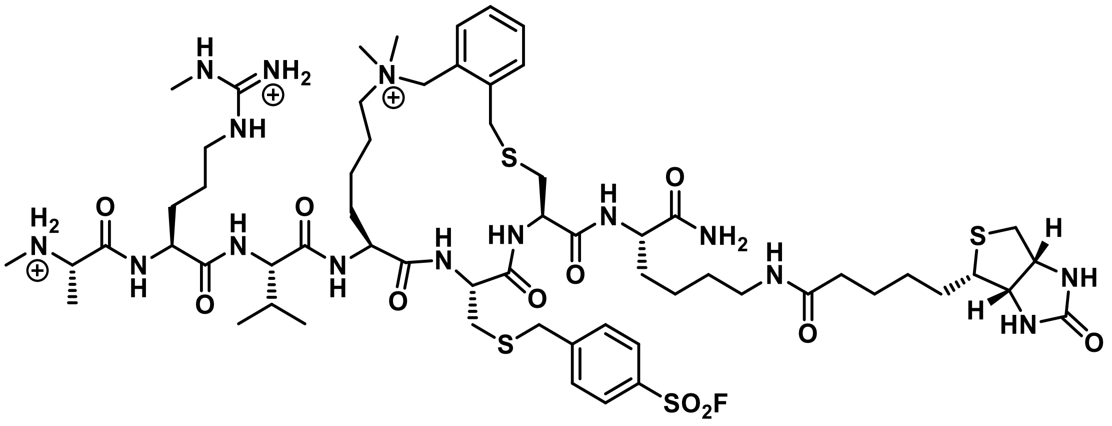

Yield: 9%

Exact mass: 1364.65 $[C_{61}H_{99}FN_{15}O_{11}S_4]^{3+}$

HPLC trace (<u>Method 2</u>):

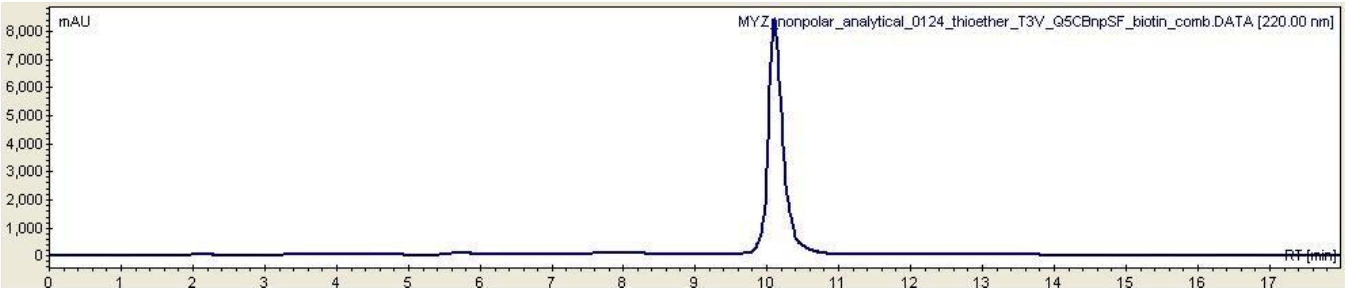

LCMS

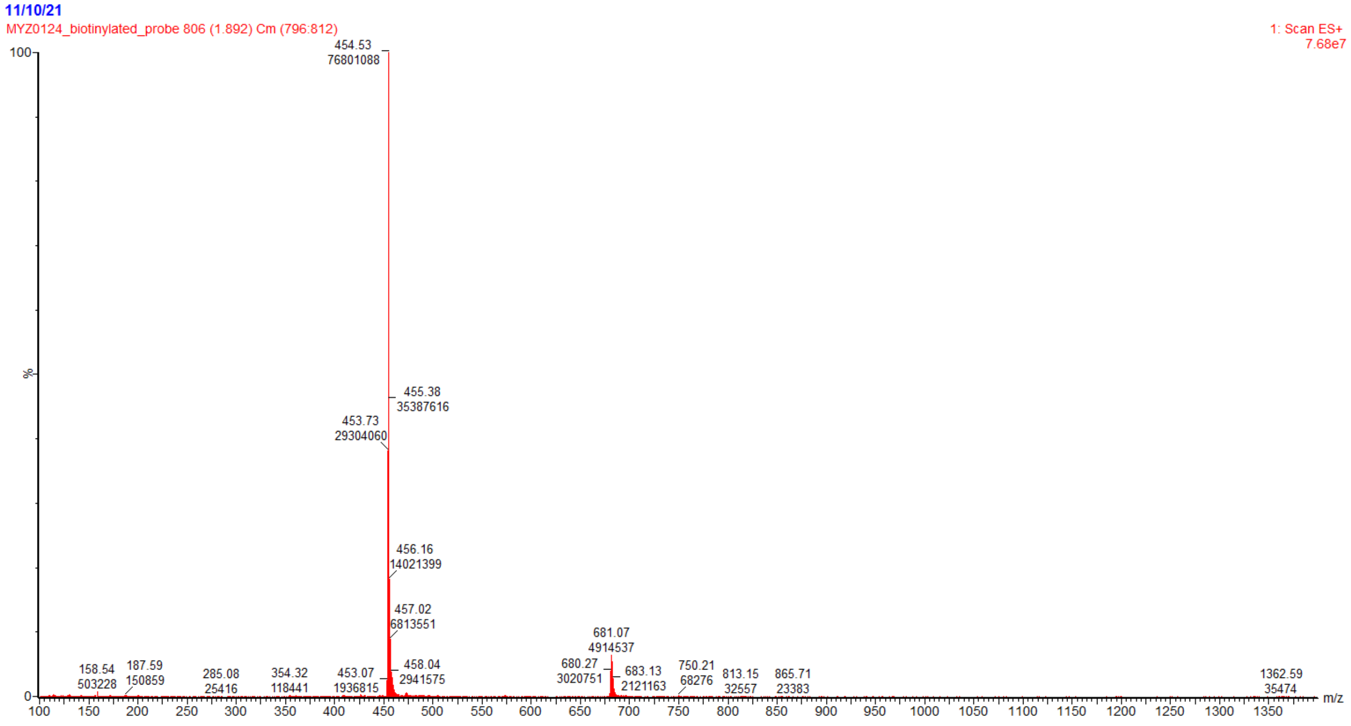

MALDI

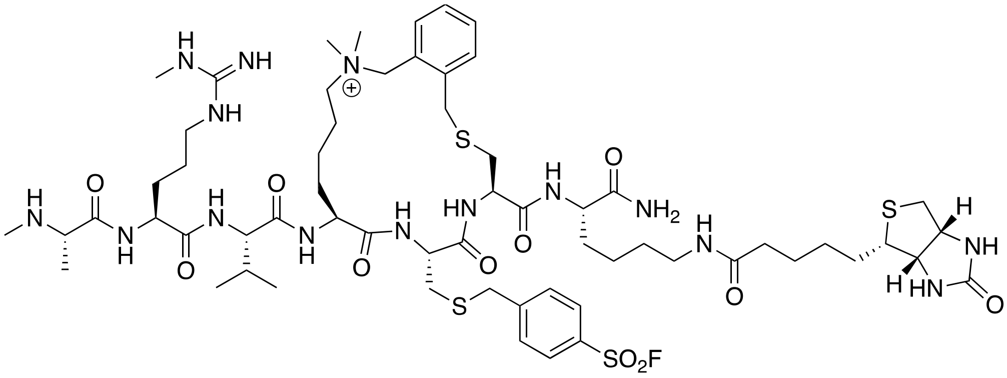

Chemical Formula: $C_{61}H_{97}FN_{15}O_{11}S_4^+$
Exact Mass: 1362.64
$[M]^+$: 1362.64
$[M+DHB]^+$: 1516.67

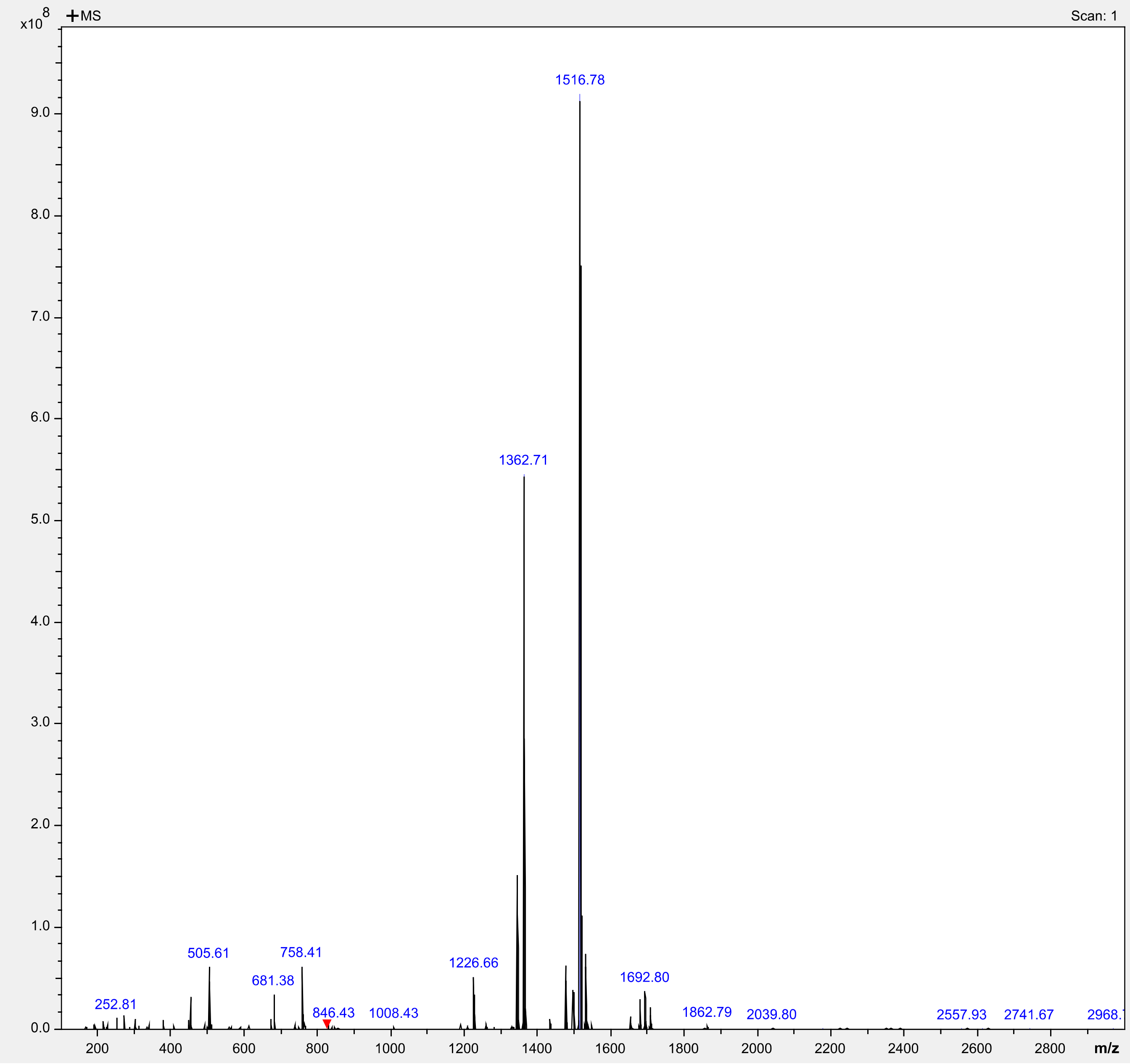

Expression and purification of His$_6$-MBP-PHD3, WT and mutant PHD3

Wild-type His$_6$-MBP-PHD3 and mutant PHD3 constructs (K1620A, K1622A and K1620A/K1622A) were cloned into a pET His$_6$ MBP TEV vector and expressed in BL21(DE3) *E. coli.* cells. Expression and purification of WT and mutant PHD3 followed the same protocol. Cells were induced with 0.3 mM IPTG and grown at 18 °C overnight. Collected pellets were flash-frozen in liquid nitrogen and stored at -80 °C until further use. The pellets were suspended in lysis buffer (20 mM Tris pH 8.0, 0.5 M NaCl, 10% glycerol, 2.5 mM BME, 50 µM $ZnCl_2$, 1x Pierce^{TM} Protease Inhibitor Tablet, EDTA-free), lysed by sonication and centrifuged. The clarified lysate was purified using immobilized metal ion affinity chromatography (HisTrap^{TM} Fast Flow 5 mL), washed with high salt buffer (20 mM Tris pH 8.0, 1 M NaCl, 2.5 mM BME, 50 µM, 20 mM imidazole), followed by low salt buffer (20 mM Tris pH 8.0, 50 mM NaCl, 2.5 mM BME, 50 µM, 20 mM imidazole), and His-MBP-PHD3 was eluted with low salt buffer with supplemented with increasing imidazole concentration (20 → 200 mM imidazole concentration). Collected protein fractions were combined and dialyzed at 4 °C overnight into storage buffer (25 mM HEPES pH 7.5, 50 mM KCl, 5 mM BME, 50 µM $ZnCl_2$). The sample was further purified by size-exclusion chromatography using a HiLoad 26/60 Superdex 75 gel filtration column in storage buffer. Eluted protein fractions were collected, concentrated, aliquoted and flash-frozen in liquid nitrogen.

During the dialysis step, cleavage of the MBP tag with TEV protease afforded the WT PHD3 construct and other related PHD3 mutants.

Fluorescence polarization assays

The binding of His_6-MBP-PHD3 to H3K4me3 WT and mutant peptides, as well as the binding of recombinant His_6-MBP-PHD3 lysine mutant proteins to H3K4me3 WT peptide were measured using direct or competitive fluorescence-polarization (FP) assays.[1] For a direct FP binding assay, 10 nM of C-terminal 5FAM-labelled H3K4me3 10mer peptide was incubated at room temperature for 30 min with varying concentrations of recombinant His_6-MBP-PHD3 protein. Results from direct FP assays were fitted to **equation 1** to deduce K_d values.

$$\textbf{equation 1}\text{: } \mathrm{FP_{obs}} = \frac{\mathrm{FP_{max}}[\mathrm{PHD3}] + \mathrm{FP_{min}}K_\mathrm{d}}{K_\mathrm{d} + [\mathrm{PHD3}]}$$

For competitive FP binding assays, 100 nM of His_6-MBP-PHD3 was incubated with 10 nM of C-terminal 5FAM-labelled H3K4me3 10mer peptide, where varying concentrations of mutant peptides were added as competitors. Results from competitive FP assays were fitted to **equation 2** to deduce K_i values for each mutant competitor peptide.

$$\textbf{equation 2}\text{: } \mathrm{FP_{obs}} = \frac{K_\mathrm{i}(\mathrm{FP_{max}}[\mathrm{PHD3}] + \mathrm{FP_{min}}K_\mathrm{d}) + \mathrm{FP_{min}}K_\mathrm{d}[\mathrm{I}]}{K_\mathrm{i}(K_\mathrm{d} + [\mathrm{PHD3}]) + K_\mathrm{d}[\mathrm{I}]}$$

FP_{obs} is the observed fluorescence polarization, FP_{max} is the maximum FP value, FP_{min} is the minimum FP value, [PHD3] is the concentration of His_6-MBP-PHD3, K_d is the dissociation constant, K_i is the inhibition constant of the competitor peptides and [I] is the concentration of the competitor peptides.

In vitro PHD3 labelling reactions

All covalent labelling reactions were performed in 50 mM HEPES pH 7.5, 50 mM KCl. Wild type of mutant PHD3 (10 µM) and covalent peptide (50 µM) were incubated at 37 °C. At each time point, an aliquot was taken and analyzed by LC-MS. The ESI mass spectra were deconvoluted using MassEnt software.

Cell lysate labelling experiments

Human embryonic kidney 293T cells were cultured in Dulbecco's modified eagle medium (DMEM), high glucose supplemented with 10% fetal bovine serum (FBS), 1% Pen-Strep at 37 °C in a humidified incubator with 5% CO_2 atmosphere. HEK293T cells were grown in a T150 flask to 30,000,000 cells, at which time cells were trypsinized, collected, washed with phosphate-buffered saline twice and flash-frozen in liquid nitrogen. Thawed cell pellets were treated with lysis buffer (50 mM HEPES pH 7.5, 50 mM KCl, 4x Pierce^{TM} Protease Inhibitor EDTA-free). Protein concentration was normalized to 5 mgmL^{-1} using the Bradford assay. Cell lysates were aliquoted and treated with biotinylated thioether probe **C-36** and/or recombinant His$_6$-MBP-PHD3 protein (t = 4.5 h at RT) at which time reactions were quenched by flash-freezing in liquid nitrogen. Thawed samples were treated with 4x SDS buffer and boiled for 10 minutes.

Samples were resolved by 4-20% SDS-PAGE gel (Bio-Rad) and were transferred to a 0.2 µM nitrocellulose membrane using a Trans-Blot Turbo transfer system (Bio-Rad, 7-minute mixed MW protocol). The membrane was briefly stained with Ponceau S solution and washed with TBST buffer (3 × 5 minutes). The membrane was blocked with 1x TBS 1% Casein Blocker (Bio-Rad, Cat. #1610782) for 1 h at RT and incubated with NeutrAvidin-HRP antibody (Thermo Scientific^{TM} Pierce^{TM} High Sensitivity NeutrAvidin^{TM}-HRP Prod # 31030, 1:10,000) for 1 h at RT. The membrane was subsequently washed 4 × 10 minutes with TBST buffer and treated with chemiluminescent western blotting substrate (Amersham^{TM} ECL^{TM} Prime Western Blotting Detection System) for five minutes, then imaged with a Bio-Rad ChemiDoc Molecular Imager.

References

(1) Longbotham, J. E.; Chio, C. M.; Dharmarajan, V.; Trnka, M. J.; Torres, I. O.; Goswami, D.; Ruiz, K.; Burlingame, A. L.; Griffin, P. R.; Fujimori, D. G. Histone H3 Binding to the PHD1 Domain of Histone Demethylase KDM5A Enables Active Site Remodeling. *Nat. Commun.* **2019**, *10* (1), 94. https://doi.org/10.1038/s41467-018-07829-z.

(2) Turner, R. A.; Oliver, A. G.; Lokey, R. S. Click Chemistry as a Macrocyclization Tool in the Solid-Phase Synthesis of Small Cyclic Peptides. *Org. Lett.* **2007**, *9* (24), 5011–5014. https://doi.org/10.1021/ol702228u.

(3) Zhou, H.; Mukherjee, P.; Liu, R.; Evrard, E.; Wang, D.; Humphrey, J. M.; Butler, T. W.; Hoth, L. R.; Sperry, J. B.; Sakata, S. K.; Helal, C. J.; am Ende, C. W. Introduction of a Crystalline, Shelf-Stable Reagent for the Synthesis of Sulfur(VI) Fluorides. *Org. Lett.* **2018**, *20* (3), 812–815. https://doi.org/10.1021/acs.orglett.7b03950.
